# Unraveling Chronic Pain: From Mechanisms and Risks to Diagnosis and Treatment

**DOI:** 10.1002/mco2.70685

**Published:** 2026-03-28

**Authors:** Xiaofeng Dai, Chongxiang Wang, Ping Jiang, Xiaopeng Mei

**Affiliations:** ^1^ National Local Joint Engineering Research Center for Precision Surgery & Regenerative Medicine The First Affiliated Hospital of Xi'an Jiaotong University Xi'an Jiaotong University Xi'an China; ^2^ Department of Pain Medicine The First Affiliated Hospital of Xi'an Jiaotong University Xi'an Jiaotong University Xi'an China; ^3^ Department of Pain Medicine Sichuan Provincial People's Hospital University of Electronic Science and Technology of China Chengdu China

**Keywords:** chronic pain, cold atmospheric plasma, hippocampus, nociceptive pain, neuropathic pain, nociplastic pain

## Abstract

Chronic pain is a globally prevalent and complex condition, encompassing three primary subtypes, that is, nociceptive, neuropathic, and nociplastic, each with distinct biopsychosocial mechanisms. Chronic pain was historically viewed as a monolithic symptom and managed with opioid‐centric models, causing widespread therapeutic failure. While recognition of its heterogeneity has driven a paradigm shift toward precision medicine, tailoring multimodal strategies to the dominant pain mechanism, critical challenges persist. These include difficulty in identifying treatable root causes, limited long‐term efficacy of therapies, and significant side‐effect burdens. To address these gaps, this review systematically synthesized contemporary knowledge, predominantly from the last decade, on the molecular mechanisms, risk factors, diagnostic frameworks, and therapeutic modalities for chronic pain, framed by its pathophysiological subtypes. Furthermore, it explored two novel frontiers aimed at advancing personalized pain medicine. First, it proposed cold atmospheric plasma as an innovative therapeutic intervention capable of modulating key molecular pathways underlying diverse pain manifestations. Second, it introduced an original neurobiological model positing the hippocampus as a putative sensor for nociplastic pain, interfacing with higher‐dimensional information fields. These insights may offer transformative potential for refining diagnostic and therapeutic strategies, potentially revolutionizing the management of chronic pain.

## Graphical abstract

Chronic pain arises through distinct molecular pathways categorized into nociceptive, neuropathic, and nociplastic types. Nociceptive pain begins with TRP channel activation in peripheral nociceptors, signaling via Aδ‐ and C‐fibers through the spinal dorsal horn and spinothalamic tracts to the brain, regulated by descending inhibition and involving both peripheral and central sensitization. Neuropathic pain follows nerve injury, featuring aberrant signaling in Aδ‐ and C‐fibers, central sensitization, and loss of inhibition, without peripheral tissue injury. Nociplastic pain involves central sensitization without structural damage, driven by glial activation, dysregulated descending control, and stress–axis dysfunction. While all types involve inflammation, their mechanisms differ: nociceptive pain is dominated by abnormal nociceptors, neuropathic by both abnormal transduction and CNS reorganization, and nociplastic primarily by CNS reorganization. We propose a hippocampal model, where peripheral nociceptors sense tissue injury for nociceptive pain, the hippocampus acts as a central sensor for nociplastic pain, potentially processing higher‐dimensional experiences, and neuropathic pain is viewed as abnormal signal transduction; all converge in the hypothalamus for integration before cortical conscious experience. Cold atmospheric plasma (CAP), rich in reactive oxygen and nitrogen species, may relieve chronic pain through five mechanisms: healing physical injury to reduce tissue inflammation and nociceptive pain; restoring nerve damage to suppress neuroinflammation and neuropathic pain; targeting pain‐related comorbidities; modulating FTO for improved obesity management; and crosstalking with estrogen receptors to regulate hormonal signaling. While the first three address pain sources, the latter two implicate molecular pathways like FTO and ER. Thus, CAP may act as a multimodal intervention targeting all three chronic pain types.

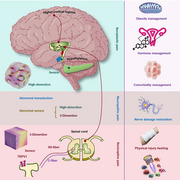

## 1 Introduction

Chronic pain was defined as “an unpleasant sensory and emotional experience associated with, or resembling that associated with, actual or potential tissue damage and lasts or recurs for longer than 3 months” by the International Association for the Study of Pain (IASP) [1]. Unlike acute pain, which serves a critical biological alarm function, chronic pain often becomes maladaptive, losing its protective purpose and instead promoting suffering and disability. Chronic pain represents one of the most pervasive and debilitating medical challenges worldwide, transcending its role as a mere symptom to become a complex disease state on its own [2]. Chronic pain has been affecting a significant portion of the population and has imposed a substantial burden on individuals, healthcare systems, and society. According to a chronic pain survey conducted across 15 primary care centers involving 5438 random interviews, the overall prevalence was estimated to be 22% [3].

Historically, chronic pain was, fundamentally, misunderstood as a monolithic symptom secondary to peripheral injury or disease, leading to a simplistic treatment model. The clinical approach was largely anchored in broad‐spectrum pharmacological suppression, with a heavy reliance on opioids for long‐term management, disregarding the central role of the brain in pain processing and modulation. This resulted in widespread therapeutic inadequacy and high rates of adverse effects, cementing chronic pain as a persistently debilitating challenge. Current advances in chronic pain have recognized it not as a simple disease but a highly heterogeneous syndrome encompassing at least three distinct subtypes based on their biopsychosocial nature, that is, nociceptive, neuropathic, and nociplastic pain. While nociceptive pain mainly results from tissue injury or inflammation [4], neuropathic pain largely originates from nerve damage or diseases within the somatosensory nervous system [5, 6], and nociplastic pain predominantly refers to pain that originates from altered nociceptive processing in the absence of clear evidence of tissue damage or neural injury [7]. Accordingly, the clinical paradigm has been decisively shifted from the one‐size‐fits‐all model to a precision medicine framework, where therapeutic strategies are tailored to the patient's dominant biopsychosocial feature. This is exemplified by the use of specific drug classes, for example, nonsteroidal anti‐inflammatory drugs (NSAIDs) for nociceptive pain [8] and antidepressants or anticonvulsants for neuropathic pain [9]. Also, the concept of multimodal therapy, which combines pharmacological treatments with physical therapy, psychological therapy, and interventional procedures, has become the cornerstone of modern pain management. For instance, treatment combining medications, patient education, and graded aerobic exercise has been applied in conditions like fibromyalgia, a representative nociplastic pain state [10].

Despite significant progresses made in medical research and treatment modalities, the effective management of chronic pain continues to be inconsistently implemented within the healthcare systems worldwide [11]. A primary challenge is the frequent inability to pinpoint a clear, treatable root cause for chronic pain, leading to symptomatic rather than curative management. This is compounded by diagnostic difficulties, as many chronic pain conditions, especially those with a nociplastic component, lack definitive confirmatory tests and rely on clinical diagnosis [12]. Consequently, treatments still often fail to address the underlying pathology and may be ineffective, with evidence showing that even with comprehensive drug therapy, complete pain relief has been achieved only in a minority of patients and relapse frequently occurs. Furthermore, all major pharmacotherapies carry substantial side‐effect burdens that limit their long‐term use. For instance, while NSAIDs increased risks of gastrointestinal bleeding, cardiovascular events, and kidney problems [13, 14], opioids were associated with tolerance, dependence, and respiratory depression [15], and even newer nonopioid drugs like gabapentinoids showed dose‐dependent increases in adverse events [16, 17]. This underscores the persistent significant gaps in translating pathophysiological understanding into consistently effective, personalized therapies across healthcare systems. This makes it pertinent to critically synthesize current knowledge on each type of chronic pain and, critically, explore emerging frontiers setting structured path toward advanced personalized pain medicine.

Motivated by these considerations, this paper focuses on the key mechanisms, risk factors, diagnostic frameworks, and evidence‐based treatment paradigms, mapped by pathophysiological subtyping, which define the contemporary understanding and management of chronic pain. Importantly, this paper proposes cold atmospheric plasma (CAP), the fourth state of matter [18, 19, 20, 21, 22], as a novel therapeutic intervention for chronic pain, leveraging its unique ability to interact with key molecular pathways and risk factors that underlie diverse chronic pain manifestations. In addition, we propose a model in which the hippocampus acts as a putative sensor for nociplastic pain by interfacing with higher‐dimensional information fields, while the hypothalamus decodes these signals for conscious interpretation that offers transformative potential for refined theranostic strategies in nociplastic pain. Our systematic synthesis and original insights may revolutionize current theranostic instruments for improved management of chronic pain.

## 2 Clinical Prevalence of Chronic Pain

Chronic pain represents a pervasive and complex global health challenge, imposing a substantial burden on individuals, healthcare systems, and societies worldwide. Its reported prevalence exhibits remarkable geographic variability, ranging from an estimated 2 to over 40% among adult populations across different nations and studies. This introductory overview sets the stage for a detailed exploration of the distinct prevalence, clinical characteristics, and mechanistic underpinnings of each pain subtype, highlighting the imperative for mechanism‐informed approaches to diagnosis and treatment.

**FIGURE 1:**
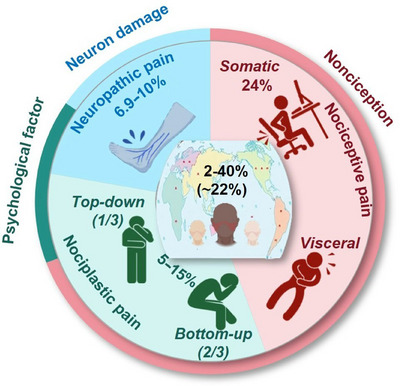
Chronic pain classification and epidemiological prevalence. Chronic pain can be categorized into three types: nociceptive, neuropathic, and nociplastic. Global prevalence varies widely, ranging from 2 to over 40% across populations and an averaged prevalence of around 22%. Nociceptive pain, a protective response to tissue damage, can be subdivided into somatic and visceral subtypes, with the somatic subtype being common in conditions like osteoarthritis that affects around 24% of US adults and precise estimates being challenging due to its symptomatic presentation. Neuropathic pain, caused by somatosensory system damage, affects 6.9–10% of people worldwide and involves maladaptive sensitization, often complicating low back pain or surgery recovery. Nociplastic pain, marked by altered pain processing without clear tissue or nerve injury, affects 5–15% of the population and can be classified into “top‐down” and “bottom‐up” subtypes. While the top‐down subtype is peripheral‐driven and accounts for approximately 1/3 of nociplastic pain, the bottom‐up subtype is central‐maintained and represents around 2/3 of the inflicted cohort.

Chronic pain exhibits a highly variable estimated prevalence among adults ranging from 2 to over 40% [3, 23, 24, 25, 26, 27, 28, 29, 30, 31, 32, 33, 34] (Figure 1). In the United States, the estimated prevalence of chronic pain among adults was 20.4% based on a National Health Interview Survey [27, 28]. In Italy, a cross‐sectional population survey of nearly 1300 adults revealed a chronic pain prevalence of 28.4% [29]. In France, the prevalence of chronic pain was 31.7% according to a large mail survey involving nearly 25,000 respondents [30]. In the United Kingdom, a pooled estimate of chronic pain prevalence among adult residents was 43.5%, with the 95% CI being 38.4–48.6% [31]. The prevalence of chronic pain in Europe was estimated to be 26% according to a European study on pain involving 3849 phone calls and 300 in‐depth interviews [32]. In Morocco, a survey of 5328 individuals found that 21% interviewees (95% CI: 19.9–22.2%) reported chronic daily pain [33]. In Australia, chronic pain was reported by 17.1% of males and 20.0% of females according to 17543 completed interviews [24]. In Japan, the prevalence of chronic pain was estimated to be 39.3% according to a survey of 6000 adults [34]. Additionally, a meta‐analysis including 28 low‐ and middle‐income countries reported the onset of chronic pain among 34% of adults [23].

Chronic pain can be systematically classified using several complementary frameworks. One approach is based on the primary anatomical location of the pain, leading to designations such as musculoskeletal, visceral, or orofacial pain. Alternatively, it can be categorized by underlying etiology into primary pain (e.g., fibromyalgia, where pain is the core disease) and secondary pain (resulting from an underlying condition such as cancer or diabetic neuropathy). Notably, chronic pain can be classified into nociceptive, neuropathic, and nociplastic pain from a mechanistic perspective to help address the complex biopsychosocial nature of the condition for targeted treatment.

Nociceptive pain arises from the activation of high‐threshold peripheral sensory neurons, known as nociceptors, in response to intense mechanical, chemical, or thermal noxious stimuli that typically subsides once the noxious stimulus is removed. It serves as a protective physiological function by signaling the presence, location, intensity, and duration of actual or potential tissue damages [4]. Nociceptive pain can be further divided into somatic and visceral pain. While somatic pain stems from peripheral tissues such as skin, muscle, and bone, visceral pain originates from internal organs or within the abdominal cavity [26]. The prevalence of chronic nociceptive pain is difficult to estimate as it is a symptom of countless underlying conditions rather than a disease itself. Yet, being a model of somatic nociceptive pain [35], osteoarthritis (OA) has been estimated to affect 24% of adults in the United States [36]; and visceral nociceptive pain has been recognized as the most frequent form of pain associated with comorbidity and the leading cause for medical consultations [37] (Figure 1).

Neuropathic pain, defined by the IASP as “pain arising from a lesion or disease of the somatosensory nervous system,” constitutes a major clinical challenge with 6.9–10% of the global population being affected [5, 6] (Figure 1). It has been estimated that up to 37% of individuals with low back pain [38, 39] and 40% of people after surgery [40] suffered from neuropathic pain. In contrast to nociceptive pain that is self‐protective, neuropathic pain frequently evolves into a chronic, maladaptive condition characterized by self‐sustaining pathological activity [41, 42]. Its pathophysiology involves diverse mechanisms such as ectopic neuronal discharge, peripheral and central sensitization, dysregulation of ion channels, and disrupted balance between excitatory and inhibitory neurotransmission [43, 44]. These alterations lead to aberrant processing of sensory signals along neuraxial pathways, rendering neuropathic pain typically resistant to conventional analgesics and necessitating mechanism‐targeted therapeutic strategies [45].

The term “nociplastic pain” was proposed as a third mechanistic descriptor of chronic pain in 2016 [7]. However, over a third of chronic pain involves a combination of all three mechanisms where nociplastic pain can be viewed as a component of a chronic pain continuum [46]. For instance, nociplastic mechanisms commonly coexist in nociceptive conditions like rheumatic diseases and chronic low back pain as well as neuropathic disorders such as small fibre neuropathy [47, 48]. Nevertheless, nociplastic pain was estimated to affect the general population at a rate of 5–15%, transcending geographic, demographic, and social boundaries [46, 49] (Figure 1).

In conclusion, chronic pain constitutes a multifaceted global phenomenon with a highly variable yet consistently significant prevalence across diverse populations. As evidenced by epidemiological data from numerous countries, it affects a substantial proportion of adults, underscoring its status as a major public health priority. The mechanistic classification of chronic pain into nociceptive, neuropathic, and nociplastic subtypes provides an essential framework for moving beyond symptomatic management toward pathophysiology‐driven therapeutic strategies. Each category presents distinct clinical challenges: nociceptive pain, often secondary to conditions like OA, signals ongoing tissue pathology; neuropathic pain, affecting a considerable segment of the global population, arises from nervous system dysfunction and is notoriously difficult to treat; and nociplastic pain, frequently coexisting with other mechanisms, involves central sensitization and altered pain processing. Understanding these mechanistic distinctions, along with their overlapping presentations in many chronic pain states, is paramount for developing accurate diagnosis and targeted interventions.

## 3 Molecular and Systems Mechanisms of Chronic Pain

**FIGURE 2:**
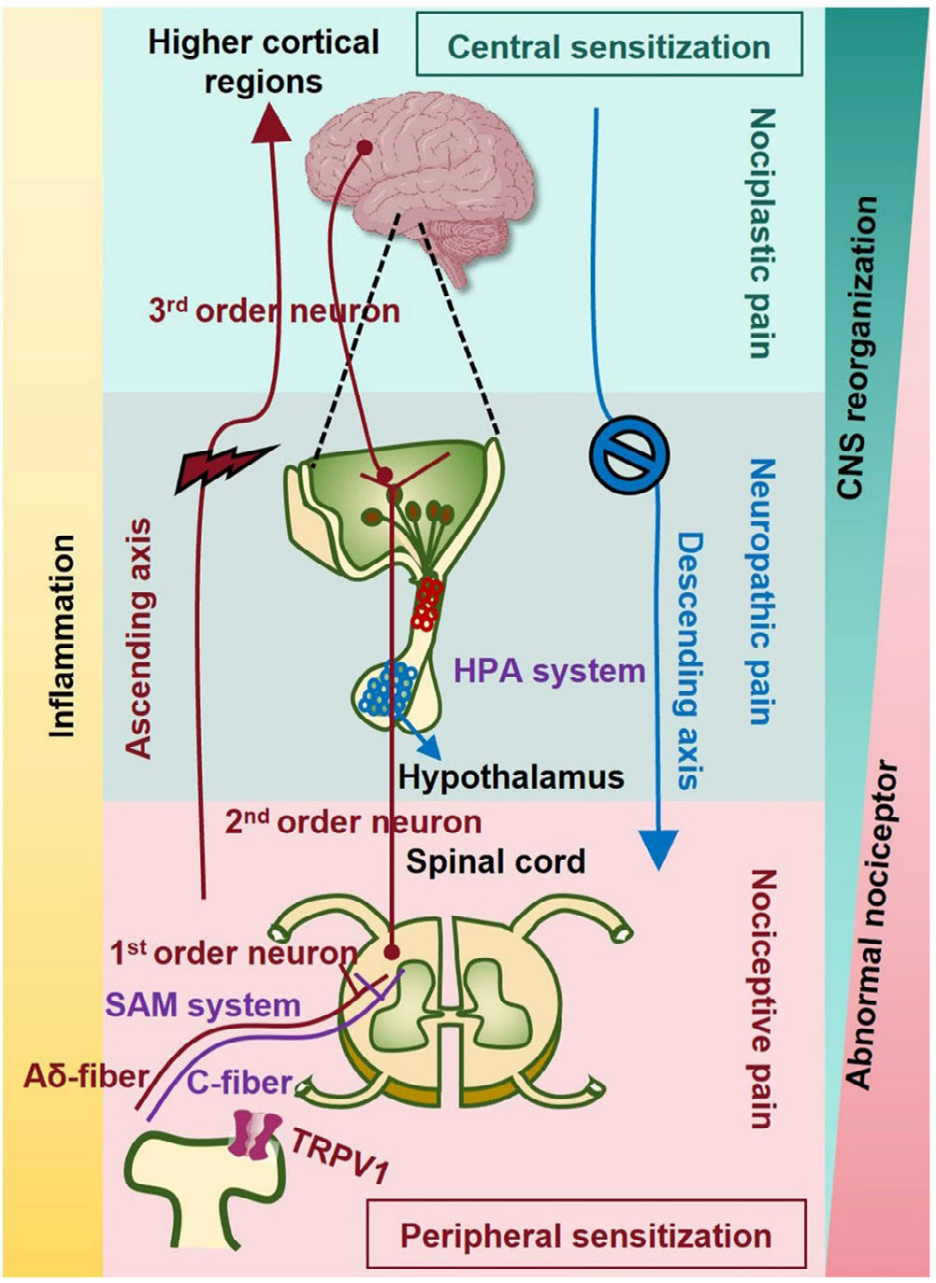
Molecular mechanisms of chronic pain. Chronic pain arises through distinct molecular pathways depending on its type. The basic path is represented by the nociceptive pain, which starts with TRP channel activation in peripheral nociceptors, signaling via Aδ‐ and C‐fibers to the spinal dorsal horn, then ascending through spinothalamic tracts to higher brain regions, regulated by descending inhibition. Neuropathic pain follows nerve injury, featuring central sensitization without non‐nerve peripheral tissue injury and loss of inhibition. Nociplastic pain involves central sensitization without structural damage, driven by glial activation, dysregulated descending control, and stress–axis dysfunction (HPA hypoactivity and SAM hyperactivity). While nociceptive pain involves both central sensitization and peripheral sensitization, neuropathic pain may or may not have central sensitization and peripheral sensitization, and nociplastic pain is featured solely with central sensitization. Overall, all three types of chronic pain are accompanied with inflammation; and the likelihoods of possessing “abnormal nociceptor” and “CNS reorganization” may exhibit an opposite pattern across different types of chronic pain. That is, nociceptive pain may be dominant by abnormal nociceptor, nociplastic pain may more likely involve CNS reorganization, and neuropathic pain may be driven by both.

Chronic pain is not a unitary phenomenon but a heterogeneous condition arising from a complex interplay of diverse and often overlapping pathophysiological mechanisms. These mechanisms range from molecular‐level sensitization in the periphery and central nervous system (CNS) to large‐scale remodeling of brain circuits, culminating in distinct clinical phenotypes classified as nociceptive, neuropathic, or nociplastic pain. This section synthesizes the current scientific understanding of these core processes. It first delineates the foundational concepts of peripheral and central sensitization, which represent the primary amplifiers of nociceptive signaling (Figure 2). It then systematically details the specific mechanistic architectures that define each pain subtype, explaining how tissue injury, nerve damage, and maladaptive central processing translate into unique clinical experiences (Figure 2). Furthermore, it explores the critical modulating roles of neuroendocrine stress axes, such as the hypothalamic–pituitary–adrenal (HPA) and sympatho–adreno–medullary (SAM) systems, and the profound structural and functional reorganization within key brain regions like the hippocampus and prefrontal cortex (PFC) (Figure 2). Together, these interconnected mechanisms provide a comprehensive blueprint for understanding how acute, protective pain can transition into a persistent, debilitating disease state, forming the essential biological basis for all subsequent discussion of diagnosis and therapeutics.

### 3.1 Peripheral and Central Sensitization

#### 3.1.1 Peripheral Sensitization

Peripheral sensitization is a process of increased responsiveness and reduced activation threshold of nociceptors (primary sensory neurons) in the periphery to stimulation of their receptive fields, initiated directly by tissue injury, inflammation, or infection [50] (Figure 2).

The molecular cornerstone of this phenomenon is the local release of a diverse array of inflammatory mediators from damaged cells, immune cells (e.g., mast cells, macrophages), and the vascular endothelium, collectively termed the “inflammatory soup.” This includes protons (H^+^), bradykinin, prostaglandins (PGE_2_), serotonin (5‐HT), adenosine triphosphate, nerve growth factor (NGF), and proinflammatory cytokines such as tumor necrosis factor‐alpha (TNF‐α), IL‐1β, and IL‐6. These mediators act on specific receptors (e.g., G‐protein‐coupled receptors, tyrosine kinase receptors, ion channels) expressed on the terminals of nociceptors, triggering intracellular signaling cascades that converge on the modulation of key transducing and voltage‐gated ion channels. A pivotal target is the transient receptor potential vanilloid 1 (TRPV1) channel, which is sensitized by mediators like bradykinin and NGF, lowering its thermal activation threshold and increasing its response to capsaicin and protons [51, 52]. Simultaneously, the tetrodotoxin‐resistant voltage‐gated sodium channels Nav1.8 and Nav1.9 are upregulated and their kinetics altered, promoting membrane depolarization and repetitive firing. This biochemical milieu leads to a phenotypic switch in the nociceptor, characterized by spontaneous ectopic activity, a lowered threshold for activation (resulting in primary hyperalgesia), and an augmented response to suprathreshold stimuli. Importantly, this process is dynamic and can be maintained by transcriptional changes within the dorsal root ganglion (DRG), where the cell bodies of nociceptors upregulate the production of pronociceptive peptides like substance P and calcitonin gene‐related peptide (CGRP), further fueling neurogenic inflammation upon peripheral release.

Peripheral sensitization thus serves as the initial critical amplifier of nociceptive signals, providing the persistent drive necessary for inducing more durable changes within the CNS [50].

#### 3.1.2 Central Sensitization

Central sensitization, defined by the IASP as “increased responsiveness of nociceptive neurons in the CNS to their normal or subthreshold afferent input” [53], represents a fundamental form of synaptic plasticity that underlies chronic pain hypersensitivity (Figure 2). This neuroplasticity amplifies pain signals and involves complex interactions between ascending pain signal transduction pathways, descending modulatory systems, and psychological factors [54, 55, 56, 57, 58]. The conceptual foundation was established by the seminal discovery that peripheral tissue injury induces a state of hyperexcitability in spinal cord nociceptive neurons [59], a finding subsequently validated and elaborated across diverse animal models demonstrating enhanced responsiveness within central pain pathways [60].

This core mechanism is classically initiated and maintained by intense, repeated, or sustained nociceptive barrage from peripherally sensitized C‐fibers, leading to an excessive presynaptic release of glutamate and neuropeptides like substance P and CGRP within the spinal dorsal horn. The pivotal postsynaptic event is the activity‐dependent removal of the magnesium block from N‐methyl‐d‐aspartate (NMDA) receptors, permitting substantial calcium influx upon glutamate binding. This calcium surge acts as a critical second messenger, activating downstream kinase cascades, including protein kinase C (PKC), calcium/calmodulin‐dependent protein kinase II, and mitogen‐activated protein kinases (MAPKs), which phosphorylate ion channels and receptors. Key consequences of this phosphorylation include the enhanced synaptic trafficking and function of α‐amino‐3‐hydroxy‐5‐methyl‐4‐isoxazolepropionic acid (AMPA) receptors, augmenting fast excitatory transmission, and a further increase in NMDA receptor responsiveness itself. These molecular alterations collectively reduce the activation threshold and expand the receptive fields of second‐order wide‐dynamic‐range neurons, enabling normally subthreshold inputs from low‐threshold Aβ mechanoreceptors to gain access to and activate nociceptive circuits. This neuronal transformation clinically manifests as secondary hyperalgesia (increased pain from noxious stimuli in uninjured tissue surrounding the lesion) and tactile allodynia (pain provoked by innocuous light touch). Importantly, central sensitization is not a passive phenomenon but is dynamically regulated and amplified by a loss of inhibitory tone due to impaired glycinergic and GABAergic interneuronal function (disinhibition) and by the activation of spinal microglia and astrocytes, which release proinflammatory cytokines and other neuroactive substances that perpetuate the hyperexcitable state [61].

The translational relevance of this mechanism has been firmly established through the development of human experimental models, which consistently demonstrate that clinical pain conditions are associated with quantifiable alterations in indices of central sensitization [61]. This self‐sustaining state of central hyperexcitability, capable of outlasting the initial peripheral trigger, forms a key pathophysiological basis for a wide spectrum of chronic pain conditions [62].

### 3.2 Pain‐Type Specific Mechanisms

#### 3.2.1 Nociceptive Pain

The molecular and neural architecture of nociceptive pain is initiated by the activation of specialized peripheral sensory neurons, or nociceptors, which are functionally categorized and widely distributed throughout somatic and visceral tissues [63, 64, 65, 66, 67]. This classification, encompassing high‐threshold mechanoreceptors, thermal, chemical, polymodal, and silent nociceptors, is not merely descriptive but reflects a fundamental principle of nociceptive coding, that is, the translation of specific, potentially damaging stimulus modalities into distinct neural signals. Critically, the phenomenon of silent nociceptors, which remain quiescent under normal conditions but become activated and mechanosensitive in the presence of inflammatory molecules, underscores a pivotal mechanism underlying pathological pain states such as arthritis. This transition from a silent to an active state represents a profound plastic change, effectively lowering the pain threshold and contributing to hypersensitivity following tissue injury, thereby illustrating a direct molecular link between peripheral pathology and altered sensory perception [63, 64, 65, 66, 67].

The initial transduction event occurs at specialized, unsheathed free nerve endings, which act as biological signal converters [68, 69, 70]. These terminals express a polymodal array of receptor channels, most notably members of the transient receptor potential family, which depolarize the first‐order neuron in response to noxious thermal, mechanical, or chemical stimuli. This depolarization initiates action potentials that are then propagated by two anatomically and functionally distinct primary afferent fiber types, that is, the lightly myelinated Aδ‐fibers and the unmyelinated C‐fibers. This structural and physiological dichotomy is fundamental to pain perception. Aδ‐fibers, with their rapid conduction velocities and small receptive fields, mediate the initial, sharp, well‐localized “first pain” that signals the presence, location, and nature of an acute injury. In contrast, C‐fibers, with their slow conduction and broad receptive fields, convey the subsequent, dull, aching, and poorly localized “second pain,” which encodes the sustained intensity and aversive quality of the stimulus [68, 69, 70]. The cell bodies of these neurons reside in the dorsal root or trigeminal ganglia, and their central terminals project into the spinal cord dorsal horn, predominantly targeting Rexed laminae I and II. Here, a critical neurochemical divergence occurs, that is, Aδ‐fibers primarily release the fast‐acting excitatory neurotransmitter glutamate onto second‐order neurons, while C‐fibers often corelease glutamate alongside neuropeptides like substance P and CGRP. This divergence lays the foundational framework for parallel processing pathways, one geared toward rapid sensory discrimination and another toward sustained affective and autonomic integration [68, 69, 70].

The complexity of central pain processing is embodied in the three major ascending pathways originating from the spinal dorsal horn [68, 69, 70]. The neospinothalamic tract, arising primarily from nociceptive‐specific neurons in lamina I, represents the direct line for sensory‐discriminative processing. Its axons decussate and ascend in the contralateral lateral spinothalamic tract, synapsing in the ventroposterolateral and ventroposterior inferior nuclei of the thalamus. Thalamocortical projections then relay this information to the primary and secondary somatosensory cortices, enabling the precise localization, intensity discrimination, and temporal analysis of the painful stimulus. This pathway is complemented by an analogous trigeminal pathway for facial pain, which synapses in the ventroposteromedial thalamic nucleus. In stark contrast, the paleospinothalamic and archispinothalamic tracts originate from deeper laminae (II, IV–VIII) and subserve the affective‐motivational and autonomic dimensions of pain. These phylogenetically older pathways project diffusely and often bilaterally via the anterior spinothalamic tract and other multisynaptic routes to the brainstem reticular formation, periaqueductal gray (PAG), and the intralaminar thalamic nuclei, particularly the parafascicular and centromedian nuclei. From these subcortical hubs, information is widely disseminated to limbic structures (including the ACC and insula), the hypothalamus, and brainstem autonomic nuclei. This network generates the unpleasant, aversive quality of pain, drives associated emotional responses (e.g., fear, anxiety), and coordinates autonomic and endocrine reactions, such as increased heart rate and stress hormone release. The existence of these parallel pathways explains how pain can be both a precise sensory event and a profound emotional experience, and why these components can be dissociated under certain clinical or pharmacological conditions [68, 69, 70].

This elaborate ascending transmission is not unchecked but is subject to robust, dynamic endogenous modulation via descending inhibitory systems [55]. A key neuromodulatory framework is the endogenous opioid system. Opioid receptors (mu, delta, kappa) are densely distributed both presynaptically on primary afferent terminals in spinal laminae I–V and postsynaptically on second‐order neurons, as well as throughout critical supraspinal sites including the PAG, rostral ventral medulla (RVM), locus coeruleus, and various limbic regions. Endogenous ligands like enkephalins, beta‐endorphins, and dynorphins activate these Gi/o‐protein‐coupled receptors, leading to neuronal hyperpolarization through increased potassium conductance and inhibition of presynaptic calcium influx. This effectively suppresses the release of pronociceptive neurotransmitters (e.g., glutamate, substance P) and dampens neuronal excitability at the first central synapse. The descending pain modulatory circuit is organized as a hierarchy, with the PAG serving as a pivotal midbrain integrator. The PAG receives inputs from forebrain limbic areas and, in turn, activates downstream nuclei like the nucleus raphe magnus in the RVM and the locus coeruleus. These nuclei send serotonergic and noradrenergic projections, respectively, back down to the spinal dorsal horn. Operating largely through direct opioid receptor activation and/or by disinhibiting local inhibitory interneurons (e.g., by suppressing GABAergic inhibition), this system can selectively attenuate C‐fiber‐mediated pain transmission while often sparing A‐fiber function. This selective filtering allows for the suppression of prolonged, pathological pain states without completely obliterating acute protective nociception. Moreover, this circuit is not static; its activity is powerfully influenced by cognitive factors (attention, expectation), emotional state (stress, fear), and contextual cues, positioning it as a central mechanism through which top‐down processes shape the ultimate pain experience [55].

In synthesis, the mechanism of nociceptive pain reveals a profoundly integrated, multilayered system. It begins with modality‐specific transduction and dual‐channel peripheral transmission (Aδ/C‐fibers), which segregates information at the earliest possible stage. This segregation is maintained and elaborated within the spinal cord through distinct neurochemical coding and then funneled into parallel, functionally specialized ascending pathways, that is, a direct, fast lemniscal‐like pathway for sensory detail and indirect, slow, polysynaptic pathways for affective and autonomic integration. Pervading this entire architecture is a state‐dependent, opioid‐rich descending modulatory network that exerts dynamic, context‐sensitive control over synaptic transmission. Therefore, pain is not a monolithic sensation but an emergent perceptual state, constructed from the interactive synthesis of discriminative, affective, cognitive, and modulatory components. This integrated model explains the multifaceted nature of pain experience, and provides a critical framework for understanding both its protective function in acute settings and its maladaptive persistence in chronic pathological conditions.

#### 3.2.2 Neuropathic Pain

Neuropathic pain arises not merely from passive damage to the somatosensory nervous system, but from a cascade of active, maladaptive plastic changes that establish a self‐perpetuating state of aberrant signaling across peripheral and central pathways [54, 57, 58, 71]. Its initiation and maintenance represent a fundamental failure of homeostasis, where compensatory molecular and cellular responses to injury paradoxically evolve into the core drivers of the pathological pain state. The process is typically instigated by a lesion at any level of the neuraxis, be it peripheral nerve, DRG, or nerve root, resulting from etiologies as diverse as compression, metabolic dysfunction (e.g., diabetic neuropathy), infection, or trauma [54, 57, 58, 71]. This initial insult sets in motion a bidirectional and interactive pathology between the peripheral and CNSs, breaking down the traditional boundaries between stimulus and response.

The primary pathology often originates at the site of peripheral nerve injury, where a profound molecular reorganization occurs within the damaged axons. A critical maladaptation is the dysregulation of ion channels, specifically the pathological upregulation and altered trafficking of voltage‐gated sodium channels (e.g., Nav1.3, Nav1.7, Nav1.8) and N‐type calcium channels [54, 57, 58, 71]. This shift results in abnormal sodium and calcium influx, rendering the axons hyperexcitable and generating spontaneous ectopic discharges that act as a persistent false signal of ongoing tissue damage. Importantly, nerve injury is not an isolated neuronal event but triggers a robust immune‐neuronal interface. Resident and infiltrating immune cells, particularly macrophages, release a cocktail of proinflammatory cytokines, including TNF‐α, IL‐1β, and IL‐6, as well as growth factors like NGF [54, 57, 58, 71]. This inflammatory milieu does not remain localized; it actively sensitizes adjacent intact nociceptors, lowering their activation threshold and amplifying their response to normal stimuli, a process termed peripheral sensitization. Thus, the initial focal injury seeds a broader field of hyperexcitability, creating a pathogenic peripheral drive that relentlessly bombards the CNS.

This sustained peripheral barrage is the critical driver for the development of central sensitization, a state of hyperexcitability within the dorsal horn of the spinal cord that represents the cardinal neural mechanism of neuropathic pain [54, 57, 58, 71]. Excessive and persistent glutamate release from hyperactive primary afferents leads to the intense and prolonged activation of postsynaptic NMDA receptors on second‐order neurons. This NMDA receptor activation, normally blocked by magnesium ions under resting conditions, allows a massive influx of calcium. The elevated intracellular calcium acts as a second messenger, triggering a cascade of kinase pathways, including PKC, protein kinase A, and MAPKs, which phosphorylate ion channels and receptors, thereby increasing neuronal excitability and synaptic strength. This is complemented by a parallel failure of inhibitory controls. There is a documented loss of GABAergic and glycinergic inhibitory interneuronal function, sometimes via selective neuronal apoptosis or a shift in anion gradients that renders GABAergic transmission excitatory. Furthermore, the balance of descending modulation from the brainstem shifts from net inhibition to net facilitation, with pathways from the RVM promoting, rather than suppressing, dorsal horn excitability. This combination of heightened excitation and diminished inhibition creates a central amplifier for nociceptive signals.

The convergence of these peripheral and central maladaptive processes, ectopic firing, peripheral sensitization, central synaptic potentiation, disinhibition, and descending facilitation, culminates in the defining clinical phenomena of neuropathic pain [54, 57, 58, 71]. Hyperalgesia (an exaggerated response to a normally painful stimulus) and allodynia (pain from a nonpainful stimulus such as light touch) are direct clinical manifestations of this heightened gain throughout the somatosensory system. The phenomenon of spontaneous pain, occurring without any external trigger, is a testament to the autonomous, self‐sustaining nature of the established neural circuit pathology. This complex, multimechanistic pathogenesis underpins the notorious resistance of neuropathic pain to conventional analgesics like opioids and NSAIDs, which primarily target nociceptive transmission rather than the maladaptive neural plasticity and immune signaling that are now central to the condition. Effective therapeutic strategies, therefore, must move beyond simple blockade of neurotransmission and instead aim to modify disease processes, such as ion channel regulation, immune modulation, and the restoration of inhibitory tone, to disrupt the self‐sustaining cycle of neuropathic pain.

#### 3.2.3 Nociplastic Pain

Nociplastic pain represents a distinct mechanistic entity within the pain taxonomy, characterized not by structural nerve damage (neuropathic) nor by ongoing tissue injury (nociceptive), but by a functional dysregulation and pathological amplification of pain processing within the CNS. Its core is the maladaptive phenomenon of central sensitization, a state of hyperexcitability in spinal and supraspinal neurons that amplifies responses to both noxious and innocuous inputs. Critically, this sensitization is sustained by a self‐reinforcing neuroimmune–glial axis. Microglia and astrocytes become chronically activated by various triggers, including prior intermittent pain, chronic stress, or immune challenges, and transition into a proinflammatory state [56, 72]. These cells release a cascade of signaling molecules, including cytokines (e.g., IL‐1β, TNF‐α), chemokines, and other neuromodulators. This glial output profoundly alters the synaptic environment in the dorsal horn. It enhances NMDA receptor function, increases the expression and release of pronociceptive neuropeptides like substance P and CGRP, and fosters long‐term potentiation (LTP) of pain‐signaling synapses. This creates a feed‐forward loop where neural activity drives glial activation, which in turn further amplifies neural excitability, thereby cementing the sensitized state independent of a peripheral driver.

Concurrently, there is a profound dysregulation of the brain's endogenous pain modulatory systems. The balance between descending inhibition and facilitation is disrupted. There is a documented reduction in the efficacy of inhibitory pathways originating from the brainstem, particularly those mediated by norepinephrine and 5‐HT from the locus coeruleus and rostral ventromedial medulla. In parallel, facilitatory pathways are potentiated, often involving neurotransmitters like cholecystokinin (CCK), which antagonizes opioidergic signaling [56]. This shift results in a net loss of inhibitory tone and a gain of facilitatory drive, effectively removing the “brakes” on and pressing the “accelerator” for pain transmission at the spinal level. This central disinhibition and facilitation are further compounded by neuroendocrine dysfunction (e.g., alterations in HPA axis function) and altered signaling of neurotrophins like brain‐derived neurotrophic factor (BDNF), which can promote synaptic plasticity and hyperexcitability. The integrated outcome of these mechanisms, that is, enhanced synaptic transmission, glial‐mediated inflammation, and unbalanced descending control, is the clinical triad of widespread pain, hyperalgesia, and allodynia, hallmarks of conditions such as fibromyalgia and chronic widespread pain syndromes.

The pathophysiology of nociplastic pain is now understood to exhibit significant mechanistic heterogeneity, leading to a clinically and neurobiologically meaningful distinction between two primary subtypes, that is, “bottom‐up” and “top‐down” nociplastic pain [56]. This framework is crucial for understanding prognosis and treatment responsiveness. The “bottom‐up” subtype aligns with the classical model of activity‐dependent central sensitization. Here, persistent, albeit often low‐grade, peripheral nociceptive input (e.g., from OA, tendinopathy) acts as the primary driver, initiating and maintaining central amplification. A key prognostic feature is that this amplification can often normalize upon removal of the peripheral drive, as the central changes remain contingent on that input [56, 59]. In stark contrast, the “top‐down” subtype is primarily sustained by central mechanisms that operate largely autonomously from the periphery. These mechanisms include cognitive‐affective processes (e.g., catastrophizing, hypervigilance, emotional distress), genetic predispositions affecting CNS pain processing, and learned pain memories. In this subtype, the central sensitization becomes an engrained, self‐sustaining state, independent of any ongoing peripheral nociception [56].

This theoretical distinction is powerfully validated by clinical and epidemiological data. In OA patients with a nociplastic pain component undergoing total joint arthroplasty, surgical removal of the presumed peripheral source led to pain resolution in approximately two‐thirds of patients, consistent with a dominant bottom‐up mechanism. However, one‐third of patients derived no benefit, suggesting their pain was sustained by entrenched top‐down mechanisms, rendering peripheral intervention ineffective [73]. Furthermore, large‐scale genetic epidemiological research provides a developmental perspective. A study of over 25,000 individuals revealed that early‐onset nociplastic pain (<50 years old) exhibited a heritability approximately three to four times greater than later‐onset cases (>60 years old) [74]. This strongly suggests that early‐onset cases are more likely mediated by top‐down mechanisms, driven by a stronger genetic/neurobiological vulnerability to central dysregulation. Conversely, later‐onset cases show stronger “bottom‐up” characteristics, where accumulated peripheral insults over a lifetime play a more prominent role in triggering central changes in a less predisposed nervous system [74].

In synthesis, nociplastic pain is a disorder of central pain processing, defined by glial‐mediated neuroinflammation, synaptic hyperefficacy, and a failure of descending modulation. The critical advancement lies in recognizing its bi‐axial nature. The bottom‐up axis represents a peripherally driven, input‐dependent sensitization, often amenable to treatments targeting the periphery or general CNS depressants. The top‐down axis represents a centrally engrained, input‐independent state, likely requiring interventions that directly target central plasticity, cognitive‐affective factors, and specific neuroimmune pathways. This integrated model moves beyond a monolithic view of central sensitization, offering a mechanism‐based framework for subtyping patients, predicting treatment outcomes, and developing targeted therapies.

### 3.3 Neuroendocrine and Stress Axes

A characteristic neuroendocrine dysfunction, marked by hypoactivation of the HPA axis and concomitant hyperactivation of the SAM axis, is a critical pathophysiological driver of chronic pain. This imbalance fosters a state of maladaptive allostasis, characterized by inadequate anti‐inflammatory cortisol signaling and excessive catecholamine‐driven central sensitization, peripheral inflammation, and sympathetic dysregulation. This pathway underlies the development and maintenance of diverse chronic pain states, including nociplastic, neuropathic, and persistent nociceptive pain.

#### 3.3.1 SAM Axis

The SAM axis serves as a critical molecular mediator in the development and maintenance of various chronic pain states, with varied roles across nociceptive, neuropathic, and nociplastic pain.

In nociceptive pain, sustained SAM activation contributes to peripheral sensitization through the release of catecholamines (e.g., norepinephrine and epinephrine), which promote proinflammatory cytokine production and enhance nociceptor excitability at the site of injury. This facilitates the transition from acute to chronic pain.

In neuropathic pain, SAM dysregulation often manifests as sympathetic hyperactivity, which can directly drive pain via aberrant sympathetic‐sensory coupling, particularly after nerve injury. Elevated catecholamines exacerbate neuroinflammation and central sensitization, further amplifying pain signaling within the somatosensory nervous system.

In nociplastic pain, SAM hyperactivation is a hallmark feature, frequently co‐occurring with HPA axis dysfunction. Here, catecholamines facilitate widespread central sensitization and dysregulate descending inhibitory pathways, contributing to chronic pain in the absence of clear tissue or neural damage. The resulting state of autonomic imbalance reinforces a maladaptive cycle of stress and pain, commonly seen in conditions such as fibromyalgia or irritable bowel syndrome (IBS).

Across all three types, chronic pain acts as a persistent stressor, further dysregulating the SAM axis and creating a treatment‐resistant vicious cycle of stress‐pain amplification.

#### 3.3.2 HPA Axis

The HPA axis plays a distinct yet interconnected role across nociceptive, neuropathic, and nociplastic pain states, reflecting varied pathophysiological mechanisms.

In nociceptive pain, acute activation of the HPA axis serves a protective role. Stress‐induced cortisol release helps suppress inflammation and temporarily attenuates pain signaling. However, if nociceptive input becomes chronic, prolonged HPA activation may eventually lead to dysregulation.

In neuropathic pain, the HPA axis often shows inconsistent alterations, sometimes hyperactive initially, then becoming hypoactive over time. Nerve injury acts as a persistent stressor, disrupting glucocorticoid receptor signaling and feedback inhibition. This dysregulation can exacerbate neuroinflammation and central sensitization, contributing to chronicity and treatment resistance.

In nociplastic pain, HPA axis hypoactivation is a hallmark feature. Conditions such as fibromyalgia are characterized by flattened diurnal cortisol rhythms, low baseline cortisol, and blunted responses to stress [75]. Reduced glucocorticoid signaling leads to inadequate control of proinflammatory processes and loss of endogenous analgesic effects, which sustains central sensitization and promotes widespread pain in the absence of clear tissue damage. This dysfunction forms a vicious cycle, that is, while chronic pain amplifies stress, stress axis dysregulation in turn intensifies pain perception that is often accompanied by a greater symptom burden, fatigue, and comorbid mood disorders.

Across all types, HPA axis involvement underscores the integral relationship between stress neurobiology and pain chronification, with nociplastic pain showing the most consistent pattern indicating hypo‐activated HPA axis.

### 3.4 Brain Circuit Remodeling

Brain circuit remodeling, particularly within the hippocampus, PFC, and the broader limbic system, represents a fundamental neuroplastic process underlying learning, memory, emotional regulation, and the pathophysiology of several psychiatric and chronic pain disorders. This remodeling encompasses structural and functional adaptations, including synaptic LTP and depression, dendritic spine reorganization, neurogenesis, and alterations in network‐wide oscillatory synchrony.

The hippocampus, a central hub for memory formation and spatial navigation, demonstrates remarkable structural plasticity, most notably through the continuous birth and integration of new neurons in the dentate gyrus throughout life, a process influenced by environmental enrichment, exercise, and stress. Functionally, hippocampal circuits undergo activity‐dependent synaptic changes, primarily at the Schaffer collateral‐CA1 synapse, where NMDA receptor‐mediated calcium influx triggers signaling cascades that lead to the persistent strengthening of synaptic connections, forming the cellular basis of declarative memory [76, 77, 78]. Critically, the hippocampus does not operate in isolation; it is densely interconnected with the PFC and the amygdala within the limbic system. The PFC, essential for executive functions such as decision‐making, working memory, and top‐down emotional control, exhibits remodeling through experience‐dependent changes in synaptic efficacy and spine density in layers II/III and V pyramidal neurons, particularly along the hippocampal–PFC pathway [79, 80]. Theta‐gamma cross‐frequency coupling between these regions is crucial for memory consolidation and retrieval [81]. The limbic system, integrating the hippocampus, amygdala, anterior cingulate cortex (ACC), and insula, coordinates emotional responses [82].

In conditions of chronic stress or pain, maladaptive remodeling occurs. Hippocampal volume and neurogenesis can decrease [83, 84], while the amygdala and ACC show increased synaptic strength and neuronal excitability, strengthening fear and aversive memory circuits. Concurrently, a weakening of prefrontal inhibitory control over these limbic structures, mediated by dendritic atrophy and reduced connectivity, leads to a loss of top‐down regulation, resulting in negative affective states, cognitive deficits, and the persistence of pain memory [85, 86, 87]. This triad of hippocampal impairment, limbic hyperexcitability, and prefrontal disinhibition constitutes a core pathological circuit remodeled in chronic pain and mood disorders, highlighting the interplay between structural plasticity and functional network dyssynchrony.

In summary, the persistence of chronic pain is governed by a multilayered and dynamic pathophysiological hierarchy. The process is often initiated at the periphery through sensitization of nociceptors but is critically cemented within the CNS via enduring synaptic potentiation and a loss of inhibitory control, a state known as central sensitization. This core mechanism manifests distinctly across the pain spectrum. In nociceptive pain, it is driven by ongoing tissue injury; in neuropathic pain, it is fueled by aberrant signaling from damaged nerves; and in nociplastic pain, it becomes a self‐sustaining, input‐independent disorder of central processing. This maladaptive plasticity is further exacerbated and maintained by a dysregulated stress response, characterized by HPA axis hypoactivity and SAM axis hyperactivity, which disrupts the body's homeostatic and anti‐inflammatory controls. Ultimately, these molecular and systemic changes catalyze a profound remodeling of key brain circuits, including hippocampal impairment, limbic hyperexcitability, and a weakening of prefrontal cortical modulation. This collective dysfunction transforms pain from a transient sensory signal into a deeply ingrained, multidimensional experience encompassing sensory, emotional, cognitive, and autonomic dimensions. Understanding this integrated mechanistic cascade, from molecule to circuit, is therefore paramount for developing targeted, effective, and personalized therapeutic strategies aimed at reversing these pathological processes rather than merely masking the final symptom.

## 4 Risk Factors

**FIGURE 3:**
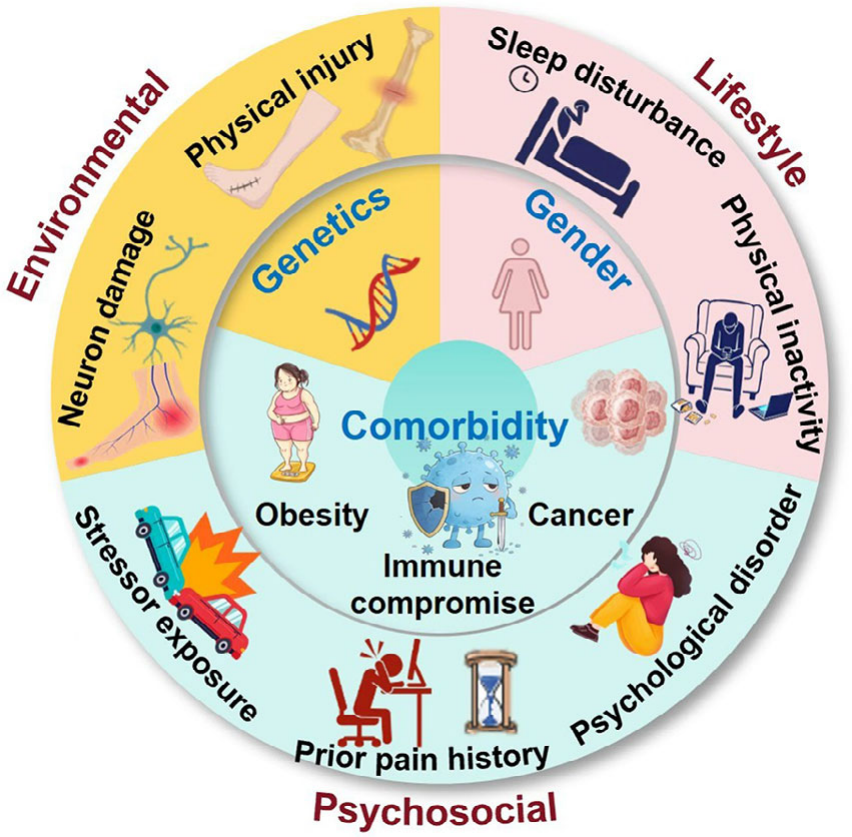
Risk factors of chronic pain. Integrated biopsychosocial model of risk factors for chronic pain, categorizing determinants into intrinsic and extrinsic domains and illustrating their dynamic interplay in pain chronification. Intrinsic factors encompass biological vulnerabilities: genetic factors, gender‐related factors (e.g., female predominance), and comorbidity‐related factors (e.g., diabetes, obesity, cancer, immune compromise). Extrinsic factors represent external influences, encompassing environmental factors (e.g., physical injury, neuron damage), lifestyle factors (e.g., physical inactivity, sleep disturbance), and psychosocial factors (e.g., prior pain history, stressor exposure across the lifespan, and psychological disorders). This model illustrates how these intrinsic vulnerabilities and extrinsic exposures interact synergistically to initiate and facilitate persistent chronic pain, intrinsic and extrinsic factors are integrated within concentric cycles, where the outer ring of extrinsic influences continuously interacts with the core of intrinsic vulnerabilities.

Chronic pain is a multifaceted and debilitating condition that arises from a complex interplay of biological, psychological, and social determinants. Its pathophysiology and clinical presentation are shaped by a diverse range of risk factors, which can be broadly categorized into intrinsic and extrinsic domains. Intrinsic factors encompass inherent biological vulnerabilities, including genetic predispositions, gender‐related differences, and comorbid systemic diseases that alter pain processing from within the organism. Extrinsic factors involve external influences that act upon the individual, such as environmental exposures, lifestyle choices, and psychosocial stressors (Figure 3). This section provides a detailed analysis of these critical determinants, synthesizing current evidence to elucidate their individual and synergistic roles in initiating, amplifying, and perpetuating the transition from acute nociception to persistent chronic pain. Understanding this intricate risk factor landscape is essential for developing effective prevention strategies, personalized therapeutic interventions, and a comprehensive biopsychosocial model of chronic pain management.

### 4.1 Intrinsic Factors

#### 4.1.1 Genetic Factors

Genetic susceptibility significantly contributes to the risk of developing chronic pain [88, 89], with rare monogenic disorders such as congenital insensitivity to pain with anhidrosis, erythromelalgia, and paroxysmal extreme pain disorder being directly caused by mutations in single genes [90, 91]. Through genome‐wide association studies and molecular genetic investigations, polymorphisms in 28 genes have been significantly associated with neuropathic pain. Among these reported genetic variants, 75% were noncoding with gene regulatory roles with approximately 40% being located within the enhancer regions [92]. The implicated genes are primarily involved in key biological processes such as neurotransmission, immune response, and metabolism.

Representative chronic pain predisposing genes involved in neurotransmission include *PRKCA* (encoding PKCα), *OPRM1* (encoding opioid receptor Mu 1), *COMT* (encoding catechol‐O‐methyltransferase), *SCN9A* (encoding sodium voltage‐gated channel alpha subunit 9), *SLC6A4* (encoding solute carrier family 6 member 4 protein or 5‐HT transporter [5‐HTT]), and *CACNG2* (encoding the transmembrane AMPA receptor regulatory protein gamma‐2). Specifically, the rs887797 variant in *PRKCA* showed a significant association with neuropathic pain in total joint replacement patients (*p* = 4.29E−06), supported by meta‐analysis of two UK cohorts and the Rotterdam study (OR = 2.41, 95% CI: 1.74–3.34, *p* = 1.29×E−7 under a recessive model) [93]. The A118G variant of *OPRM1* was associated with reduced neuropathic pain risk in diabetic foot ulcer patients (OR = 0.24, 95% CI: 0.07−0.80, *p* = 0.038) [94]. The Met allele of *COMT* rs4680 polymorphism was significantly associated with neuropathic pain in sclerosis patients (*p* = 0.046) [95] and with pain severity in sciatica [40]. For *SCN9A*, four variants (rs7449889, rs3750904, rs4369876, rs12478318) were associated with painful diabetic peripheral neuropathy, with two (rs4369876 and rs12478318) also correlating with pain intensity [96]. In *SLC6A4*, the 5‐HTTLPR short‐short genotype was more frequent in trigeminal neuralgia patients of East Asian ancestry (OR = 5, 95% CI: 1.45–3.17, *p* = 0.034) and linked to greater pain severity [97]. Finally, the A–C–C *CACNG2* haplotype (rs4820242–rs2284015–rs2284017) was associated with persistent postsurgical pain after breast surgery (OR = 1.65, *p* = 0.02), though neuropathic characteristics were not confirmed [98]

Genes involved in the immune response, particularly human leukocyte antigens (HLA‐A, HLA‐B, HLA‐DRB1, and HLA‐DQB1), have been implicated in chronic pain susceptibility. For example, HLA‐A*33 and HLA‐B*44, as well as the HLA‐DRB1*1302 haplotype, have been positively associated with postherpetic neuralgia (PHN) in a Japanese population [99], a finding corroborated in a Korean study that identified HLA‐B*44 as the most strongly associated allele along with additional risk alleles including HLA‐B*13, ‐B*15, DRB1*10.01, and DRB1*1202 [100]. Similarly, HLA‐DRB1*04 and HLA‐DQB1*03:02 have been significantly linked to persistent postsurgical pain [101]. Meta‐analyses consolidated these findings, revealing that neuropathic pain, primarily PHN and persistent postsurgical pain, was associated with HLA‐DRB1*13 (OR = 1.59, 95% CI: 1.04–2.41, *p* = 0.031) [100, 101, 102, 103], HLA‐DRB1*04 (OR = 1.40, 95% CI: 1.02–1.93, *p* = 0.03), HLA‐A*33 (OR = 2.41, 95% CI: 1.72–3.39, *p* = 3.9×E−7) [100, 102, 103, 104], and HLA‐DQB1*03 (OR = 2.86; 95% CI: 1.57–5.21, *p* = 6×E−4) [104], while HLA‐A*02 exerted a protective effect (OR = 0.60; 95% CI: 0.41–0.90, *p* = 0.01) [100, 102, 103]. In addition, polymorphisms in genes encoding cytokines such as IL6, IL10 and TNF‐α have been significantly associated with multiple neuropathic pain conditions. For instance, the *IL6* variant rs13306435 (T15A) has been significantly linked to neuropathic pain (rs13306435; OR = 4.4; CI: 1.2‐5.7; *p* = 1.1×E−02), and haplotype analysis of four *IL6* SNPs (rs1800797; G‐597A, rs1800796; G‐572C, rs1800795; G‐174C, and rs13306435; T15A) further revealed a strong association between the GGGA haplotype and neuropathic pain (OR = 5.4, 95% CI: 1.5–9.2, *p* = 3.3×E−03) [105]. The rs11674595 in *IL1R2* (OR = 36.07, 95% CI: 2.02–643.37, *p* = 0.01) and the CGCGATT haplotype from rs3024505–rs3024496–rs1878672–rs1518111–rs1518110–rs3024491 in *IL10* remained significantly associated with neuropathic pain after covariate adjustment (OR = 0.21, 95% CI: 0.05–0.91, *p* = 3×E−02) [106]. Additionally, the *TNF‐α* variant rs1800629 was associated with an increased risk of neuropathic pain following hernia surgery (OR = 1.93, 95% CI: 1.03–3.61; *p* = 3.6×E−02) [107]

From the perspective of metabolism, polymorphisms in *GCH1*, a gene encoding GTP cyclohydrolase, have been associated with altered susceptibility to neuropathic pain. In studies of human immunodeficiency virus (HIV)‐associated sensory neuropathy in individuals of black African ethnicity, two protective haplotypes correlated with reduced neuropathic pain risk were identified, which were a 3‐SNP haplotype (CAT, *p* = 0.02) and a 6‐SNP haplotype (CTCGAT, *p* = 0.04) [108]. Additionally, the C allele of rs8007267 in *GCH1* was significantly associated with persistent postsurgical pain (*p* = 0.02) [109]. Additionally, genetic variants in iron metabolism‐related pathways have been implicated in neuropathic pain susceptibility and severity [110]. According to a study involving 168 HIV‐associated sensory neuropathy patients from an admixed American population, 17 variants across eight metabolic genes, that is, *ACO1* (encoding aconitase 1), *B2M* (encoding beta‐2‐microglobulin), *BMP6* (encoding bone morphogenetic protein 6), *FXN* (encoding frataxin), *CP* (encoding ceruloplasmin)*, TF* (encoding transferrin), *TFRC* (encoding transferrin cell surface receptor), and *SLC11A2* (encoding solute carrier family 11 member 2), were associated with neuropathic pain. In *ACO1*, rs2026739 (OR = 1.5, *p* = 0.007) and rs7033149 (OR = 1.6, *p* = 0.012) increased the risk, while rs4495514 was protective (OR = 0.4, *p* = 0.036). Variants rs16966334 (OR = 2.4, *p* = 0.003) and rs1901531 (OR = 1.6, *p* = 0.028) in *B2M*, as well as rs13072552 (OR = 1.6, *p* = 0.007), rs13075921 (OR = 1.6, *p* = 0.048), and rs3816893 (OR = 1.9, *p* = 0.004) in *CP* also conferred increased risk. *BMP6* polymorphisms showed mixed effects, with rs270388 (OR = 1.3, *p* = 0.05) and rs267206 (OR = 1.4, *p* = 0.03) being risky, and rs267202 (OR = 0.8, *p* = 0.05) being protective. Similarly, while *TF* rs2718796 increased the risk of developing neuropathic pain (OR: 3.1, *p* = 0.007), *TF* rs8177306 (OR = 0.4, *p* = 0.023) reduced it. Protective associations were also observed for *FXN* rs3793451 (OR = 0.4, *p* = 0.047), *SLC11A2* rs224446 (OR = 0.7, *p* = 0.047), and the T allele of *TFRC* rs480760 (OR = 0.6, *p* = 0.004).

In addition, numerous causal genetic variants linked to the function of the HPA axis [111], serotonergic signaling [112, 113], and brain structure and functionality [114] have been discovered. These genetic influences operate through interactions with environmental factors, particularly given the importance of early‐life experiences in shaping how genes are expressed and regulated [115].

#### 4.1.2 Gender‐Related Factors

Accumulating evidence has indicated significant sexual dimorphism in chronic pain, with women exhibiting a higher prevalence of chronic pain [116, 117, 118, 119]. Take neuropathic pain as the example, although the symptoms can overlap with those of vitamin D deficiency, a clinical study has demonstrated that neuropathic pain was significantly associated with female gender in Type 2 diabetes mellitus independent of the vitamin D status [119]. Mechanistically, gender‐dependent involvement of central immune cells (microglia) and peripheral adaptive immune cells (T‐cells) has been shown to drive the maladaptive processes underlying neuropathic pain [118]. Such a gender difference also conveys profound pharmaceutical implications. It has been reported that women achieved higher blood concentrations than men during treatment with medications such as amitriptyline, nortriptyline, duloxetine, venlafaxine, and pregabalin, resulting in higher rates of adverse effects. These gender‐related pharmacokinetic and pharmacodynamic differences suggested the necessity of initiating therapy with lower doses in women suffering from neuropathic pain for improved tolerability and safety. Given the pronounced sex‐based variations in drug response, personalized treatment strategies have been advocated to address the fact that up to 60% of neuropathic pain patients failed to achieve adequate pain relief with standard pharmacotherapy [116].

Nociplastic pain conditions also consistently exhibit a female predominance, occurring approximately 1.5–2 times more frequently in women than in men, especially after puberty [120, 121]. Even among healthy individuals without chronic pain, women have demonstrated heightened sensitivity to pressure and thermal pain stimuli compared with men [122, 123]. Though detailed mechanisms underlying such gender disparities remain incompletely understood, gonadal hormones (particularly estrogen and testosterone) have been found with profound roles [113, 124]. Specifically, estradiol exhibits antinociceptive properties in healthy women at elevated concentrations [125, 126] and increases pain susceptibility at lower concentrations [127]. Testosterone mediates the antinociceptive effects and is protective against widespread pain. As demonstrated in a murine model, orchiectomized male mice developed prolonged and more widespread pain sensitivity compared with controls, while testosterone administration in either female or orchiectomized male animals reversed this hypersensitivity [128]. In the clinics, a 2013 study involving adult females using oral contraceptives reported that suppressed endogenous testosterone levels correlated with both a decreased threshold to thermal pain and reduced neuronal activity within the RVM, a key region of the descending pain modulatory system [127]. Further in support of this include the emergence of sex‐based differences in pain sensitivity around puberty [129], fluctuations in pain responses across phases of the menstrual cycle in women [130, 131], and the higher prevalence of adverse childhood experiences among females, a factor that may independently contribute to increased risk [132]. For example, the prevalence of most regional nociplastic pain conditions raised after puberty and persisted throughout the mid‐life [133]; a 2021 study has shown that in a 10‐year follow‐up period, dysmenorrhea increased the risk of developing chronic multisite pain (in nonpelvic anatomical locations), a cardinal feature of nociplastic pain [134]; another study has shown that women with IBS were more likely to report a history of adverse childhood experiences than men [132].

#### 4.1.3 Comorbidity‐Related Factors

Comorbidity‐related factors constitute a critical category of intrinsic and modifiable risk determinants that significantly drive the onset, severity, and persistence of chronic pain. These factors encompass a wide spectrum of systemic diseases, metabolic dysregulations, and immune disorders, which contribute to pain pathophysiology through direct neuropathic injury, systemic inflammation, biomechanical stress, and neuroimmune crosstalk, often creating complex, bidirectional relationships where pain and comorbidity exacerbate each other.

Chronic pain is a frequent and debilitating companion of major systemic diseases. In diabetes mellitus, hyperglycemia is a primary driver for the development of diabetic peripheral neuropathy, with approximately 50% of patients affected [135]. The correlation between disease severity and neuropathy risk is underscored by evidence that tight glycemic control can reduce the incidence of neuropathy by 69% over 6.5 years [136, 137], positioning hyperglycemia as a potent, modifiable metabolic factor for neuropathic pain. Similarly, in cancer, chronic pain is reported in more than 50% of patients [138]. The pain arises either directly from the disease, through invasion or compression of neural structures including spinal nerves, plexuses, and peripheral nerves, or from a consequence of oncological therapies, with one large study attributing pain to the cancer itself in 92.5% of severe cases and to treatment in 20.8% [138]. OA and chronic pancreatitis further exemplify how somatic and visceral pathologies, respectively, provide persistent nociceptive input that can initiate and sustain central sensitization, leading to chronic pain states that often outpace the progression of the primary disease.

Obesity represents a paramount comorbidity with a well‐established positive correlation to chronic pain across multiple types [139]. Epidemiologically, a high body mass index (BMI ≥ 30 kg/m^2^) is clearly linked to an increased incidence of neuropathic pain [89, 140, 141, 142] with weight independent of height and increased waist circumference also identified as significant risk factors [143, 144]. Beyond neuropathic pain, obesity predisposes individuals to nociplastic pain conditions like fibromyalgia [145]. The mechanisms are multifaceted and synergistic. Biomechanically, excess weight imposes increased load and stress on joints, muscles, and tendons, exacerbating conditions like OA and low back pain; studies show obese adults experience significantly higher lumbar disc compression forces [146]. Immunologically, adipose tissue is a prolific endocrine organ secreting proinflammatory adipokines (e.g., leptin, resistin) and cytokines (e.g., TNF‐α, IL‐6), creating a state of chronic low‐grade systemic inflammation that sensitizes peripheral and central pain pathways [147, 148]. Molecularly, obesity‐related pathways directly interact with pain biology. In rodent models, obesity‐induced neuropathic pain involves the AMPK/ERK/NOX4 pathway, where AMPK activation attenuates oxidative stress and pain [149]. Furthermore, the fat‐mass and obesity‐associated protein (FTO), an RNA demethylase expressed in injured dorsal root ganglia, plays a critical role in neuropathic pain maintenance in both male and female rodents, with FTO inhibition producing antinociceptive effects, highlighting a direct molecular link [150]. The clinical relevance of this comorbidity is underscored by interventions: weight loss, and even severe caloric restriction prior to significant mass reduction, can notably attenuate nociplastic and other chronic pain [151, 152].

A dysregulated or compromised immune system is a significant intrinsic risk factor for chronic pain, primarily by failing to contain threats that damage nerves or by perpetuating a state of pathological inflammation. This is most vividly illustrated in the context of PHN, a chronic neuropathic pain condition that develops in 9–24% of individuals following herpes zoster (shingles) infection [153]. The primary risk factor is immune compromise; the incidence of PHN increases progressively with age, paralleling the age‐related decline in immune function [154]. The risk is dramatically elevated in individuals with pronounced immunosuppression, such as people living with HIV (15 times higher herpes zoster incidence), patients with hematologic malignancies (e.g., leukemia, lymphoma), and recipients of bone marrow or solid organ transplants [155, 156]. Furthermore, iatrogenic immunosuppression from long‐term chemotherapy, radiotherapy, or corticosteroid use increases susceptibility [156]. The mechanism involves the reactivation of latent varicella‐zoster virus due to waning cell‐mediated immunity, which causes neuronal damage and inflammation, establishing a persistent neuropathic pain state. Beyond specific infections, a generally proinflammatory state, characterized by elevated circulating levels of cytokines like TNF‐α, IL‐1β, and IL‐6, can sensitize nociceptors, promote neuroinflammation via glial cell activation in the CNS, and drive central sensitization, thereby lowering pain thresholds and facilitating chronicity across various pain conditions.

The impact of comorbidity‐related factors is rarely isolated. Instead, they engage in pathophysiological crosstalk that amplifies pain. For example, obesity‐induced inflammation can exacerbate the neuroinflammatory component of diabetic neuropathy or OA. Similarly, the immune compromise from cancer or its treatment can unleash viral reactivations leading to neuropathic pain. This interconnectivity means that chronic pain in comorbid conditions is often multimechanistic, involving concurrent elements of nociceptive (e.g., joint damage in obesity), neuropathic (e.g., nerve compression in cancer, metabolic injury in diabetes), and nociplastic (e.g., central sensitization fueled by systemic inflammation) pain. Consequently, effective management necessitates a dual approach, that is, optimal management of the underlying comorbidity (e.g., glycemic control, weight loss, immune restoration) is fundamental to removing the primary driver, while concurrent, mechanism‐based pain therapy is required to address the established maladaptive plasticity within the nervous system.

### 4.2 Extrinsic Factors

#### 4.2.1 Environmental Factors

Environmental factors constitute a critical and diverse category of extrinsic determinants that significantly influence the initiation, maintenance, and chronicity of pain. These factors encompass direct physical insults to bodily tissues and nerves, as well as broader contextual exposures, which can act alone or synergistically with intrinsic vulnerabilities to shape an individual's pain trajectory.

Direct physical injury represents a quintessential environmental risk factor for chronic pain, most directly for the nociceptive type. This encompasses a wide array of traumatic events, including fractures, sprains, contusions, burns, and surgical incisions, which cause actual or potential damage to somatic or visceral tissues [157, 158]. The ensuing tissue disruption triggers a local inflammatory cascade, releasing a multitude of mediators (e.g., PGEs, cytokines, bradykinin) that sensitize peripheral nociceptors, leading to primary hyperalgesia and ongoing pain signaling. While the intensity of acute pain often correlates with the extent of tissue damage, the transition to chronicity is modulated by individual differences in genetic background, inflammatory reactivity, and psychological response, illustrating the interaction between environmental insult and host factors [157, 158].

A particularly consequential subcategory of environmental injury is iatrogenic nerve damage, most commonly resulting from surgical intervention. This serves as a principal extrinsic trigger for chronic neuropathic pain. Epidemiological data consistently indicate that persistent neuropathic pain develops in a substantial minority (10–50%) of patients following common procedures such as breast surgery [159], orthognathic surgery [160], thoracotomy [161], fracture surgery [162], lumbar stenosis surgery [163], and inguinal hernia repair [164]. The consistency of this outcome across disparate surgical contexts underscores direct neural trauma through transection, compression, entrapment, or inflammation as a key mechanistic driver. Beyond the immediate nerve injury, the surgical environment itself contributes through associated factors like the intensity of acute postoperative pain, which acts as a potent nociceptive barrage driving central sensitization, and potentially inadequate perioperative analgesia [4]. This highlights that the environmental factor is not a single event but a perioperative continuum of risk.

Beyond discrete injuries, environmental factors include sustained physical exposures and systemic challenges. Occupational and repetitive strain injuries from ergonomic stressors (e.g., prolonged poor posture, repetitive motions) are environmental contributors to chronic musculoskeletal pain conditions. Furthermore, systemic biological insults, such as severe infections, can act as environmental triggers for nociplastic pain syndromes, as evidenced by postviral conditions like post‐COVID syndrome, where the pathogen exposure initiates a cascade of neuroimmune dysregulation leading to widespread pain and fatigue. Socioeconomic and physical environments also play a profound role; factors such as chronic stress from unsafe neighborhoods, occupational instability, or lack of access to green spaces can perpetuate stress‐system dysregulation (e.g., HPA axis dysfunction), promote systemic inflammation, and diminish an individual's capacity for recovery, thereby facilitating the transition and maintenance of chronic pain states.

In synthesis, environmental factors in pain are multifactorial, ranging from discrete physical and iatrogenic injuries that directly damage tissues and nerves, to broader contextual exposures that alter systemic physiology and neural processing. The path from an environmental insult to chronic pain is not deterministic but is mediated by the intensity and nature of the exposure, its interaction with genetic and psychological susceptibilities, and the subsequent neuroplastic changes it induces in both peripheral and CNSs. Effective pain prevention and management, therefore, require not only treating the biological consequences of these environmental factors but also, where possible, mitigating their occurrence through improved surgical techniques, ergonomic interventions, and public health policies aimed at reducing broader environmental stressors.

#### 4.2.2 Lifestyle Factors

Physical inactivity is a well‐established extrinsic risk factor contributing to the development and maintenance of chronic pain, particularly of the nociplastic type. Evidence from both preclinical and clinical studies indicates that sedentary behavior disrupted endogenous pain modulatory systems, thereby facilitating central sensitization and widespread pain. One proposed mechanism involves the impairment of descending inhibitory pathways. It has been demonstrated from animal models that regular physical activity reduced nociceptive sensitivity through activation of the RVM, a key brainstem region involved in pain control [165]. Clinically, higher levels of physical activity are associated with reduced pain facilitation, whereas sedentary behavior was correlated with impaired conditioned pain modulation, a psychophysical indicator of descending inhibition [166]. Furthermore, the cessation of habitual exercise in previously active individuals induced nociplastic‐like symptoms, including generalized pain and fatigue [167]. Supportive evidence are also available from large‐scale human studies. For instance, an inverse relationship between objectively measured physical activity levels and the number of painful body sites, a key feature of nociplastic pain, was revealed from a cross‐sectional analysis of UK Biobank data [168]. This suggests that physical inactivity may not only be a consequence but also an extrinsic contributor to chronic pain, likely through mechanisms that involve reduced endogenous analgesia, increased proinflammatory signaling, and enhanced central sensitization. These insights have highlighted the importance of promoting physical activity as a nonpharmacological strategy to mitigate the risk and burden of chronic nociplastic pain.

Sleep disturbance is another well‐established extrinsic risk factor contributing to the development and exacerbation of chronic pain, especially nociplastic pain [169]. Numerous studies have demonstrated that sleep deprivation could induce fibromyalgia‐like symptoms such as widespread pain, fatigue, and cognitive dysfunction in otherwise healthy individuals [170, 171, 172]. However, it remains unclear which specific aspects of sleep such as total duration, sleep architecture, or circadian rhythm are most critically involved. These findings have suggested that disrupted sleep might actively drive maladaptive changes in central pain processing rather than merely accompany chronic pain conditions. Although the association is clear, the specific mechanisms remain to be incompletely understood. It has not yet been established whether total sleep duration, alterations in sleep architecture (such as reduced slow‐wave or REM sleep), or disruptions in circadian rhythmicity play the most critical role in facilitating pain sensitization. Nonetheless, sleep disturbance appears to impair descending inhibitory pathways, promote neuroinflammation, and alter neurotransmitter systems involved in pain modulation, all of which are hallmarks of nociplastic pain pathophysiology [169]. These facts have positioned sleep disturbance as a modifiable extrinsic factor that could be targeted for both the prevention and management of chronic nociplastic pain. Further studies are needed to elucidate the specific sleep‐related mechanisms involved and to establish chronobiological and behavioral interventions that can mitigate their impact on pain perception.

#### 4.2.3 Psychosocial Factors

Psychosocial factors represent a critical and dynamic domain of extrinsic risk that profoundly influences the development, perception, maintenance, and severity of chronic pain, encompassing neuropathic, nociceptive, and particularly nociplastic pain conditions. These factors, which include prior pain history and stressor exposure across the lifespan, as well as psychological traits and states, do not merely modulate pain but actively interact with biological substrates to drive maladaptive neuroplasticity, thereby transitioning acute pain into a persistent, complex chronic state. The theoretical framework for understanding chronic pain has consequently evolved from a purely biomedical model to a biopsychosocial one, recognizing pain as the emergent product of dynamic interactions between pathophysiological mechanisms and psychosocial variables [173, 174].

A significant history of prior pain serves as a potent psychosocial and neurobiological primer for chronicity. Clinical evidence robustly demonstrates that the intensity and duration of previous nociceptive experiences can predispose individuals to persistent pain states through maladaptive neuroplastic alterations. For instance, patients who endure severe acute pain during herpes zoster infections are significantly more likely to develop PHN, suggesting that robust initial nociceptive input can drive long‐term central sensitization and sensory dysfunction [175, 176, 177]. Similarly, amputees with a history of intense and prolonged preamputation pain in the affected limb exhibit a higher incidence of severe phantom limb pain [178, 179]. This pattern highlights how prior pain exposure acts as an extrinsic trigger, promoting pain chronification via mechanisms such as entrenched central sensitization, altered synaptic efficacy, and structural reorganization within somatosensory pathways, making comprehensive pain history assessment essential for identifying at‐risk individuals.

Stressor exposure, both in early life and adulthood, operates as a fundamental extrinsic driver of chronic pain, with particularly strong links to nociplastic pain syndromes. Early‐life adversity during critical developmental periods—including intrauterine stress, maternal stress, low birth weight [180], neonatal intensive care admission [181], and childhood trauma—is consistently associated with heightened pain sensitivity [182] and a markedly increased risk of developing chronic overlapping pain conditions in adulthood [183, 184, 185]. The Adverse Childhood Experiences study established a clear link between childhood trauma and later‐life health morbidity [186], with chronic pain conditions such as fibromyalgia [183], chronic pelvic pain [184], and functional abdominal pain [185] being frequently observed. Population‐based studies suggest these experiences are significant contributory risk factors, accounting for a portion of cases, such as approximately 10% of fibromyalgia cases [187]. In adulthood, exposure to major physical or psychological stressors, such as motor vehicle accidents, terrorist attacks, or severe infections, can initiate or amplify nociplastic pain. A prominent contemporary example is post‐COVID syndrome, manifesting as widespread pain, fatigue, and cognitive dysfunction following SARS‐CoV‐2 infection [188]. While certain features may be pathogen specific, the core clinical presentation aligns with established postinfectious nociplastic syndromes, underscoring the role of significant stressors in triggering maladaptive central pain processing through mechanisms like dysregulated stress‐response systems (e.g., HPA axis) and long‐term alterations in neural function [189].

Concurrent psychological factors are integral to pain perception and persistence. The contributions of pain‐related fear, anxiety, depression, catastrophizing, and negative pain memories are well established [190]. In healthy adults, low mood amplifies pain perception through maladaptive cognitive processes and heightened activity in pain‐related brain regions [191]. A 2023 longitudinal study using UK Biobank data emphasized that psychosocial factors, including persistent low mood and exposure to stressful life events, significantly contributed to the development of widespread pain over a 9‐year period [192]. These factors directly shape clinical outcomes; for example, in lower‐limb amputees, psychosocial measures like catastrophizing and perceived social support recorded 1 month postamputation predict phantom limb pain severity up to 2 years later [193]. Similarly, preoperative anxiety correlates with heightened postoperative pain, and fear‐driven avoidance behaviors negatively shape recovery trajectories [194]. Conversely, psychological flexibility, the ability to adapt to pain‐related thoughts and feelings while pursuing valued goals, has been proposed as a key resilience factor among individuals with chronic pain [194, 195]. This highlights a spectrum of psychological influence, where maladaptive processes exacerbate pain chronicity, while adaptive processes can foster resilience.

In synthesis, psychosocial factors are not peripheral contributors but are central to the etiology and trajectory of chronic pain. Prior pain history primes the nervous system for sensitization, life stressors disrupt neuroendocrine and neural homeostasis to trigger or exacerbate pain states, and psychological factors continuously modulate the perceptual and emotional experience of pain. These elements interact synergistically, creating a self‐perpetuating cycle where, for instance, a history of childhood adversity (stressor) may increase vulnerability to catastrophizing (psychological factor) following a new injury (prior pain event), thereby dramatically elevating the risk of transition to a chronic, nociplastic pain state. This integrated biopsychosocial model necessitates that comprehensive assessment and effective intervention for chronic pain must explicitly address these intertwined psychosocial dimensions alongside biological pathology.

In summary, chronic pain emerges from a complex, dynamic interplay of intrinsic and extrinsic risk factors that operate across the biopsychosocial spectrum. Intrinsic vulnerabilities, such as genetic polymorphisms, female gender, and comorbid conditions (e.g., diabetes, obesity, immune compromise), create a predisposed biological substrate. Extrinsic factors, including physical injuries, surgical trauma, lifestyle elements (physical inactivity, sleep disturbance), and profound psychosocial exposures (prior pain history, life stressors, maladaptive psychological traits), act as critical triggers and perpetuators (Figure 3). These domains do not act in isolation; instead, they engage in synergistic interactions. That is, a genetic predisposition, an environmental injury (e.g., surgical trauma), and psychological distress can converge and synergistically drive maladaptive neuroplasticity and disrupt homeostatic systems, thereby facilitating the transition from acute nociception to a persistent chronic pain state. This comprehensive risk factor landscape underscores the necessity of a holistic, personalized approach to prevention, assessment, and management that simultaneously targets biological, psychological, and social determinants to effectively mitigate the burden of chronic pain.

## 5 Diagnostic Frameworks and Emerging Biomarkers

**FIGURE 4:**
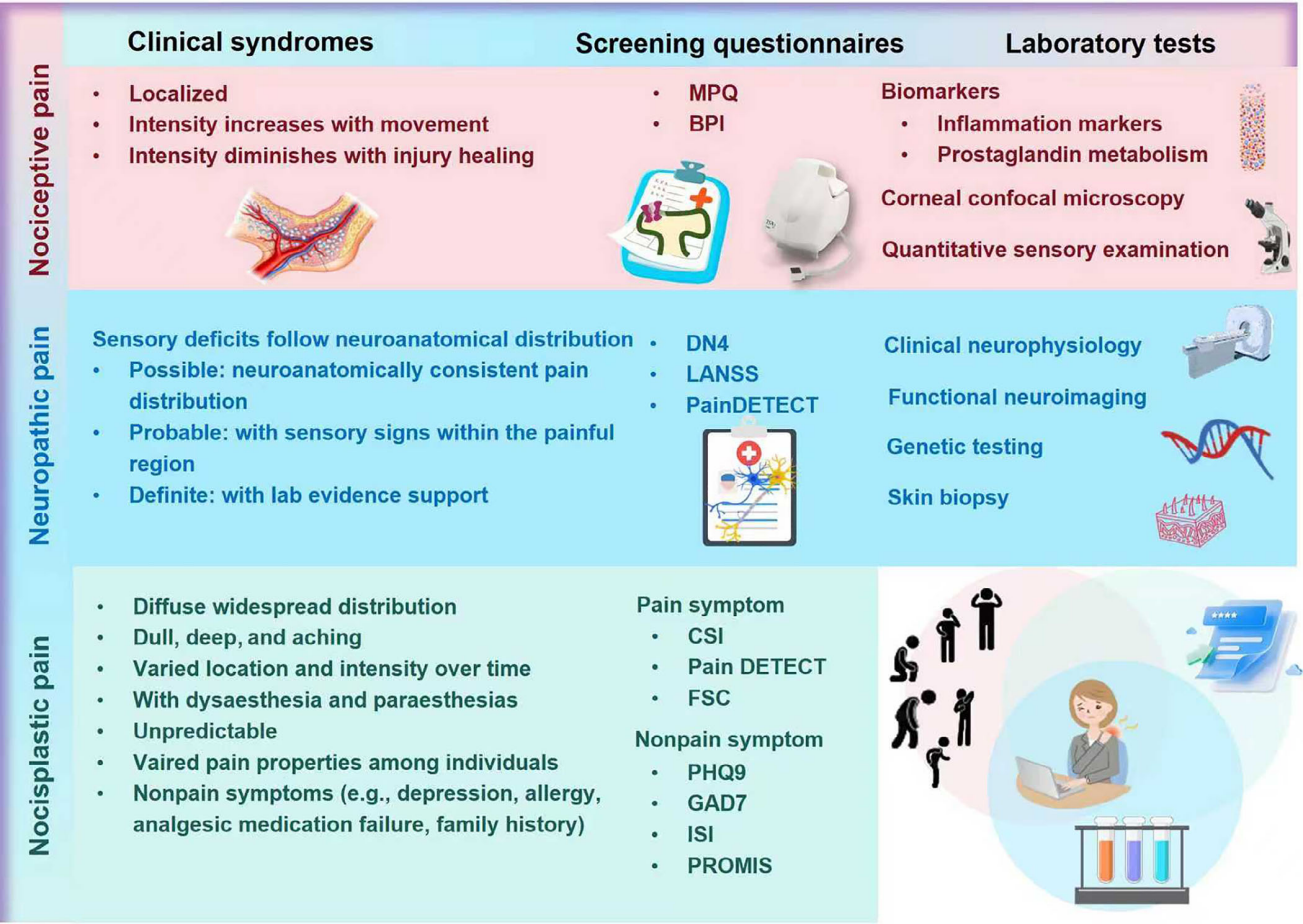
Primary diagnosis modalities for each type of chronic pain. Diagnosis of chronic pain is an integrated process of clinical syndromes, screening questionnaires, and laboratory tests to identify mixed pain and guide personalized treatment. Nociceptive pain is localized, with the pain intensity directly associated with the injury and movement; the sensory deficits of neuropathic pain follow neuroanatomical distribution; and nocisplastic pain is featured with diffuse widespread distribution, unpredictable and with nonpain syndromes such as depression, allergy, and even familial history. In addition to these clinical syndromes, nociceptive pain can be assessed using tools like MPQ and BPI, along with biomarkers, corneal confocal microscopy, and quantitative sensory testing. Neuropathic pain can be evaluated with DN4, LANSS, or painDETECT questionnaires, supported by clinical neurophysiology, functional neuroimaging, skin biopsy, and genetic testing to confirm nerve involvement. Nociplastic pain is challenging to diagnose solely via screening questionnaires and laboratory tests. However, it frequently coexists with nociceptive and neuropathic pain. Therefore, instruments primarily used for diagnosing the latter can also provide useful supportive information for identifying nociplastic components.

The accurate diagnosis of chronic pain, a prerequisite for effective management, hinges on distinguishing its underlying pathophysiological mechanisms, that is, nociceptive, neuropathic, and nociplastic. This section systematically delineates the clinical syndromes, screening questionnaires, and laboratory investigations that form the contemporary diagnostic armamentarium for each pain type (Table 1 and Figure 4). For nociceptive pain, characterized by well‐localized symptoms linked to tissue injury, we examine tools like the McGill Pain Questionnaire (MPQ) and discuss biomarkers of inflammation. For neuropathic pain, resulting from somatosensory system damage, we detail the use of validated screening tools such as Douleur Neuropathique 4 (DN4), confirmatory neurophysiological tests such as laser‐evoked potentials (LEPs) and skin biopsy. Finally, for the complex entity of nociplastic pain, defined by CNS sensitization in the absence of clear neural or tissue damage, we review the characteristic clinical phenotype, supportive questionnaires such as Central Sensitization Inventory (CSI), and the challenges in identifying objective biomarkers. This structured, mechanism‐based diagnostic framework is essential for moving beyond symptomatic treatment toward personalized, pathophysiology‐targeted therapeutic strategies.

**TABLE 1:** Major instruments for chronic pain diagnosis.

Assessment tool	Purpose	Pain subtype	Key items	Sensitivity/specificity	Typical clinical setting	References
MPQ	To differentiate pain dominated by nociceptive mechanisms from neuropathic pain	Nociceptive pain; applicable to mixed pain	78 descriptors (sensory, affective, evaluative categories), PRI, VAS	NA	Detailed profiling in research and nonacute patient investigations	[196, 197]
Short‐form MPQ (SF‐MPQ)	To provide a quicker multidimensional pain assessment while retaining the core structure of the MPQ	Nociceptive pain; applicable to mixed pain	22 descriptors categorized into four subscales: continuous pain, intermittent pain, neuropathic pain, affective pain; uses a 0–10 Numerical Rating Scale	Sensitivity 93%, specificity 82%	Routine rapid clinical assessment for monitoring changes in pain quality and intensity	[198, 199, 200, 201]
SF‐MPQ‐2	An enhanced version of SF‐MPQ with improved ability to identify neuropathic pain components	Nociceptive pain; applicable to mixed pain	22 descriptors categorized into four subscales: continuous pain, intermittent pain, neuropathic pain, affective pain; uses a 0–10 Numerical Rating Scale (NRS)	Sensitivity 89.5%, specificity 75.0%	Routine rapid clinical assessment	[202, 203]
BPI	Pain severity and impact on daily function	Nociceptive pain	Two core dimensions: pain severity (4 items) and pain interference (7 items)	Sensitivity 79.37%, specificity 46.9%	Cancer pain management, chronic pain assessment, and quality of life monitoring	[204]
DN4	To assess suspected neuropathic pain	Neuropathic pain	7 symptom descriptors (e.g., burning, electric shocks) and 3 clinical examination items (tactile allodynia, pinprick hypoesthesia)	Sensitivity 89%, specificity 88%	First‐line screening tool when history suggests possible neuropathic pain	[205, 206]
LANSS	Rapid identification of neuropathic pain based on symptoms and simple bedside sensory tests	Neuropathic pain	5‐item symptom questionnaire and 2 bedside sensory tests (allodynia, altered pinprick threshold)	Sensitivity 80%, specificity 87%	Rapid bedside assessment in emergency departments, wards, or time‐limited outpatient settings	[205, 207]
PainDETECT	A self‐report screening tool, requiring no physical examination, for identifying neuropathic pain components	Neuropathic pain	9 descriptors of pain quality (e.g., burning, tingling), radiating pattern, and temporal characteristics	Sensitivity 84%, specificity 80%	Initial screening for neuropathic pain, patient self‐assessment	[206]
CSI	To assess a spectrum of somatic and affective symptoms associated with central sensitization	Nociplastic pain	25 items covering a wide range of somatic symptoms (pain, headache, irritable bowel) and affective/cognitive symptoms (fatigue, mood issues)	Sensitivity 81%, specificity 75%	Identifying neurophysiological and symptomatic features commonly associated with nociplastic pain	[208, 209]
FSC	To quantify the severity of fibromyalgia symptoms	Nociplastic pain	WPI (0–19) and SSS (0–12)	Sensitivity 88.5%, specificity 81.5%	Diagnosis, severity stratification, long‐term monitoring, and clinical research of fibromyalgia	[210, 211]
PHQ9	To screen for and assess the severity of depressive symptoms	Comorbid depressive symptoms in nociplastic pain	9 items corresponding to DSM‐IV/5 diagnostic criteria for depression, assessing frequency over the past 2 weeks	Sensitivity 85%, specificity 85%	Routine screening for psychological comorbidities in chronic pain management, monitoring impact of depression on pain and treatment	[212, 213]
GAD7	To screen for and assess the severity of generalized anxiety symptoms	Comorbid anxiety symptoms in nociplastic pain	7 items assessing the frequency of core anxiety symptoms over the past 2 weeks	Sensitivity 64%, specificity 91%	Routine screening for psychological comorbidities in chronic pain management, assessing anxiety status	[214, 215]
ISI	To assess the severity of insomnia and its impact on daytime function	Comorbid sleep disturbance in nociplastic pain	7 items assessing difficulty falling asleep, sleep maintenance, early awakening, sleep satisfaction, and daytime distress	Sensitivity 86%, specificity 87%	Systematic evaluation of insomnia, a common comorbidity in chronic pain, guiding sleep hygiene interventions	[216]
PROMIS	To provide standardized, efficient measurement of patient‐reported outcomes across physical, mental, and social health domains	Multidimensional symptom of nociplastic pain; applicable to all chronic pain	A modular system containing a large item bank; enables creation of independent short forms for specific domains (pain interference, fatigue) or dynamic administration via CAT	Sensitivity 81%, specificity 84%	Clinical research and high‐quality practice: systematic assessment of pain's broad impact on life; tracking improvements in overall health status from interventions; enabling standardized data comparison across studies and institutions	[217, 218]

*Abbreviations*: BPI, Brief Pain Inventory; CAT, computerized adaptive testing; CSI, Central Sensitization Inventory; DN4, Douleur Neuropathique 4; FSC, Fibromyalgia Survey Criteria; GAD7, Generalized Anxiety Disorder‐7; ISI, Insomnia Severity Index; LANSS, Leeds Assessment of Neuropathic Symptoms and Signs; MPQ, McGill Pain Questionnaire; NA, not available; NRS, Numerical Rating Scale; PHQ9, Patient Health Questionnaire‐9; PRI, present pain intensity; PROMIS, Patient‐Reported Outcomes Measurement Information System; SF‐MPQ, Short‐Form MPQ; SF‐MPQ‐2, Short‐Form McGill Pain Questionnaire‐2; SSS, Symptom Severity Score; VAS, Visual Analog Scale; WPI, Widespread Pain Index.

### 5.1 Nociceptive Pain

#### 5.1.1 Clinical Syndromes

Clinical manifestations of nociceptive pain vary according to the origin and severity of tissue injury, and commonly include aching, throbbing, burning, stabbing, cramping, gnawing, or pressure‐like sensations [55]. Accompanying signs may include localized numbness, weakness, tingling, erythema, warmth, or swelling [219]. This type of pain is typically well localized, with the intensity often exacerbating with movement or mechanical stress and tending to diminish with the healing of the affected tissues.

#### 5.1.2 Screening Questionnaires

Screening questionnaires provide valuable support in the clinical identification and characterization of nociceptive pain, especially in cases where a mixed nociceptive–neuropathic presentation is suspected. MPQ and the Brief Pain Inventory (BPI) have been widely used to aid in distinguishing predominantly nociceptive from neuropathic mechanisms by systematically assessing pain qualities, spatial distribution, temporal characteristics, and intensity. While these tools cannot replace comprehensive clinical evaluations involving imaging or diagnostic blocks, they may offer a standardized method to screen for nociceptive pain, monitor its progression, and evaluate nociceptive contributions within the mixed‐pain conditions.

The original MPQ was the first tool to enable multidimensional pain assessment, capturing sensory and affective dimensions, intensity, and subjective impact [197]. It was later succeeded by the Short‐Form MPQ (SF‐MPQ) [201] and the SF‐MPQ‐2 [203, 220], which were both designed to enhance the usability, sensitivity to change, and clinical applicability through improved structure and scaling. For example, the original MPQ's scoring system could easily confound pain quality and quantity, as high scores could result from either numerous mild descriptors or a few severe ones. To address this, the SF‐MPQ and SF‐MPQ‐2 reduced the number of items and improving the numeric scaling from a 4‐point scale to an 11‐point scale in the SF‐MPQ‐2 [221]. Furthermore, these revised versions refined the selection of sensory descriptors. Specifically, the SF‐MPQ‐2 introduced three distinct sensory subscales while retaining the affective scale and implemented a structured response format [221]. As a result, these short revisions have demonstrated satisfactorily high reliability, improved discriminative validity across pain conditions, and greater responsiveness in tracking the treatment outcomes as compared with the original MPQ [222].

The BPI, originally developed for assessing cancer pain, uses numeric rating scales to evaluate both pain severity and its impact on daily function and helps identify patterns suggestive of somatic or visceral nociception such as localized aching or pressure‐sensitive pain exacerbated by the movement. The instrument was initially based on a two‐factor model encompassing “pain intensity” and “pain interference with daily functioning,” using the patient's experience over the preceding week as the reference period.

Subsequent refinements have expanded its conceptual framework, including a three‐factor model distinguishing “pain intensity,” “interference with activities,” and “interference with affectivity” [223, 224], as well as a second‐order “pain interference” factor encompassing both activity‐ and emotion‐related domains [225]. The BPI is available in two formats, that is, a long form with 32 items and a short form with 15 items [226]. Both versions retain identical core items aligned with the factorial models, which differ primarily in the number of supplementary items and the reference period (i.e., past week for the long form, past 24 h for the short form) [226]. Although the long form allows more comprehensive characterization of the pain experience, the short form has been more widely used in both academic and clinical practice due to its brevity and practicality [226, 227].

#### 5.1.3 Laboratory Tests

Beyond capturing subjective pain experiences through descriptor endorsement in the form of screening questionnaires, an implicit objective of pain diagnosis is to suggest the underlying pain mechanisms and, in particular, differentiate nociceptive from neuropathic pain. Various targeted laboratory tests have been established for this purpose, including, for example, biomarkers, corneal confocal microscopy (CCM), and quantitative sensory examination. Clinical neurophysiology, functional neuroimaging, skin biopsy and genetic testing may also be employed to help differentiate nociceptive from neuropathic pain, which will be discussed in detail in the section *Neuropathic Pain*.

##### 5.1.3.1 Biomarkers

The well‐established link between inflammation and pain, wherein proinflammatory cytokines induce and facilitate pain by acting on peripheral nociceptors, has long positioned chronic pain as a clinical sign of inflammation [228]. Although cytokines and chemokines measured in experimental inflammatory models have shown a great promise as biomarkers, their clinical utility has been limited by inconsistent results across studies, likely due to variations in sample types (e.g., serum, skin biopsies) and patient heterogeneity [229]. While no standalone biomarker specifically diagnoses nociceptive pain, commonly measured indicators of tissue damage, inflammation, or systemic conditions contributing to nociception include C‐reactive protein (CRP), erythrocyte sedimentation rate, proinflammatory cytokines (e.g., IL6, TNF‐α), and creatine kinase. Other promising candidates include the products of the PGE metabolic pathway, which may reflect the activity of inflammatory pain [230]. For example, serum lipids serving as substrates for PGE synthesis have been correlated with pain intensity, though these are often confounded by comorbidities and other factors [231]. Despite the theoretical appeal of objective pain biomarkers [232, 233, 234], it is unlikely that any single mediator will specifically indicate pain. A more viable approach may involve developing integrated mediator profiles within a systems‐based framework [235, 236]. Furthermore, biomarker results should always be interpreted in conjunction with clinical evaluation, symptom‐based questionnaires, and other laboratory findings to support the identification of nociceptive mechanisms, monitor disease progression, and assess responses to anti‐inflammatory or disease‐modifying treatments.

##### 5.1.3.2 Corneal Confocal Microscopy

CCM is a noninvasive in vivo imaging technique that permits quantitative assessment of corneal nerve fibers, consisting exclusively of thinly myelinated Aδ‐fibers and small unmyelinated C‐fibers, and is used to evaluate small‐fiber damages in systemic peripheral neuropathies. By analyzing parameters such as corneal nerve fiber length, density, and branching density, CCM has demonstrated good correlations with peripheral neuropathy severity in conditions such as diabetic neuropathy and sarcoidosis, as confirmed by neurophysiological tests and skin biopsy. According to a large study involving 998 diabetic patients, these parameters have demonstrated moderate sensitivity (52–67%) and specificity (60–68%) in detecting peripheral neuropathy, regardless of the presence of neuropathic pain [237]. However, evidence regarding the diagnostic accuracy of CCM specifically for painful small fiber neuropathy remains limited and inconsistent. One study involving 16 patients reported the sensitivity and specificity values of CCM being 44 and 55%, respectively, as compared with skin biopsy [238], whereas another found that the sensitivity and specificity of these three main CCM parameters ranged between 60 and 80% [239]. Although CCM has demonstrated a great promise as an objective tool for the assessment of small‐fiber integrity, its diagnostic utility has not yet been firmly established and cannot be broadly generalized in the routine clinical practice.

##### 5.1.3.3 Quantitative Sensory Examination

Quantitative sensory testing (QST) is a psychophysiological diagnostic method that uses standardized mechanical and thermal stimuli to quantitatively evaluate both nociceptive and non‐nociceptive sensory pathways. By administering stimuli of graded intensities and recording patient responses, via button press or verbal report, regarding detection and pain thresholds, QST can detect both negative (e.g., hypoesthesia, hypoalgesia) and positive (e.g., allodynia, hyperalgesia, hyperpathia) sensory abnormalities across multiple modalities [240]. This approach enables the objective quantification of both sensory loss and gain of function.

A widely adopted standardized protocol, comprising 13 parameters that assess various dimensions of sensory processing, has been frequently used to characterize detailed sensory profiles in patients with chronic pain and to subgroup them according to the mechanisms of action [241]. In the clinical practice, QST offers functional insights into small‐fiber integrity, making it particularly useful in conditions involving selective damages to aδ‐ and c‐fibers, where conventional neurophysiological techniques such as nerve conduction studies (NCSs) and somatosensory evoked potentials often yield normal results [242]. Although comprehensive data on its diagnostic sensitivity and specificity for chronic pain remain limited, QST has evolved as an established tool for the functional assessment of disorders affecting the somatosensory system [206]

### 5.2 Neuropathic Pain

#### 5.2.1 Clinical Syndromes

Neuropathic pain, resulting from a lesion or disease of the somatosensory nervous system, is characterized by sensory deficits and requires a comprehensive somatosensory examination to identify relevant signs and symptoms. Sensory deficits in neuropathic pain typically follow a neuroanatomically plausible distribution corresponding to peripheral nervous system or CNS territories. In contrast, pain, particularly evoked pain, may extend beyond these anatomical boundaries, as often observed in painful mononeuropathies where dynamic mechanical allodynia spreads beyond the anatomical territory of the inflicted nerve [243]. A grading system recommended by IASP provides a structured diagnostic framework in the diagnosis of neuropathic pain [244]. Specifically, neuropathic pain is classified as “possible” when the pain distribution is neuroanatomically consistent, considered “probable” if the pain is accompanied with confirmatory sensory signs within the painful region, and deemed “definite” when the evidence can be objectively supported by rigorous laboratory examinations.

#### 5.2.2 Screening Questionnaires

Screening questionnaires are valuable tools in the clinical assessment of suspected neuropathic pain, especially in patients with localized rather than widespread symptoms [245, 246, 247]. These tools assess characteristic neuropathic pain descriptors, including burning, tingling, dynamic mechanical allodynia, electric shock‐like sensations, cold‐evoked pain, and numbness, with a minimum combination of such features indicating a possible neuropathic etiology. Widely used instruments include the DN4, the Leeds Assessment of Neuropathic Symptoms and Signs (LANSS), along with their self‐report versions (I‐DN4 and S‐LANSS), and PainDETECT [205]. While DN4 and LANSS incorporate both symptom descriptors and a brief physical examination requiring clinician administration, PainDETECT and the self‐report variants rely exclusively on patient‐reported verbal descriptors. DN4 and LANSS employ dichotomous cut‐off scores to classify probable neuropathic pain, whereas PainDETECT utilizes a graded scoring system, With scores ≥19 indicating probable neuropathic pain and scores between 12–18 suggesting ambiguous or mixed mechanisms [205]. Among these, the DN4 has been widely regarded as the most diagnostically accurate instrument, as evidenced by a meta‐analysis of 27 studies involving 4774 patients, which reported a sensitivity of 0.89 (95% CI: 0.86–0.92) and a specificity of 0.88 (95% CI: 0.83–0.92) [206].

#### 5.2.3 Laboratory Tests

##### 5.2.3.1 Clinical Neurophysiology

Clinical neurophysiology is integral to the assessment of neuropathic pain, as it enables the objective evaluation of functional disturbances in the somatosensory nervous system and has become a standard component of clinical care. Established methods including NCSs and somatosensory‐evoked potentials have continued to serve as cornerstone techniques for identifying and measuring deficits in large myelinated sensory fibers within both peripheral and central pathways [248]. Although these approaches do not directly examine nociceptive transmission, they yield valuable information about the structural and functional impairments that are commonly associated with neuropathic pain. For evaluating small‐fiber dysfunction, a frequent contributor to neuropathic pain, microneurography allows direct recording of unmyelinated C‐fiber activity and has been instrumental in detecting aberrant nociceptor responses in painful neuropathies [249]. Nonetheless, its clinical application is limited due to technical challenges, a high degree of operator skill requirement, and a lack of universally accepted diagnostic norms. As an alternative, the Quantitative Sudomotor Axon Reflex Test serves as an indirect measure of small‐fiber function by assessing sudomotor activity, which is mediated by autonomic C‐fibers often compromised in neuropathic disorders [250]. The RIII flexion reflex and the corneal reflex have been primarily used as neurophysiological tools to study the modulation of nociceptive pathways under various physiological and pharmacological conditions [251]. Additionally, LEPs and contact heat‐evoked potentials, which activate nociceptors through radiant and conductive heat, respectively, have been increasingly utilized in the diagnosis of neuropathic pain, especially in cases of small fiber neuropathy. These techniques have demonstrated diagnostic sensitivities ranging from 0.66 to 0.79 and specificities between 0.82 and 0.90, using skin biopsy as a reference standard [206, 252, 253, 254]. Despite the expanding repertoire of neurophysiological assessments, integrating ancillary approaches such as QST may enhance the multidimensional characterization of mechanisms driving neuropathic pain.

##### 5.2.3.2 Functional Neuroimaging

Functional neuroimaging techniques, notably positron emission tomography and functional magnetic resonance imaging (MRI), have significantly advanced our understanding of the central mechanisms underlying neuropathic pain, including both spontaneous and provoked forms, although their use remains limited to preclinical studies rather than clinical diagnosis [255]. It has been shown that spontaneous neuropathic pain is linked to reduced thalamic activity contralateral to the side of pain, manifested as decreased glucose metabolism and cerebral blood flow [256]. This thalamic hypoactivity is further corroborated by structural and metabolic evidence, such as gray matter volume loss observed in voxel‐based morphometry and reduced neuron‐specific metabolite peaks in magnetic resonance spectroscopy. Importantly, these thalamic abnormalities appear to be unique to neuropathic pain and may be reversible with successful analgesic treatment. In contrast, provoked neuropathic pain, such as dynamic mechanical allodynia, involves both quantitative and qualitative functional reorganization. Quantitatively, non‐noxious stimuli evoke brain responses similar in intensity to those triggered by normally painful stimuli. Qualitatively, changes include a transition from thalamic hypoactivity to hyperactivity, a shift of activation from ventrolateral to medial thalamic nuclei, paradoxical hyperactivation of the operculo–insular cortex, and reduced responsiveness in the ventromedial PFC [206, 255]. Collectively, these findings have underscored the importance of central neural reorganization in neuropathic pain and deepened our pathophysiological insights into both spontaneous and evoked symptoms of chronic pain.

##### 5.2.3.3 Skin Biopsy

Skin biopsy is a widely employed diagnostic tool for staining and quantifying cutaneous nerve fibers in peripheral neuropathies [257], offering a valuable alternative for confirming small fiber neuropathy when other diagnostic modalities yield inconclusive results. The procedure involves sterile collection under local anesthesia using a disposable punch, typically 3 mm in diameter, from distal (e.g., leg) and proximal (e.g., thigh) sites to evaluate length‐dependent pathology, without the need for sutures. Thin sections (typically of 50 µm) are immunostained to visualize intraepidermal nerve fibers, predominantly unmyelinated C‐fiber terminals and some small myelinated Aδ‐fibers, as well as sweat glands, blood vessels, and cellular infiltrates, with both bright‐field immunohistochemistry and immunofluorescence exhibiting comparable diagnostic accuracy [258]. While intraepidermal nerve fiber density does not consistently distinguish between neuropathic and nociceptive pain, it is correlated with specific pain phenotypes [259]. For example, partially preserved fiber density has been linked to dynamic mechanical allodynia [260]. Furthermore, immunostaining patterns of peptidergic and regenerating fibers have shown potential as pathological biomarkers in conditions such as painful diabetic neuropathy [261, 262]. A recent meta‐analysis of six studies reported pooled sensitivity and specificity of 0.84 (95% CI: 0.75–0.90) and 0.86 (95% CI: 0.70–0.94), respectively [206], supporting the utility of skin biopsy in diagnosing painful small fiber neuropathy.

##### 5.2.3.4 Genetic Testing

Genetic testing, while not yet integrated into routine clinical assessment for neuropathic pain, plays a definitive diagnostic role in specific monogenic hereditary disorders such as erythromelalgia and paroxysmal extreme pain disorder. The most commonly implicated gene is *SCN9A*, which encodes the voltage‐gated sodium channel Nav1.7. Biallelic loss‐of‐function mutations in *SCN9A* resulted in congenital insensitivity to pain, whereas more than 20 distinct rare gain‐of‐function variants have been associated with inherited erythromelalgia and another group of gain‐of‐function variants have been linked to paroxysmal extreme pain disorder [263]. Additionally, several rare pathogenic variants in *SCN9A* have been identified in patients carrying small fiber neuropathy [92], further underscoring the critical involvement of this gene in potentiating the mechanism of neuropathic pain.

### 5.3 Nociplastic Pain

#### 5.3.1 Clinical Syndromes

Individuals with nociplastic pain typically report a persistent, diffuse discomfort that is widespread throughout the body and disproportionate to any identifiable peripheral pathology [46, 209]. Nociplastic pain is often poorly localized, frequently affecting regions such as the upper arms or legs, and is commonly described as deep, dull, and aching in quality [264]. Its location and intensity tend to fluctuate over time and may be accompanied by dysesthesias or paresthesias [46, 264]. Nociplastic pain is also notably variable and unpredictable, often exacerbated or alleviated by nonspecific and multifactorial triggers [46].

Beyond pain, a constellation of nonpain symptoms supports the diagnosis of nociplastic pain. These include intrusive fatigue, nonrestorative sleep, depressed mood, deficits in concentration and short‐term memory, and heightened sensitivity to visual, auditory, or tactile stimuli [46, 209, 265]. Additional supportive features include the presence of multiple comorbid conditions or sensitivities, sometimes labeled as “allergies,” as well as a documented lack of response to conventional analgesics or peripheral interventions [46, 75, 218, 264]. A personal or family history of chronic pain that is not localized to a specific anatomical site may further suggest a nociplastic etiology. Finally, a pervasive sense of being overwhelmed by the cumulative burden of these multisystem symptoms can serve as a valuable clinical indicator for nociplastic pain [12, 75].

#### 5.3.2 Screening Questionnaires

A range of standardized questionnaires are available to complement, though not replace, comprehensive clinical assessment in the diagnosis of nociplastic pain. While screening tools such as the CSI can help identify neurophysiological and symptomatic features commonly associated with nociplastic pain [208, 209], abnormal scores on neuropathic pain instruments such as PainDETECT can aid in distinguishing between neuropathic and nociplastic pain mechanisms [209, 211, 266, 267]. Furthermore, the Fibromyalgia Survey Criteria provide a continuous measure of nociplastic pain severity [46, 268] and have been used to predict poor responsiveness to surgery and opioids in patients undergoing procedures such as arthroplasty [269] and hysterectomy [270].

Several additional instruments are available to screen for commonly associated nonpain symptoms, including depression, anxiety, and sleep disturbances. These include, for example, the Patient Health Questionnaire‐9 [213], the Generalized Anxiety Disorder‐7 Scale [214], the Insomnia Severity Index [216], and Patient‐Reported Outcomes Measurement Information System from National Institutes of Health.

Though there currently lacks questionnaires differentiating mechanisms causing different subtypes of nociplastic pains (i.e., top‐down or bottom‐up) [12], ongoing efforts have been made to establish and evaluate the types and combinations of features effective in distinguishing nociplastic pains of different mechanisms [209, 218, 265, 271]. For example, the 2019 introduction of a structured grading system for nociplastic pain affecting the musculoskeletal system offered clinicians a more nuanced understanding of the underlying mechanisms, thereby supporting better‐informed treatment decisions [218].

In summary, the contemporary diagnosis of chronic pain is a multifaceted process that integrates clinical assessment with mechanism‐specific tools. Nociceptive pain is identified through localized pain descriptors, questionnaires assessing sensory qualities, and laboratory markers of tissue injury or inflammation. Neuropathic pain diagnosis is supported by screening tools with high diagnostic accuracy, complemented by objective confirmatory tests such as neurophysiology, functional neuroimaging, and skin biopsy to verify somatosensory system lesions. The diagnosis of nociplastic pain remains primarily clinical, relying on the recognition of a characteristic syndrome, widespread pain with associated somatic and cognitive symptoms, aided by questionnaires that capture central sensitization features and comorbid conditions (Figure 4). A critical limitation across all types is the current lack of a single, definitive biomarker. Diagnosis therefore depends on a convergence of evidence from history, examination, and complementary investigations. This rigorous, stratified diagnostic approach is fundamental to differentiating pain mechanisms, guiding appropriate and targeted treatment, and advancing toward a more precise and personalized model of pain medicine.

## 6 Therapeutics Strategies: From Symptom Control to Precision Medicine

Effective management of chronic pain necessitates a tailored, mechanism‐based strategy that fundamentally differs across its three primary subtypes, that is, nociceptive, neuropathic, and nociplastic. This section systematically details the hierarchical, multimodal therapeutic frameworks for each subtype, with clinical trials summarized in Table 2. For nociceptive pain, treatment progresses from foundational analgesics (e.g., NSAIDs, paracetamol) to interventional procedures, emphasizing the mitigation of tissue injury and inflammation. Neuropathic pain management is anchored by first‐line neuromodulators (e.g., gabapentinoids, antidepressants), with advanced neuromodulation reserved for refractory cases. In stark contrast, the management of nociplastic pain prioritizes nonpharmacological, biopsychosocial interventions, patient education, graded exercise, and cognitive‐behavioral therapy (CBT), as first‐line care, with pharmacotherapy playing a secondary, adjunctive role. Across all subtypes, psychological approaches and emerging experimental treatments are integral to addressing the multidimensional nature of chronic pain and overcoming the limitations of current modalities.

**TABLE 2:** Exemplified clinical trials for major chronic pain therapeutics.

Pain subtype	Therapeutic line	Drug class	Example medication	Mechanism	Primary indication	NCT, status, phase	Objective	Preliminary finding	References
Nociceptive pain	First‐line	Simple analgesics	Paracetamol	Analgesic and antipyretic effects; exact mechanism is complex but distinct from COX inhibition. Favorable safety profile at recommended doses, but risk of hepatotoxicity with overdose	Mild‐to‐moderate nociceptive pain management	NCT00699114, completed, Phase IV	To establish the multidimensional pharmacodynamic profiles of commonly used analgesics (including paracetamol 500 and 1000 mg) in a homogeneous acute postoperative pain model, and to compare them with calculated effect size estimates (e.g., NNT)	Paracetamol has limited analgesic efficacy with a flat dose–response profile and is less effective than ibuprofen; however, its combination with codeine achieves similar analgesia to ibuprofen 400 mg with a more rapid onset.	[272]
Nociceptive pain	First‐line	NSAIDs	Naproxen, diclofenac	Peripheral inhibition of COX enzymes, reducing prostaglandin‐mediated inflammation and nociceptor sensitization	Inflammatory components of nociceptive pain (e.g., musculoskeletal injuries, osteoarthritis)	NCT00171665	To evaluate the efficacy and safety of topical 1% diclofenac sodium gel applied four times daily compared with vehicle (placebo) in reducing pain and improving function in patients with primary hand osteoarthritis over an 8‐week period	Topical diclofenac gel demonstrated significant superiority over placebo across all primary efficacy outcomes in hand osteoarthritis, with early and sustained improvement that reached clinical relevance. It was well tolerated, with mostly mild application‐site paresthesia and no systemic gastrointestinal or cardiovascular adverse events, highlighting the safety advantage of local administration.	[273]
			Ibuprofen			NCT03441269, completed, Phase IV	To compare analgesic efficacy of three single‐dose oral ibuprofen regimens in adult ED patients with acute pain, using pain score difference at 60 min as the primary outcome	The study found no significant difference in short‐term analgesic efficacy among 400, 600, and 800 mg ibuprofen for acute pain in the ED, with no adverse events reported. Thus, 400 mg ibuprofen is as effective as higher doses for initial acute pain management in this setting.	[274]
			Naproxen			NCT02388191, completed, NA	To evaluate whether 550 mg oral naproxen sodium given 1 h before IUD insertion is effective for pain relief during the insertion procedure compared with placebo	Naproxen sodium did not reduce pain during IUD insertion but significantly reduced postinsertion pain, supporting its use as a premedication for postprocedural analgesia.	[275]
Nociceptive pain	First‐line	Opioid analgesics	Codeine	Agonism of μ‐opioid receptors at presynaptic and postsynaptic terminals in the spinal cord and brain (e.g., periaqueductal gray, dorsal horn). This inhibits presynaptic Ca^2^^+^ influx (reducing neurotransmitter release like substance P) and enhances postsynaptic K^+^ efflux (causing neuronal hyperpolarization), attenuating nociceptive transmission.	Short‐term use for acute moderate‐to‐severe pain (e.g., postoperative, posttrauma). Risk of respiratory depression (via brainstem μ‐receptors), dependence, and misuse necessitates cautious, time‐limited use	NCT02625753, completed, Phase III	To assess the efficacy and safety of the codeine/acetaminophen combination as add‐on therapy for pain after PRK	Superior analgesia with typical opioid side effects: effective for post‐PRK pain but associated with drowsiness and nausea	[276]
			Tramadol			NCT03108482, completed, Phase III	To evaluate the analgesic efficacy and safety of the novel celecoxib–tramadol cocrystal versus its components (tramadol, celecoxib) and placebo in patients with moderate‐to‐severe acute pain following bunionectomy	The celecoxib–tramadol cocrystal provided significantly superior analgesia compared with its individual components and placebo, with a lower incidence of adverse events than tramadol alone and no serious safety concerns.	[277]
			Oxycodone			NCT02604446, completed, Phase III	To compare the analgesic effectiveness of tapentadol ER, oxycodone CR, and placebo as add‐ons to multimodal analgesia in the first week after total knee arthroplasty	Compared with oxycodone CR, tapentadol ER provided equivalent analgesia but with significantly less constipation as an add‐on after total knee arthroplasty.	[278]
Nociceptive pain	Second‐line	Weak opioid combinations	Paracetamol + codeine	Combined mechanisms: paracetamol (central analgesia) + opioid (μ‐receptor agonism).	Used when first‐line options provide insufficient relief	NCT03049878, completed, Phase IV	To determine the efficacy of a single preoperative oral dose of paracetamol‐codeine versus placebo for acute pain after impacted mandibular third molar surgery, using a randomized split‐mouth design	A single preoperative dose of paracetamol–codeine effectively reduced early postoperative pain and delayed its onset, though it did not decrease overall analgesic use. It serves as an effective preemptive analgesic strategy.	[279]
			Paracetamol + tramadol			NCT04178109, completed, Phase II	To compare oral tramadol/dexketoprofen vs. intravenous paracetamol + tramadol for posthip replacement pain relief and tolerability	Oral tramadol/dexketoprofen provided faster and superior analgesia with less need for rescue medication compared with IV paracetamol + tramadol after total hip arthroplasty, with both regimens well tolerated.	[280]
Nociceptive pain	Second‐line	Selective COX‐2 inhibitors	Celecoxib	Selective inhibition of the COX‐2 enzyme, reducing inflammatory prostaglandins with potentially lower GI risk than nonselective NSAIDs	Considered for persistent inflammatory pain in patients at high gastrointestinal risk, with cardiovascular risk evaluation required	NCT02934191, completed, Phase II	To assess high‐dose celecoxib versus placebo for pain control and safety after pediatric tonsillectomy	High‐dose celecoxib is a safe and effective analgesic after pediatric tonsillectomy, providing an opioid‐sparing effect that is more pronounced in children with higher pain levels, and can be considered an alternative to ibuprofen.	[281]
			Etoricoxib			NCT02534610, completed, NA	To compare preoperative versus postoperative etoricoxib for analgesia and morphine‐sparing effect in the first 48 h after total knee arthroplasty	Etoricoxib reduced postoperative morphine use by approximately 30%. Preoperative administration provided a superior opioid‐sparing effect compared with postoperative timing, with no increase in side effects.	[282]
Nociceptive pain	Second‐line	SNRIs	Duloxetine	Inhibits reuptake of serotonin and norepinephrine, enhancing descending inhibitory pain pathways in the spinal cord	Chronic low back pain with a neuropathic component	NCT02208778, recruiting, Phase IV	To explore the neural mechanisms of duloxetine‐induced pain relief and identify neuroimaging biomarkers predictive of treatment response in chronic knee osteoarthritis pain	This mechanistic study employs multimodal neuroimaging to elucidate the central nervous system mechanisms of duloxetine analgesia and to identify objective biomarkers predictive of treatment response, aiming to address the ∼50% responder rate in clinical practice.	[283]
Nociceptive pain	Second‐line	Gabapentinoids	Gabapentin	Bind to the α2δ subunit of voltage‐gated calcium channels on presynaptic neurons, modulating calcium influx and reducing the release of excitatory neurotransmitters involved in central sensitization	Primarily for neuropathic pain, but also used in perioperative settings and certain chronic nociceptive states (e.g., fibromyalgia)	NCT02725710, completed, Phase II	To evaluate the effect of adding oral gabapentin to a usual oral pain management regimen on pain 5 min after surgical abortion	Gabapentin did not provide additional acute analgesia but significantly reduced postprocedure opioid consumption, with a comparable safety profile to placebo.	[284]
			Pregabalin						
Nociceptive pain	Second‐line	Cannabinoid‐based medications	THC/CBD formulations (e.g., oromucosal spray)	Activation of cannabinoid receptors CB1 (predominantly CNS) and CB2 (mainly peripheral immune cells). CB1 activation inhibits presynaptic Ca^2^^+^ influx, suppressing excitatory neurotransmitter release (e.g., glutamate, substance P). CB2 activation modulates immune response and neuroinflammation.	Promising adjunct for conditions like diabetic neuropathy and multiple sclerosis‐related neuropathic pain. Treatment often initiates with CBD‐predominant formulations (THC:CBD ≤1:10) to minimize psychoactive effects.	NA, completed, NA	To assess the long‐term safety, tolerability, and clinical benefit of THC/CBD spray in patients with advanced cancer‐related pain	Long‐term use of THC/CBD spray was safe and well tolerated. It provided sustained pain relief and improved quality of life in advanced cancer pain without leading to tolerance or increased analgesic requirements, supporting its role as an effective long‐term adjuvant therapy.	[285]
Nociceptive pain	Second‐line	Interventional procedures	Corticosteroid injections	Pharmacological (injections): local anti‐inflammatory (steroids) or signal blockade (anesthetics). Ablative/stimulatory: thermocoagulation of nociceptive fibers or electrical modulation (SCS activates Aβ fibers, enhancing GABAergic inhibition and descending pathways without opioid receptors)	For well‐localized, refractory pain; provides diagnostic/therapeutic benefit (e.g., for radicular pain, osteoarthritis, failed back surgery syndrome)	NCT02580630, completed, Phase IV	To compare ultrasound‐guided corticosteroid injection vs. placebo injection, both combined with exercise therapy, for chronic Achilles tendinopathy at 6 months	Corticosteroid injection combined with exercise therapy was more effective than placebo plus exercise for chronic Achilles tendinopathy, with a favorable safety profile and no long‐term deterioration.	[286]
			Nerve blocks (local anesthetics)			NCT03706313, completed, Phase IV	To assess if adding a genicular nerve block to standard analgesia reduces 24‐h opioid use after total knee arthroplasty compared with sham block	Adding a genicular nerve block to a comprehensive analgesic regimen significantly reduces early postoperative opioid requirements and improves early analgesia after total knee arthroplasty, without compromising patient satisfaction or functional recovery.	[287]
			Radiofrequency ablation			NCT04786145, NA, NA	To compare cryoneurolysis and radiofrequency ablation versus placebo on patient‐reported outcomes over 1 year in patients with facetogenic chronic low back pain	Neither cryoneurolysis nor radiofrequency ablation demonstrated superior clinical benefit over a placebo procedure for facetogenic chronic low back pain in short‐ or long‐term follow‐up across all patient‐reported outcomes, including global impression of change, pain intensity, function, and quality of life.	[288]
			SCS			NCT03594266, completed, NA	To assess the safety and efficacy of a novel multiphase spinal cord stimulation (two frequency ranges) in SCS‐responsive patients with chronic low back/leg pain	Multiphase SCS safely and effectively reduced chronic low back/leg pain, with comparable efficacy between its two frequency settings. It may offer superior analgesia and greater energy efficiency than traditional SCS, warranting long‐term evaluation.	[289]
Nociceptive pain	Psychological approaches	CBT	Nonpharmacological	Identifies and modifies maladaptive pain‐related thoughts (catastrophizing), behaviors (avoidance), and emotional responses (fear, anxiety) to improve coping and function	Integral part of managing chronic nociceptive pain, addressing the biopsychosocial model; effectively reduces perceived pain intensity, disability, and distress	NCT01467843, completed, Phase III	To compare the effectiveness of MBSR and CBT versus usual care for improving back pain and function in adults with chronic low back pain	Both MBSR and CBT were more effective than usual care in improving pain and function in chronic low back pain, with comparable efficacy between the two psychological interventions. The benefits of MBSR were sustained at 1 year.	[290]
Nociceptive pain	Psychological approaches	ACT	Nonpharmacological	Enhances psychological flexibility—accepting pain sensations while committing to value‐driven actions		NCT03105908, completed, NA	To identify predictors of treatment effect in digital ACT for chronic pain by analyzing pooled data from prior trials	Shorter pain duration and more severe baseline insomnia predicted better outcomes from digital ACT for chronic pain. These findings help identify patients most likely to benefit, aiding personalized treatment recommendations.	[291]
Nociceptive pain	Psychological approaches	MBSR	Nonpharmacological	Trains nonjudgmental awareness of present‐moment sensations (including pain), reducing suffering and emotional reactivity		NCT02133976, completed, NA	To compare CT, MBSR, and BT versus TAU for chronic low back pain, evaluating effects on pain, function, mood, sleep, and the durability/speed of response	CT, MBSR, and BT were equally effective and superior to usual care for chronic low back pain, with durable benefits and similar speed of action.	[292]
Neuropathic pain	First‐line	Gabapentinoids	Gabapentin	Bind to the α2δ subunit of presynaptic voltage‐gated calcium channels, reducing calcium influx, neurotransmitter release, and neuronal hyperexcitability	Diabetic neuropathy, postherpetic neuralgia, spinal cord injury pain; adverse effects: sedation, dizziness, weight gain, risk of misuse	NCT01127100, completed, Phase IV	To assess the noninferiority of transdermal fentanyl versus gabapentin for efficacy and safety in chronic radicular neuropathic pain over 56 days	Transdermal fentanyl was noninferior to gabapentin for pain relief in chronic radicular neuropathic pain, with comparable effects on function, mood, and overall safety. Gabapentin was associated with a higher tendency for dizziness and somnolence. The study supports fentanyl as an effective alternative treatment option.	[293]
			Pregabalin			NCT05624853, completed, Phase IV	To demonstrate the noninferiority of once‐daily sustained‐release pregabalin compared with twice‐daily immediate‐release pregabalin for diabetic neuropathic pain over 8 weeks	Extended‐release pregabalin provides comparable pain relief and safety to the immediate‐release version, offering a once‐daily, more convenient treatment option for diabetic peripheral neuropathic pain.	[294]
Neuropathic pain		TCAs	Amitriptyline	Potently inhibit serotonin and norepinephrine reuptake pumps, enhancing descending inhibitory pain pathways; also provide sodium channel blockade and NMDA receptor antagonism	Postherpetic neuralgia, painful diabetic neuropathy; adverse effects: anticholinergic effects (dry mouth, constipation), sedation, cardiac (QTc prolongation); use limited in elderly	NCT00370656, completed, Phase II/III	To compare the analgesic efficacy and the effects on sleep, daytime function, and quality of life of pregabalin, amitriptyline, and duloxetine in patients with DPNP	The three medications showed similar analgesic efficacy, but with distinct profiles: pregabalin improved sleep continuity, while duloxetine enhanced daytime alertness and performance despite reducing total sleep time. Adverse events were more frequent with pregabalin, though no major safety concerns emerged.	[295]
			Nortriptyline			ISRCTN73178636, completed, NA	To evaluate the analgesic efficacy and safety of a nortriptyline–morphine combination compared with each drug as monotherapy in patients with neuropathic pain	The nortriptyline–morphine combination demonstrated superior analgesic efficacy over either drug alone. Its safety profile reflected the combined adverse effects of both components, primarily constipation and dry mouth.	[296]
Neuropathic pain		SerotonSNRIs	Duloxetine	Dual inhibition of serotonin and norepinephrine reuptake, amplifying brainstem‐spinal descending pain inhibitory systems	Painful diabetic peripheral neuropathy, fibromyalgia; advantage over TCAs: better tolerability, fewer anticholinergic/cardiac effects	NCT00489411, completed, Phase III	To evaluate the effect of duloxetine (60 mg/day) on pain, function, and quality of life in patients with chemotherapy‐induced painful peripheral neuropathy	Duloxetine provided significantly greater pain relief than placebo, with more patients reporting pain reduction.	[297]
			Venlafaxine			IRCT2015060922632N1, completed, NA	To compare the effects of venlafaxine versus duloxetine on the symptoms of chemotherapy‐induced peripheral neuropathy	Duloxetine showed greater efficacy than venlafaxine in alleviating chemotherapy‐induced neuropathy symptoms.	[298]
Neuropathic pain	Second‐line	Topical agents	Lidocaine (5% patch)	Lidocaine: blocks peripheral sodium channels, reducing nerve firing; capsaicin: binds and activates/desensitizes the TRPV1 receptor on nociceptors, causing defunctionalization	Localized neuropathic pain (e.g., postherpetic neuralgia, diabetic neuropathy); offer localized effect with minimal systemic exposure	NCT02763592, completed, Phase II	To evaluate the efficacy and safety of a 5% lidocaine plaster for post‐knee surgery neuropathic pain, focusing on allodynia, hyperalgesia, and thermal sensitivity	The lidocaine plaster was effective in alleviating allodynia and improving pain thresholds in patients with postsurgical neuropathic knee pain.	[299]
			Capsaicin (8% patch, creams)			NCT02228928, completed, Phase I/II	To compare the efficacy and safety of topical capsaicin patches versus cream and placebo in peripheral neuropathic pain	The 0.625% capsaicin patch provided significant pain relief and favorable patient feedback, with localized skin irritation as the main side effect, positioning it as a potentially safer alternative to high‐concentration (8%) patches.	[300]
Neuropathic pain		Opioids	Tramadol	Dual mechanism: μ‐opioid receptor agonism combined with inhibition of serotonin/norepinephrine reuptake	Refractory neuropathic pain (short‐term use); risks: dependence, respiratory depression, abuse. Efficacy may be reduced in chronic pain due to neuroadaptive changes.	NCT02722603, terminated, Phase III	To compare the efficacy, safety, and pharmacokinetics of gabapentin versus tramadol in pediatric neuropathic/mixed pain	Final outcomes comparing gabapentin to tramadol are pending; primary outcome is the difference in average pain scores at 15 weeks.	[301]
			Tapentadol			NCT01352741, completed, Phase IV	To evaluate tapentadol PR monotherapy versus combined tapentadol PR/pregabalin therapy in severe neuropathic low back pain	Tapentadol PR monotherapy provided comparable pain relief to the tapentadol‐pregabalin combination while demonstrating superior CNS tolerability.	[302]
Neuropathic pain		BoNT‐A	OnabotulinumtoxinA	Proteolytically cleaves SNAP‐25, inhibiting vesicle fusion and release of pain mediators (glutamate, substance P, CGRP) from nociceptors	Postherpetic neuralgia, diabetic neuropathy, trigeminal neuralgia; administered via localized injection; limitations: requires specialized training, repeated applications, cost	NCT00336349, completed, NA	To assess the effect of onabotulinumtoxinA on sensory function in painful diabetic polyneuropathy	OnabotulinumtoxinA significantly and sustainably improved both tactile and pain sensitivity in diabetic neuropathy patients over 12 weeks.	[303]
Neuropathic pain	Third‐line	NMDA receptor antagonists	Ketamine (IV)	Antagonize NMDA receptors, blocking glutamate‐driven calcium influx and reducing central sensitization and synaptic hyperexcitability	Refractory conditions (e.g., complex regional pain syndrome, diabetic neuropathy); limitations: ketamine has psychotomimetic risks; dextromethorphan has variable efficacy and side effects (drowsiness).	NCT02467517, NA, Phase II	To assess the 5‐week analgesic and cognitive‐emotional effects of intravenous ketamine alone and combined with magnesium versus placebo in refractory neuropathic pain	The study found no significant benefit of ketamine (with or without magnesium) over placebo across all measured outcomes, with adverse events being most common in the combination group.	[304]
			Dextromethorphan (oral)			NCT02271893, terminated, Phase II	To evaluate dextromethorphan's effects on pain, cognition, and quality of life in breast cancer patients with CIPN, and the role of genetic polymorphism in its analgesic response	This is a protocol hypothesizing dextromethorphan may be effective for CIPN, with pharmacogenetics influencing response. Results are pending.	[305]
Neuropathic pain		Cannabinoids	Plant‐derived/synthetic THC/CBD formulations	Activate cannabinoid receptors (CB1/CB2); modulate neurotransmitter release and neuronal excitability	Refractory neuropathic pain; evidence: modest average pain reduction vs. placebo; considerations: psychoactive potential, variable efficacy; generally reserved for refractory cases	NCT00710554, completed, Phase III	To assess the efficacy and safety of THC/CBD oromucosal spray as add‐on therapy for treatment‐resistant peripheral neuropathic pain with allodynia	THC/CBD spray was superior to placebo in the proportion of patients achieving meaningful pain relief (≥30% reduction) and improved sleep quality, though the reduction in mean pain scores was not significant. The treatment was well tolerated.	[306]
Neuropathic pain	Interventional procedures	Injections/blocks	Epidural steroid injections	Pharmacological blockade (steroids reduce inflammation; local anesthetics block nerve conduction) to interrupt pain signaling	Radicular pain (e.g., sciatica), complex regional pain syndrome; used for diagnostic and therapeutic relief	NCT03995563, completed, Phase IV	To compare the efficacy and safety of single‐dose versus intermittent repeated epidural dexamethasone (with local anesthetic) in severe Zoster‐associated pain	Intermittent epidural dexamethasone provided superior pain relief and remission rates versus a single dose, and was well tolerated.	[307]
			Sympathetic nerve blocks			NCT02737527, completed, NA	To compare the procedural efficacy and safety of ultrasound‐assisted versus fluoroscopy‐guided lumbar sympathetic ganglion blocks	Both techniques were equally effective and safe, but ultrasound guidance offered key advantages: lower radiation, less procedural bone contact, and faster blockade onset.	[308]
Neuropathic pain		Neuromodulation	Spinal cord stimulation (SCS)	Electrical modulation: SCS activates dorsal column Aβ fibers, enhancing GABAergic inhibition; PNS directly targets peripheral nerves.	Refractory neuropathic pain (e.g., failed back surgery syndrome); definitive interventional treatment	ISRCTN77527324, completed, NA	To compare the effectiveness of adding spinal cord stimulation to conventional medical management versus conventional medical management alone for neuropathic pain in failed back surgery syndrome	SCS was more effective than standard care for failed back surgery syndrome pain, but involved device complications; most control patients eventually received SCS.	[309]
			PNS			NCT01996254, completed, NA	To evaluate percutaneous PNS against placebo for treating chronic neuropathic pain after amputation	Percutaneous PNS provided significantly greater and sustained pain relief compared with placebo for postamputation neuropathic pain, with evidence suggesting durable long‐term benefit.	[310]
Neuropathic pain		Other	TENS	TENS: low‐voltage surface currents modulate superficial nerve activity; surgery: addresses anatomic nerve compression	Noninvasive alternative (TENS) or specific entrapment neuropathies (surgery)	NCT04509518, completed, NA	To assess the effectiveness of adding TENS to physical therapy for pudendal neuralgia in male patients	Adding TENS to physical therapy significantly improved pain relief and reduced analgesic use compared with physical therapy alone in pudendal neuralgia.	[311]
			Surgical decompression			NCT02602093, NA, NA	To assess the noninferiority of PTED versus open microdiscectomy in reducing leg pain from lumbar disc herniation	PTED was noninferior to open surgery for pain relief and demonstrated significant advantages in recovery speed and resource use, with similar safety.	[312]
Neuropathic pain	Psychological approaches	Psychotherapy	CBT	CBT: restructures catastrophic thinking, reduces fear‐avoidance; MBSR/ACT: promotes nonjudgmental acceptance of pain, engagement in valued activities	Fundamental for comorbid mood disorders and disability; aims to reduce suffering, improve function, and enhance treatment effectiveness	NCT00830011, completed, NA	To compare the efficacy of CBT versus as adjunctive treatments for diabetic peripheral neuropathic pain	Both interventions improved outcomes, but CBT demonstrated specific advantages: greater reduction in neuropathic pain intensity at 12 weeks, and superior long‐term (36‐week) improvements in pain interference and mental health functioning compared with diabetes education.	[313]
			Mindfulness‐based stress reduction (MBSR)			NCT02127762, completed, NA	To examine the psychological mechanisms by which MBSR improves pain outcomes in patients with PDPN	Increased mindfulness, not pain catastrophizing, mediated MBSR's benefits on pain and function in PDPN. Patients with longer diabetes duration may experience greater gains through enhanced mindfulness.	[314]
			ACT			NCT03584412, NA, NA	To examine the feasibility of a full‐scale RCT testing online ACT for neuropathic pain in people living with HIV	Findings support the feasibility and acceptability of a future definitive trial for ACT OPEN, with promising preliminary signals for improving pain and depression in this population.	[315]
Nociplastic pain	First‐line (nonpharmacological)	Patient education and self‐management	PNE	Biopsychosocial model: explains central sensitization to validate experience, reduce fear, and shift perspective; behavioral skills: teaches activity pacing to avoid “boom‐bust” cycles and promotes self‐efficacy	Cornerstone of management; aims to empower patients and provide practical self‐management tools	NCT05736172, NA, NA	To evaluate the effects of a 3‐h PNE session on BDNF levels and pain intensity in chronic pain patients	Despite not altering BDNF levels, PNE significantly reduced pain intensity and improved key psychological variables in chronic pain patients.	[316]
			Pacing			NCT03497585, completed, NA	To test the feasibility of implementing a new activity pacing framework and explore its effects on pacing behavior and symptoms	The framework was feasible and well received, with significant improvements in pacing ability and symptoms (pain, fatigue) postintervention. Gains were partially maintained at 3 months, though attrition was notable.	[317]
			Stress and sleep management			NCT03239938, completed, NA	To compare the efficacy of the modern pain neuroscience approach versus usual care physiotherapy for reducing disability in chronic whiplash‐associated disorders	MPNA outperformed usual care at posttreatment and long‐term follow‐up, improving key pain‐related mechanisms and outcomes, despite no difference at the 6‐month primary endpoint.	[318]
Nociplastic pain		Prescribed exercise	GA exercise (walking, swimming, cycling)	Physiological: strengthens descending inhibitory pathways, increases parasympathetic tone, may reduce inflammation; behavioral: counters fear‐avoidance, builds self‐efficacy, supports adaptive neuroplasticity	One of the most evidence‐supported interventions; must be graded and consistent; alone may be insufficient for moderate‐severe symptoms	NCT04023162, completed, NA	To compare a biopsychosocial exercise approach (GA) with traditional supervised exercise for chronic low back pain	The biopsychosocial approach (GA) provided more sustained long‐term benefits than traditional exercise therapy alone.	[319]
			Mind‐Body Practices (Tai Chi, Yoga)			NCT03412916, completed, NA	To investigate the relationship between home practice adherence and physical/emotional outcomes in a mind–body intervention for chronic pain	Patients were generally adherent to home practice. Emphasizing longer duration of physical activity may be beneficial for improving depression in this context.	[320]
Nociplastic pain		Psychological therapies	CBT‐P	CBT‐P: restructures catastrophic thoughts, reduces fear‐avoidance behaviors, uses behavioral activation; ACT: fosters psychological flexibility (acceptance + value‐driven action); MBSR: cultivates nonreactive awareness to reduce emotional suffering	First‐line psychological intervention; addresses cognitive, emotional, and behavioral aspects of pain. For some, trauma‐informed therapies (e.g., emotional exposure) may be needed.	NCT01129817, completed, NA	To compare the long‐term (3‐year) efficacy of cognitive functional therapy versus manual therapy and exercise for disability and pain in chronic low back pain	Cognitive functional therapy was superior to manual therapy and exercise for long‐term improvement in function and psychological factors, but not pain intensity, indicating a dissociation between pain and disability.	[321]
			ACT			NCT04140838, completed, NA	To evaluate the efficacy of remote group ACT and BATD as adjuncts to usual care for chronic low back pain and co‐occurring depression	ACT and BATD outperformed usual care for pain‐related outcomes (not mood), with psychological flexibility driving improvements.	[322]
			MBSR			NCT04304664, completed, NA	To assess MBSR's effects in fibromyalgia and explore the mediating role of pain‐related cognitions	MBSR has significant therapeutic potential for fibromyalgia, and its benefits are driven by changes in specific pain‐related cognitions.	[323]
Nociplastic pain	Second‐line	Pharmacological agents	SNRIs (duloxetine)	SNRIs/TCAs: enhance descending inhibitory (serotonin/norepinephrine) pathways; gabapentinoids: modulate voltage‐gated calcium channels to reduce neuronal hyperexcitability	Pharmacotherapy is second‐line. SNRIs have best evidence. Opioids are contraindicated long‐term (risk of hyperalgesia, no efficacy). COX‐2 inhibitors/acetaminophen are not effective.	NCT03880916, NA, NA	To determine whether duloxetine can reduce postoperative pain and improve quality of recovery after TKA in patients with pre‐existing central sensitization	Duloxetine effectively reduces pain and improves recovery in centrally sensitized patients undergoing TKA.	[324]
			SNRIs (milnacipran)			NCT01747044, NA, Phase II	To evaluate whether milnacipran could improve the status of CPM, a measure of central pain inhibition, in patients with fibromyalgia	Milnacipran did not significantly improve pain outcomes compared with placebo after 1 month, though a trend suggested possible benefit in a subgroup of patients.	
			TCAs (amitriptyline)			ACTRN12612000131853, completed, Phase II	To examine the efficacy of low‐dose amitriptyline versus an active comparator on pain, disability, and work outcomes in chronic low back pain	The trial suggests amitriptyline may be an effective treatment, showing a short‐term functional benefit and good tolerability, though it did not meet its primary efficacy endpoint.	[325]
			Gabapentinoids (pregabalin)			NCT00645398, completed, Phase III	To evaluate pregabalin for pain and sleep in fibromyalgia patients with moderate‐to‐severe baseline pain	Pregabalin effectively reduced pain and improved sleep versus placebo, especially in severe fibromyalgia, with a favorable safety profile.	[326]
			Gabapentinoids (gabapentin)			NCT02918760, NA, Phase III	To evaluate the efficacy of oral Gabapentin in alleviating pain in women with chronic pelvic pain	The study concluded that gabapentin was an effective treatment for reducing chronic pelvic pain, with a safety profile consistent with its known side effects.	[327]
Nociplastic pain		Physical modalities	Acupuncture	Acupuncture: activates Aδ/C fibers, releases endogenous opioids and monoamines, modulates limbic brain activity (ACC, amygdala), may restore oxidative homeostasis.; acupressure: manual stimulation of points; chiropractic: aims to reduce aberrant peripheral nociceptive input.	Evidence base varies; must be integrated into a broader biopsychosocial plan; high‐velocity manipulation is risky (may exacerbate symptoms); gentler approaches are recommended.	ChiCTR2100044762, completed, NA	To assess the efficacy of acupuncture in patients with IBS‐D	Acupuncture outperformed sham treatment with lasting efficacy and no severe safety concerns.	[328]
			Acupressure			NCT02106741, completed, NA	To examine the preliminary effects of relaxing and stimulating self‐administered acupressure on multiple symptoms in chronic low back pain	Self‐acupressure safely reduced pain and (with stimulating technique) fatigue.	[329]
			Chiropractic manipulation (gentle, nonthrust)			ACTRN12613000653763, completed, NA	To assess the feasibility and preliminary effects of instrument‐assisted chiropractic versus sham treatment for dizziness and neck pain in older adults	A feasibility study that confirmed methodological viability, hinted at treatment benefit, and powered a future definitive trial.	[330]
Nociplastic pain	Third‐line (interventional for refractory cases)	Neurostimulation techniques	tDCS	tDCS/rTMS: modulate cortical excitability (e.g., motor cortex, DLPFC) to reinforce descending inhibitory control; VNS: activates parasympathetic system, may suppress proinflammatory signaling; TENS: applies peripheral current for segmental spinal inhibition (gate‐control) and possible supraspinal modulation	Considered for symptoms refractory to conventional therapies; evidence is evolving; represents an expanding advanced therapeutic arsenal	NCT05231239, completed, Phase II	To investigate pain reduction via anodal transcranial direct current stimulation (tDCS) in patients with endometriosis and chronic pelvic pain	tDCS is an effective supporting therapy, supporting the central nervous system's role in the maintenance of chronic pelvic pain.	[331]
			rTMS			ChiCTR2200061399, NA, NA	To investigate the analgesic efficacy and brain activation patterns of rTMS combined with rPMS in patients with CNLBP	The combined rTMS and rPMS therapy provided specific benefits by increasing C‐fiber pain thresholds and promoting functional remodeling in key brain pain‐processing regions.	[332]
			VNS			NCT00294281, completed, Phase II	To assess the safety, tolerability, and preliminary efficacy of VNS in treatment‐resistant fibromyalgia	The treatment demonstrated a known safety profile and was generally well tolerated, though not by all. It induced clinically significant and profound improvement in a subset of patients, with benefits showing a positive trend over time.	[333]
			TENS			NCT01017913, completed, NA	To compare the effects of TENS and IFC among patients with nonspecific chronic low back pain	Both electrotherapies were effective and equivalent for pain and function, with strong associated reductions in medication use.	[334]

*Abbreviations*: ACC, anterior cingulate cortex; ACT, acceptance and commitment therapy; BATD, behavioral activation therapy for depression; BDNF, brain‐derived neuropathic factor; BoNT‐A, botulinum toxin type A; CBT, cognitive‐behavioral therapy; CBT‐P, cognitive‐behavioral therapy for pain; CGRP, calcitonin gene‐related peptide; CIPN, chemotherapy‐induced peripheral neuropathy; CNLBP, chronic nonspecific low back pain; CNS, central nervous system; COX, cyclooxygenase; COX‐2, cyclooxygenase‐2; CPM, conditioned pain modulation; DLPFC, dorsolateral prefrontal cortex; DPNP, diabetic peripheral neuropathic pain; ED, Emergency Department or Diabetes Education; ER, extended‐release or controlled‐release; GA, graded activity; GI, gastrointestinal; IBS‐D, irritable bowel syndrome with diarrhea; IFC, interferential current; IUD, intrauterine device; MBSR, mindfulness‐based stress reduction; MPNA, multimodal pain neuroscience and acceptance; NA, not available; NNT, number needed to treat; NSAIDs, nonsteroidal anti‐inflammatory drugs; PDPN, painful diabetic peripheral neuropathy; PNE, pain neuroscience education; PNS, peripheral nerve stimulation; PPK, pain after photorefractive keratectomy; PR, prolonged release; PTED, percutaneous endoscopic discectomy; rPMS, repetitive peripheral magnetic stimulation; rTMS, repetitive transcranial magnetic stimulation; SCS, spinal cord stimulation; SNRIs, serotonin–norepinephrine reuptake inhibitors; TCAs, tricyclic antidepressants; tDCS, transcranial direct current stimulation; TENS, transcutaneous electrical nerve stimulation; TKA, total knee arthroplasty; VNS, vagus nerve stimulation.

### 6.1 Nociceptive Pain

The management of nociceptive pain, which arises from the activation of nociceptors in response to actual or potential tissue damage, follows a stepwise, multimodal framework. This approach prioritizes safety and efficacy, beginning with foundational pharmacological interventions and escalating in complexity based on the pain's persistence, severity, and impact on function. The therapeutic strategy integrates pharmacological and nonpharmacological modalities to target both peripheral drivers and central processing of pain.

#### 6.1.1 First‐Line Pharmacological Options

The cornerstone of acute and mild‐to‐moderate nociceptive pain management is the use of simple analgesics and NSAIDs. Paracetamol (acetaminophen) is widely recommended for its analgesic and antipyretic effects, with a favorable safety profile at recommended doses, though hepatotoxicity risks necessitate dose caution. NSAIDs (e.g., ibuprofen, naproxen, diclofenac) are first‐line for inflammatory components of nociceptive pain (e.g., musculoskeletal injuries, OA) due to their peripheral inhibition of cyclooxygenase (COX) enzymes, thereby reducing PGE‐mediated inflammation and sensitization. Topical NSAIDs (e.g., diclofenac gel) offer a valuable option for localized pain (e.g., OA of the knee) with minimal systemic exposure. For acute moderate‐to‐severe pain, particularly postoperatively or posttrauma, short‐term use of opioid analgesics (e.g., codeine, tramadol, oxycodone) may be considered [335]. They produce analgesia primarily through agonism of μ‐opioid receptors located at both presynaptic and postsynaptic terminals within spinal cord circuits, resulting in the inhibition of neurotransmitter release and the attenuation of nociceptive transmission [336]. Among the three classical opioid receptor types (μ, δ, κ), the μ‐receptor is particularly critical for spinal and supraspinal analgesia, as evidenced by the absence of opioid analgesia in μ‐receptor knockout mice [337]. These receptors are abundantly expressed in key pain‐modulatory regions, including the PAG, raphe magnus, hypothalamus, and hippocampus, as well as in the dorsal horn of the spinal cord (Rexed laminae I, II, and V) [337, 338]. Endogenous opioid peptides, such as beta‐endorphin, enkephalins, and dynorphins, also bind to these receptors to inhibit the transmission of nociceptive signals, including those involving substance P. Their mechanisms include (1) inhibition of presynaptic calcium influx, reducing neurotransmitter release and (2) enhancement of potassium efflux, leading to neuronal hyperpolarization and suppression of action potential generation [337]. Both exogenous opioids and endogenous ligands act on peripheral and central terminals of nociceptors to elevate the firing thresholds and activate descending inhibitory pathways that block ascending pain signals. Beta‐endorphin serves as the principal endogenous ligand for μ‐receptors [338, 339]. However, the widespread distribution of opioid receptors also underlies serious adverse effects. For example, activation of μ‐receptors in the brainstem pre‐Bötzinger complex can disrupt respiratory rhythm generation and cause respiratory depression [338, 339]. Their use must be time limited, at the lowest effective dose, and with close monitoring. These risks, including respiratory depression, physical dependence, and the potential for long‐term misuse, have highlighted the need for alternative analgesics with improved safety profiles.

#### 6.1.2 Second‐Line Therapies

When first‐line options provide insufficient relief or are poorly tolerated, second‐line agents target different pain pathways. Weak opioid combinations (e.g., paracetamol with codeine or tramadol) are commonly employed. For persistent inflammatory or musculoskeletal pain, selective COX‐2 inhibitors (e.g., celecoxib, etoricoxib) may be considered for patients at high gastrointestinal risk, though cardiovascular risk profiles require evaluation. In specific conditions like chronic low back pain with a neuropathic component, 5‐HT–norepinephrine reuptake inhibitors (SNRIs) such as duloxetine have demonstrated efficacy, likely by enhancing descending inhibitory pathways. Similarly, the α2δ ligands (gabapentinoids like gabapentin and pregabalin), though primarily indicated for neuropathic pain, show utility in perioperative settings and certain chronic nociceptive states (e.g., fibromyalgia) by modulating calcium channels and central sensitization. Cannabinoid‐based medications have garnered increasing interest in pain management owing to their potential utility across diverse clinical conditions and a more favorable adverse effect profile compared with opioids [340, 341, 342]. These compounds modulate both central and peripheral pain signaling primarily via interactions with cannabinoid receptors CB1 and CB2. CB1 receptors are predominantly located in the CNS; their activation inhibits presynaptic calcium influx and suppresses the release of excitatory neurotransmitters such as glutamate and substance P, thereby reducing central sensitization [343]. CB2 receptors are mainly expressed on immune cells and in peripheral tissues; their activation helps regulate immune responses and neuroinflammation, the criticality of which has been gaining incremental recognition in the development and maintenance of neuropathic pain [343]. The analgesic mechanisms of cannabinoids involve both direct suppression of excitatory and inhibitory neurotransmitter release via synaptic vesicle modulation, and indirect modulation of neuronal excitability through inhibition of voltage‐gated ion channels [344]. The two primary active constituents, tetrahydrocannabinol (THC) and cannabidiol (CBD), act largely through CB1 and CB2 receptors, respectively [345]. Current consensus has been reached in initiating treatment with CBD‐predominant oral formulations, starting at 5 mg twice daily and titrating cautiously up to 40 mg daily, while maintaining a THC : CBD ratio not exceeding 1 : 10 to minimize psychoactive and other side effects [346]. Although evidence regarding the long‐term efficacy of cannabinoids remains limited [340, 341, 342], their unique mechanisms of action have positioned them as promising adjuncts to conventional analgesics. For instance, cannabinoids have demonstrated utility as adjuvants to first‐line treatments in diabetic neuropathy and multiple sclerosis‐related neuropathic pain [347]. A meta‐analysis reported that cannabinoids provided a small but significant reduction in pain compared with opioid therapy [348]. Further supporting their clinical relevance, a double‐blind, randomized, placebo‐controlled trial showed that a Δ9‐THC/CBD oromucosal spray significantly improved pain scores and sleep quality compared with placebo [306]. Such a novel cannabinoid formulation has subsequently been approved in several countries for treating cancer‐related pain and pain associated with multiple sclerosis [340].

For well‐localized, refractory nociceptive pain, targeted interventional procedures can provide significant diagnostic and therapeutic benefit. Corticosteroid injections (epidural, intra‐articular, bursal, or peri‐tendinous) are mainstays for inflammatory foci, such as in radicular pain, OA, or tendinopathies, providing potent local anti‐inflammatory effects. Nerve blockswith local anesthetics (e.g., facet joint injections, peripheral nerve blocks) can interrupt pain signal transmission for both acute analgesia and as part of a diagnostic workup. For chronic joint pain, radiofrequency ablation of the sensory nerves innervating the joint (e.g., genicular nerves for the knee) can provide medium‐to‐long‐term relief by thermocoagulation of nociceptive fibers, with evidence from randomized controlled trials supporting its efficacy for short‐term relief and benefits lasting beyond 6 months in a subset of patients [349, 350, 351]. Vertebroplasty or kyphoplasty are specialized procedures for painful vertebral compression fractures. For patients with refractory symptoms, surgical intervention (e.g., outpatient spine surgery) can provide sustained functional improvements [352]. For those who remain inadequately managed, advanced spinal cord stimulation (SCS) has emerged as a promising option. By epidurally stimulating Aβ fibers, SCS modulates nociceptive transmission via mechanisms rooted in the gate control theory, including enhanced GABAergic inhibition and activation of descending pathways, without engaging opioid receptors [353, 354, 355]. SCS has demonstrated efficacy in reducing pain and improving function in refractory conditions such as failed back surgery syndrome and diabetic neuropathy [356, 357].

#### 6.1.3 Psychological Approaches

Given the significant biopsychosocial interplay in all chronic pain, including nociceptive pain, psychological therapies are integral, not adjunctive. CBT is the most empirically supported approach, helping patients identify and modify maladaptive pain‐related thoughts (catastrophizing), behaviors (avoidance), and emotional responses (fear, anxiety) to improve coping and function. Acceptance and commitment therapy (ACT) focuses on enhancing psychological flexibility, accepting pain sensations while committing to value‐driven actions. Mindfulness‐based stress reduction (MBSR) trains patients in nonjudgmental awareness of present‐moment sensations, including pain, which can reduce suffering and emotional reactivity. These approaches effectively reduce perceived pain intensity, disability, and distress.

#### 6.1.4 Experimental Treatments

Experimental efforts for treating nociceptive pain have been devoted to explore a range of novel targets beyond traditional pharmacology, with NGF inhibitors being a prominent example. For instance, the monoclonal antibody tanezumab can selectively bind and inhibit NGF, a key driver of peripheral and central sensitization that is upregulated in conditions like OA to induce pain; substantial Phase III clinical trials have demonstrated its significant efficacy in reducing OA pain by blocking the interaction between NGF and the tropomyosin receptor kinase A [358, 359]; however, significant safety concerns regarding joint safety have emerged from these trials, where composite joint safety events (such as rapidly progressive OA Type 1) occurred at a dose‐dependent, statistically higher rate with tanezumab compared with NSAIDs, though adverse events like abnormal peripheral sensation were typically mild and transient [359].

Another molecular‐targeting approach involves the development of selective sodium channel blockers. For example, small‐molecule inhibitors can target the tetrodotoxin‐resistant channels Nav1.8 and Nav1.7 highly expressed in nociceptors; preclinical studies have shown that compounds like A‐803467 could potent inhibition of Nav1.8‐dependent action potentials in models of inflammatory pain [360]. However, a key translational challenge highlighted the concept of degeneracy in nociceptor excitability, where neurons can achieve similar firing patterns using different combinations of sodium channel subtypes (NaV1.3, NaV1.7, NaV1.8), and the dominant subtype can shift over time or after inflammation, potentially compromising the reliable efficacy of any single subtype‐selective inhibitor [361, 362].

Gene therapy and regenerative medicine represent another frontier aiming for long‐term disease modification. Strategies of this family include the use of viral vectors to deliver genes for endogenous opioids (e.g., preproenkephalin) or anti‐inflammatory cytokines (e.g., IL‐10) to the spinal cord or peripheral tissues to dampen pain signaling and inflammation [363], while others employed viral vectors to introduce designer receptors into specific spinal cord neuron subsets (e.g., those expressing CCK or PKC) that can be orally activated by a synthetic ligand to block pain signals for potentially 5–10 years [364]. Concurrently, regenerative approaches like platelet‐rich plasma (PRP) and mesenchymal stem cells (MSCs) aim to promote tissue healing. Two 2024 preclinical studies demonstrated that PRP‐derived exosomes boosted bone marrow MSCs which synergistically promoted peripheral nerve regeneration [365, 366].

Finally, noninvasive neuromodulation techniques, including transcranial magnetic stimulation (TMS) and transcranial direct current stimulation (tDCS), are being investigated for broader chronic pain applications by targeting cortical areas like the primary motor cortex or dorsolateral PFC to modulate excitability and potentially reverse maladaptive neuroplasticity associated with chronic pain [367]. A 2025 umbrella review of meta‐analyses noted that while excitatory protocols like high‐frequency TMS and anodal tDCS have shown promise in reducing neuropathic pain intensity, the overall quality of evidence remains low due to heterogeneity, underscoring their experimental status [367].

Taken together, effective management of nociceptive pain requires a patient‐centered, hierarchical strategy. Treatment initiation with first‐line analgesics (paracetamol, NSAIDs) is standard, escalating to second‐line agents (including cautious opioid or cannabinoid use) and interventional procedures (from injections to advanced neuromodulation like SCS) for refractory cases. Crucially, psychological approaches should be introduced early in the course of chronic pain to address its multidimensional impact. The future of nociceptive pain therapy lies in the refinement of targeted biological agents and the personalized integration of pharmacological, interventional, and behavioral modalities based on individual pain mechanisms and psychosocial contexts.

### 6.2 Neuropathic Pain

The management of neuropathic pain, resulting from a lesion or disease of the somatosensory nervous system, is guided by international guidelines and follows a structured, mechanism‐informed approach. Treatment is often challenging due to the complexity of underlying pathophysiology, and a multimodal strategy is typically required [368]. This framework outlines the progression from foundational pharmacotherapy to advanced interventions, with the overarching goals of alleviating suffering and improving organ functionality [368].

#### 6.2.1 First‐Line Pharmacological Options

The management of neuropathic pain differs fundamentally from that of nociceptive pain, relying primarily on antidepressant and anticonvulsant medications as first‐line options with the overarching goals being alleviated suffering and improved organ functionality [368]. First‐line agents include gabapentinoids, tricyclic antidepressants (TCAs), and SNRIs, all of which modulate neuropathic signaling through two principal mechanisms, i.e., (1) inhibiting the reuptake of 5‐HT and norepinephrine to enhance descending inhibitory pathways, and (2) potentiating GABAergic inhibition in the spinal cord [336, 369].

Gabapentinoids, a cornerstone of neuropathic pain treatment, act by binding to the α2δ subunit of voltage‐gated calcium channels, thereby reducing neuronal hyperexcitability and attenuating the release of excitatory neurotransmitters [370]. This mechanism underlies their efficacy in conditions such as diabetic neuropathy, PHN, and spinal cord injury‐related pain. As anticonvulsant GABA analogs, both gabapentin and pregabalin promote the internalization of presynaptic calcium channels, disrupt calcium‐mediated signaling, and ultimately decrease synaptic excitation, leading to suppressed abnormal pain signal transmission [369, 371, 372]. Gabapentin, with a half‐life of 5–7 h, is typically administered in divided daily doses up to 1800 mg. Extended‐release formulations such as GRALISE and the prodrug gabapentin enacarbil (HORIZANT) offer improved tolerability and once‐daily dosing. Pregabalin, which features more predictable pharmacokinetics and a faster onset of action, is commonly prescribed at 300–600 mg daily and is also available in a controlled‐release formulation (Lyrica CR) [370]. The efficacy of both drugs in treating PHN and diabetic neuropathy has been validated in randomized controlled trials and approved by the FDA [370]. However, immediate‐release gabapentinoids are associated with notable adverse effects including sedation, dizziness, fatigue, weight gain, cognitive impairment, depression, suicidal ideation, and potential risks of misuse, abuse, and respiratory depression [370]. Agent selection should therefore be individualized based on comorbidities and prior treatment responses [45, 370], and combination therapy may be considered when monotherapy yields insufficient pain relief [370].

TCAs, such as amitriptyline and nortriptyline, represent another foundational first‐line pharmacological class for neuropathic pain. Their primary mechanism of action involves the potent inhibition of presynaptic reuptake pumps for both 5‐HT and norepinephrine within the CNS. This blockade leads to increased synaptic concentrations of these monoamines, which in turn enhances the activity of descending inhibitory pathways originating from the brainstem, particularly the RVM. This enhanced descending noradrenergic and serotonergic tone acts on α2‐adrenergic and 5‐HT receptors in the spinal dorsal horn to suppress the transmission of nociceptive signals from primary afferent neurons to second‐order projection neurons. Beyond this monoaminergic effect, TCAs exert secondary analgesic properties through additional mechanisms, including sodium channel blockade, which stabilizes hyperexcitable neuronal membranes, and antagonism of NMDA receptors, which modulates central sensitization. Clinically, TCAs have demonstrated robust efficacy in various neuropathic pain conditions, most notably PHN and painful diabetic neuropathy. Treatment is typically initiated at very low nocturnal doses (e.g., 10–25 mg of amitriptyline) to capitalize on their sedative side effects for improving comorbid sleep disturbance, with gradual upward titration based on analgesic response and tolerability. However, their use is significantly constrained by a broad profile of anticholinergic side effects (e.g., dry mouth, constipation, urinary retention, blurred vision), antihistaminergic effects (sedation), and anti‐α1‐adrenergic effects (orthostatic hypotension). Furthermore, their quinidine‐like effect on cardiac conduction, which can prolong the QTc interval, necessitates careful cardiovascular screening and often precludes their use in elderly patients or those with pre‐existing heart disease, frequently positioning them as a secondary choice after SNRIs or gabapentinoids [45, 336, 368, 369].

SNRIs, including duloxetine and venlafaxine, are equally positioned as first‐line pharmacological agents, offering a potentially more favorable tolerability profile compared with TCAs. Like TCAs, their core mechanism hinges on the dual inhibition of 5‐HT and norepinephrine reuptake, thereby amplifying the activity of the brainstem‐spinal descending pain inhibitory system. This action is particularly crucial for attenuating the central amplification of pain signals characteristic of neuropathic states. Duloxetine has garnered the strongest evidence base and regulatory approval for the management of painful diabetic peripheral neuropathy and fibromyalgia (which often features a neuropathic pain component). Its efficacy is attributed to this dual reuptake inhibition, with a higher relative potency for 5‐HT at lower doses and a more balanced effect on both monoamines at therapeutic analgesic doses (typically 60 mg daily). Venlafaxine, while also effective, requires dosing at higher levels (typically ≥150 mg/day) to engage meaningful norepinephrine reuptake inhibition for analgesic purposes. The primary advantages of SNRIs over TCAs include a markedly reduced burden of anticholinergic and antihistaminergic side effects, as well as a lower risk of cardiac toxicity, making them suitable for a broader patient population, including many older adults. Common side effects are instead related to their serotonergic and noradrenergic activity and may include nausea, insomnia, headache, sweating, and a modest increase in blood pressure (particularly with venlafaxine), necessitating routine monitoring. The clinical decision between an SNRI and a gabapentinoid often depends on comorbidity profiles; SNRIs are frequently preferred in patients with coexisting depression or anxiety, while gabapentinoids might be chosen for those with prominent sleep initiation problems or anxiety where sedation is desired. Both classes require slow titration from a low starting dose to manage initial side effects and optimize long‐term adherence [45, 336, 368, 369].

#### 6.2.2 Second‐Line Therapies

For patients with inadequate response to first‐line treatments, second‐line therapies including topical agents, opioids and botulinum toxin type A (BoNT‐A) offer important alternatives.

Among these options, topical agents constitute a notable category, including lidocaine and capsaicin. While 5% lidocaine patches act by blocking sodium channels to reduce peripheral nerve firing, 8% capsaicin patches induce nociceptor defunctionalization through sustained activation and subsequent desensitization of the TRPV1 receptor [370]. Capsaicin creams, applied at intervals such as every 3 months, have also demonstrated efficacy in conditions like diabetic neuropathy.

Opioids such as tramadol and tapentadol are also classified as second‐line agents due to risks of developing dependence, respiratory depression, and potential abuse [336, 373]. Their limited application is further warranted by several neuroadaptive changes in chronic pain states, including downregulation of opioid receptors, increased Aβ‐fiber‐mediated allodynia, CCK‐mediated antagonism of opioid signaling, and NMDA receptor‐mediated hyperexcitability in the dorsal horn, all of which contribute to reduced opioid efficacy and elevated analgesic thresholds [374, 375]. These agents are occasionally preferred in refractory neuropathic pain due to their dual mechanisms, that is, μ‐opioid receptor agonism coupled with monoamine reuptake inhibition (5‐HT and norepinephrine), which may provide broader symptomatic relief [41, 370].

BoNT‐A provides a targeted peripheral intervention via proteolytic cleavage of SNAP‐25, a SNARE protein required for synaptic vesicle fusion. This inhibits the release of pain mediators such as glutamate, substance P, and CGRP from nociceptors, thereby reducing peripheral sensitization and potentially attenuating central amplification [376]. Administered via localized subcutaneous or intradermal injections, BoNT‐A has demonstrated its efficacy in treating conditions including PHN, diabetic neuropathy, and trigeminal neuralgia [376]. Although adverse effects such as injection site pain, erythema, and mild muscle weakness are typically transient and localized [377], its use is limited by the need for specialized training, repeated administrations, and considerable cost [368].

#### 6.2.3 Third‐Line and Adjunct Therapies

Third‐line treatments for neuropathic pain are reserved for patients who remain unresponsive to or intolerant of first‐ and second‐line therapies. These options, primarily including NMDA receptor antagonists and cannabinoids, often have more limited evidence, narrower mechanisms of action, or higher side effect burdens. Nevertheless, they can offer meaningful symptom relief in carefully selected patients through distinct neuromodulatory pathways.

NMDA receptor antagonists, such as ketamine and dextromethorphan, target central sensitization mediated by glutamate‐driven calcium influx and synaptic hyperexcitability [375]. Intravenous ketamine administered in sub‐anesthetic doses (e.g., 75–475 mg) under close monitoring can provide rapid though often short‐lived (less than 1 month) analgesia in conditions like complex regional pain syndrome or diabetic neuropathy [378]. However, its use is limited by risks of psychotomimetic effects, hypertension, hepatotoxicity, and sedation [379]. Dextromethorphan, an oral alternative with additional activity at sigma‐1 and nicotinic receptors, has shown antiallodynic and antihyperalgesic effects in preclinical models. Clinically, its utility is constrained by unpredictable bioavailability, variable response, and side effects such as drowsiness and dizziness [379]. It is most often used adjunctively with gabapentin or opioids to enhance antiallodynic outcomes [368].

Cannabinoids, including both plant‐derived and synthetic formulations, represent another third‐line option. Meta‐analyses suggest a modest analgesic effect, with an average reduction of 0.67 points on a 10‐point pain scale compared with placebo [345]. Although side effects such as dry mouth, fatigue, and cognitive changes are usually mild and transient [345], cannabinoids are generally reserved for refractory cases due to their psychoactive potential and variable efficacy [368].

#### 6.2.4 Interventional Procedures

For patients with severe neuropathic pain refractory to first‐ through third‐line pharmacological therapies, a hierarchy of interventional and surgical options may be considered, progressing from less invasive diagnostic/therapeutic procedures to definitive implantable neuromodulation. Initial interventions for well localized or sympathetically maintained pain include epidural steroid injections or selective nerve root blocks for radicular pain (e.g., sciatica), and sympathetic nerve blocks (e.g., stellate ganglion, lumbar sympathetic) for conditions like complex regional pain syndrome, which can provide both diagnostic information and therapeutic relief [41]. For refractory cases, the definitive interventional treatment is advanced neuromodulation. This includes SCS, which involves the surgical implantation of electrodes in the epidural space to deliver electrical pulses to the dorsal columns, masking pain signals through mechanisms such as enhanced GABAergic inhibition [368, 370], and peripheral nerve stimulation (PNS), which involves implanting electrodes along specific peripheral nerves [41, 45]. A more targeted form of neuromodulation, DRG stimulation, offers precise targeting for focal limb pain. For patients seeking a noninvasive alternative, transcutaneous electrical nerve stimulation (TENS) applies low‐voltage electrical currents through surface electrodes to modulate superficial nerve activity [41, 45]. Finally, in carefully selected cases where a clear anatomic nerve compression is identified (e.g., in some entrapment neuropathies), surgical decompression may be a viable option to address the underlying structural pathology [41].

#### 6.2.5 Psychological Approaches

Given the high comorbidity of mood disorders and pain‐related disability, psychological therapies are fundamental. CBT is the cornerstone, helping patients restructure catastrophic thinking and reduce fear‐avoidance behaviors. MBSR and ACT focus on nonjudgmental acceptance of pain and engagement in valued activities. These approaches, along with pain reprocessing therapy and structured physical exercise, do not abolish pain but significantly reduce suffering, improve function, and enhance the effectiveness of other treatments [370].

#### 6.2.6 Experimental Treatments

Experimental treatments for neuropathic pain have been pursuing highly targeted mechanisms to address the complex pathophysiology, with significant focus on selective ion channel modulation, where sodium channel blockers targeting the pain‐specific isoforms Nav1.7 and Nav1.8 aim to precisely inhibit hyperexcitable nociceptors without disrupting motor or autonomic function [380, 381, 382]. As mentioned before, a critical limitation of these strategies lies in the degeneracy of dominant nociceptor subtypes that may dynamically shift following nerve injury or over time, diminishing the efficacy of highly selective monotherapies [361, 362].

In the realm of gene therapy, innovative approaches are in early clinical and advanced preclinical stages, such as using viral vectors (e.g., adeno‐associated viruses) to deliver genes for endogenous opioid peptides like enkephalin or endorphin precursors directly to DRG or the spinal cord dorsal horn, promoting sustained, local analgesic production [383, 384]. Complementary strategies involve using CRISPR‐based systems or small interfering RNA (siRNA) to silence the expression of pain‐promoting genes, such as those encoding sodium channels (e.g., SCN9A for Nav1.7) or proinflammatory mediators, with preclinical models of neuropathic pain demonstrating long‐term pain relief from a single intervention [385].

Immunomodulation represents another frontier, shifting the focus from neurons to the neuroimmune interface, with therapies actively investigating monoclonal antibodies and small molecules to neutralize key proinflammatory cytokines like TNF‐α and IL‐6, which are elevated in conditions such as painful diabetic neuropathy and PHN and drive both peripheral and central sensitization [386, 387]. Concurrently, there is a growing interest in targeting activated spinal microglia and astrocytes, the CNS's resident immune cells, using inhibitors of specific signaling pathways such as the purinergic 2×7 (P2×7) receptor or p38‐MAPK to suppress the release of neuroexcitatory and proinflammatory substances that maintain neuropathic pain states [388, 389].

Novel neuromodulation technologies are evolving beyond traditional open‐loop systems, with closed‐loop SCS systems representing a significant advance. These devices use real‐time algorithms to analyze recorded neural signals (e.g., evoked compound action potentials) and automatically adjust stimulation parameters in response to the patient's position and activity, aiming to provide more consistent and personalized pain relief while potentially reducing energy use and paresthesia, as evidenced by emerging clinical trial data [390]. Meanwhile, noninvasive TMS, particularly TMS targeting the primary motor cortex (M1) or dorsolateral PFC, is being rigorously studied for its ability to induce neuroplastic changes that modulate the pain matrix, with systematic reviews and meta‐analyses indicating it can produce statistically significant, though often modest, reductions in neuropathic pain intensity, positioning it as a promising adjunctive or alternative therapy for refractory cases [391, 392, 393, 394, 395].

Collectively, neuropathic pain management requires a sequential, patient‐tailored strategy. First‐line treatment with gabapentinoids, TCAs, or SNRIs forms the foundation. Second‐line topical agents, opioids, and BoNT‐A offer alternatives. Third‐line options like NMDA receptor antagonists or cannabinoids are reserved for refractory cases. For severe, drug‐resistant pain, interventional procedures, particularly advanced neuromodulation, provide powerful options. Psychological support and physical modalities should be integrated throughout. The future lies in more precise, disease‐modifying therapies targeting specific molecular pathways and neural circuits.

### 6.3 Nociplastic Pain

The management of nociplastic pain, a condition characterized by altered nociception and CNS sensitization without clear tissue or nerve damage, demands a distinct and comprehensive strategy. Unlike nociceptive or neuropathic pain, pharmacotherapy often yields modest effects and significant adverse effects [46, 396, 397, 398], necessitating a primary focus on nonpharmacological, mechanism‐based interventions that target central dysregulation, dysfunctional pain processing, and associated psychosocial comorbidities [46, 264, 398, 399]. The therapeutic approach is inherently multimodal and patient centered.

#### 6.3.1 First‐Line Pharmacological Options

The foundational management of nociplastic pain conditions, such as fibromyalgia and chronic widespread pain, is decisively nonpharmacological. This approach centers on active patient engagement through structured education and prescribed physical activity, forming the essential dual pillars of first‐line care.

Patient education constitutes the indispensable cornerstone of this strategy. Its primary objective is to empower individuals by demystifying their condition within a clear biopsychosocial framework. Effective education provides a scientifically grounded explanation of central sensitization, which validates the patient's often‐disputed experience, reduces catastrophic fear associated with pain, and shifts the perspective from one of structural damage to one of functional nervous system dysregulation [46, 400, 401]. This educational process extends beyond conceptual understanding to deliver practical self‐management skills. Patients are taught critical techniques for stress management, optimized sleep hygiene, and activity pacing. Pacing, in particular, is a key behavioral intervention that teaches individuals to balance activity and rest to avoid the debilitating “boom‐bust” cycles that perpetuate pain and disability, thereby promoting sustainable increases in function [46, 400, 401].

Concurrently, prescribed aerobic and mind–body exercise forms the second critical pillar, representing one of the most evidence‐supported interventions for reducing pain severity and improving physical function [402]. The prescription emphasizes graded, low‐impact aerobic activities such as walking, swimming, or stationary cycling, which are initiated at a tolerable level and progressively increased. Mindful movement practices like Tai Chi and Yoga are equally endorsed, as they uniquely integrate gentle physical movement with breath regulation, meditative focus, and enhanced interoceptive awareness. The therapeutic benefits of these exercises are multifactorial. Physiologically, regular participation strengthens descending inhibitory pain pathways, increases parasympathetic nervous system tone (promoting relaxation), and can reduce markers of systemic inflammation. Behaviorally, they directly counteract fear‐avoidance and build self‐efficacy. Collectively, these effects work to reduce widespread hyperalgesia and support adaptive neuroplasticity, effectively “recalibrating” the hypersensitive CNS [402]. It is important to note, however, that while essential, physical activity alone may yield constrained benefits for individuals with moderate to severe symptoms, who typically require integration within a broader multimodal treatment plan [403].

CBT for pain (CBT‐P) is positioned with equal importance as a first‐line psychological intervention. CBT‐P is a structured, goal‐oriented psychotherapy predicated on the understanding that cognitive appraisals and behavioral responses profoundly shape the pain experience and functional outcomes. The therapy involves cognitive restructuring to identify and challenge maladaptive, catastrophic thoughts about pain, and behavioral activation to gradually and systematically re‐engage patients in avoided but valued activities. A standard CBT‐P protocol integrates pain neuroscience education, relaxation training, activity pacing, attentional diversion techniques, and sleep hygiene instruction. By enhancing self‐efficacy and reducing pervasive fear‐avoidance behaviors, CBT‐P successfully decreases the perceived threat and burden of pain, leading to significant improvements in daily function and quality of life [46]. Beyond traditional CBT‐P, other validated psychological modalities play a crucial role. ACT fosters psychological flexibility, teaching patients to accept unavoidable pain sensations while committing to actions aligned with their personal values. MBSR cultivates a stance of nonreactive, present‐moment awareness toward sensory and emotional experiences, thereby reducing the emotional suffering and reactivity that amplify pain [46]. For a subset of patients, particularly those with a history of underlying trauma, modalities such as emotional awareness and exposure therapy or neural reprocessing techniques may offer additional benefit by addressing the psychological drivers that can maintain a sensitized state [46, 398, 404].

#### 6.3.2 Second‐Line Therapies

The management of nociplastic pain prioritizes nonpharmacological strategies, positioning pharmacological agents as second‐line or adjunctive treatments aimed at symptom modulation rather than cure [402]. Antidepressants form the pharmacological cornerstone, with SNRIs such as duloxetine and milnacipran possessing the most robust evidence for efficacy. Their primary mechanism involves enhancing descending inhibitory pathways from the brainstem to the spinal cord. TCAs, notably amitriptyline, are typically used at low doses, primarily leveraging their sedative and neuromodulatory properties to improve sleep quality and indirectly attenuate central sensitization. Anticonvulsants or gabapentinoids, including pregabalin and gabapentin, may provide benefit by attenuating neuronal hyperexcitability through modulation of voltage‐gated calcium channels, although their therapeutic effects in nociplastic conditions are frequently modest and inconsistent. In contrast, conventional analgesics such as selective COX‐2 inhibitors and acetaminophen demonstrate negligible efficacy against the core central sensitization pathophysiology. Critically, opioids are contraindicated in the long‐term management of nociplastic pain due to a profound lack of analgesic efficacy, coupled with a high risk of inducing opioid‐induced hyperalgesia, physical dependence, respiratory depression, and overdose [402].

Physical modalities such as acupuncture, acupressure, and chiropractic manipulation have been frequently incorporated into a multimodal treatment plan as adjunctive therapies, though their evidence base and proposed mechanisms of action differ considerably [404]. Acupuncture, involving the insertion of fine needles at specific anatomical sites, is postulated to alleviate pain through a multifactorial mechanism. This includes the activation of Aδ‐ and C‐fibers, which triggers the release of endogenous opioids (e.g., endorphins, enkephalins) and monoamines (5‐HT, norepinephrine) within the CNS. Additionally, functional neuroimaging studies suggest acupuncture can modulate activity in limbic brain regions, such as the ACC and amygdala, which are critically involved in the affective and emotional dimensions of pain processing [405, 406, 407]. These combined actions are believed to collectively reduce central sensitization. Beyond neuromodulation, emerging translational evidence points to a role for acupuncture in restoring systemic oxidative homeostasis. A prospective clinical study involving female fibromyalgia patients found elevated baseline levels of oxidative stress markers (glutathione species) compared with healthy controls. Following a course of acupuncture treatment, these patients exhibited a significant increase in intracellular antioxidant activity, suggesting a novel mechanism contributing to symptom relief [408].

Acupressure shares the foundational principles of acupuncture, applying manual pressure rather than needles to stimulate specific anatomical points in a noninvasive manner. It is considered a more accessible and patient‐administered option, though high‐quality evidence for its specific efficacy in nociplastic pain requires further substantiation [409, 410]. Chiropractic manipulation, particularly spinal adjustment techniques, seeks to improve spinal joint mobility and biomechanical function. The proposed analgesic mechanism involves reducing aberrant peripheral nociceptive input from spinal structures, thereby potentially diminishing the facilitatory drive to already sensitized central pain pathways [411, 412, 413]. However, the application of high‐velocity, low‐amplitude thrust techniques in this patient population warrants extreme caution. In individuals with established central sensitization, such forceful manipulations carry a significant risk of provoking widespread symptom exacerbation, muscle guarding, and increased pain, highlighting the necessity for gentler, nonthrust approaches [414].

The integration of these diverse physical interventions must occur within a comprehensive, biopsychosocial treatment framework. This approach emphasizes that their utility is not standalone but as components of a personalized plan that simultaneously addresses psychological factors (e.g., through CBT), promotes graded exercise, and improves sleep hygiene. For patients with moderate‐to‐severe nociplastic pain, a unimodal approach focusing solely on physical activity or a single modality is typically insufficient [414]. Effective management requires a coordinated, multimodal strategy that concurrently targets the biological, psychological, and social dimensions of this complex condition, with physical therapies playing a supportive, adjunctive role tailored to individual patient tolerance and response.

#### 6.3.3 Interventional Procedures

For patients with symptoms refractory to conventional pharmacological and physical therapies, neurostimulatory techniques present a promising avenue by modulating neural activity across central, autonomic, and peripheral pathways [415, 416, 417, 418]. The tDCS and TMS target cortical regions such as the primary motor cortex and dorsolateral PFC to normalize cortical excitability and reinforce impaired descending inhibitory control. Vagus nerve stimulation, delivered either invasively or noninvasively via transcutaneous devices, may reduce central sensitization through parasympathetic activation and suppression of proinflammatory neuroimmune signaling. TENS applies low‐intensity electrical currents peripherally to elicit segmental inhibition in the spinal dorsal horn via gate‐control mechanisms, with potential engagement of supraspinal modulatory networks. While the robustness and consistency of evidence for these modalities continue to evolve, they represent an expanding therapeutic arsenal for refractory nociplastic pain.

#### 6.3.4 Experimental Treatments

Experimental treatments for nociplastic pain have been distinctly focused on directly correcting the CNS dysregulation that defines the condition, with noninvasive brain stimulation being a leading investigational area where techniques like TMS [419] and tDCS [420] aim to recalibrate maladaptive cortical excitability. For instance, a meta‐analysis of TMS for fibromyalgia concluded that high‐frequency stimulation (typically 10 Hz) applied to the M1 yielded a significant, though moderate, reduction in pain intensity compared with sham stimulation, with the analgesic effect believed to stem from the induction of neuroplasticity that strengthens descending inhibitory controls, while anodal tDCS applied to the same region has shown promise in modulating pain perception and improving conditioned pain modulation, a key measure of endogenous pain inhibition that is often impaired in nociplastic conditions [419, 420].

The search for novel pharmacological targets is moving beyond traditional neurotransmitter systems to address the underlying neuroimmune and molecular pathology, with significant research into drugs that inhibit the activation of spinal and brain microglia, such as small‐molecule antagonists of the purinergic P2×7 receptor or p38–MAPK inhibitors, which in preclinical models of widespread pain can attenuate the release of proinflammatory cytokines (e.g., IL‐1β, TNF‐α) and reduce pain‐like behaviors [421]. Simultaneously, there is growing interest in refining modulation of the endocannabinoid system beyond plant‐derived cannabinoids, focusing on developing compounds that selectively target the cannabinoid Type 2 (CB2) receptor to potentially dampen neuroinflammation [422] or those that inhibit fatty acid amide hydrolase to boost levels of the endogenous cannabinoid anandamide [423], aiming for analgesic efficacy with a reduced psychoactive burden.

Digital therapeutics and virtual reality (VR) represent a paradigm shift in delivering behavioral interventions, where immersive VR environments are being studied not only for acute pain distraction through engaging, multisensory experiences but also for graded exposure therapy, helping patients with high fear‐avoidance to safely re‐engage with feared movements in a controlled virtual setting, thereby reducing catastrophizing and disability [424, 425, 426, 427]. Concurrently, validated app‐based programs that deliver core components of CBT, mindfulness meditation, and activity pacing are demonstrating efficacy in randomized trials (NCT04523714) for improving pain interference and self‐efficacy in fibromyalgia, with the advantage of increasing accessibility and enabling consistent, personalized self‐management [428, 429].

Finally, research into lifestyle and dietary interventions is examining their foundational role in modulating systemic inflammation and the gut–brain axis. While clinical studies have been investigating specific anti‐inflammatory dietary patterns (e.g., omega‐3 supplementation) for their potential to reduce pain severity and improve quality of life in conditions like IBS [430] and fibromyalgia [431], mind–body practices, such as meditation, yoga, and tai chi that integrate gentle movement with breath regulation and meditative focus, have got support from evidence showing that they can lower circulating inflammatory markers, improve heart rate variability, and reduce widespread hyperalgesia, effectively targeting multiple pillars of the nociplastic pain phenotype through nonpharmacological means [320, 432, 433].

In summary, the management of nociplastic pain represents a paradigm shift from a biomedical to a biopsychosocial model. The foundation is a sustained commitment to first‐line nonpharmacological strategies, that is, comprehensive education, graduated mind–body exercise, and CBT. Pharmacological agents and select physical modalities play a secondary, supportive role. Interventional procedures have minimal indication. Advanced psychological therapies and emerging neurostimulation are central for refractory cases. Future treatments will likely focus on sophisticated neuromodulation and pharmacotherapies that directly target the dysregulated central pain processing pathways characteristic of this complex condition.

Conclusively, the therapeutic landscape for chronic pain is defined by distinct, mechanism‐guided paradigms. Nociceptive pain is managed through a stepwise escalation from simple analgesics to interventional procedures aimed at the peripheral site of injury. Neuropathic pain relies on a foundation of neuromodulating drugs, with interventional neuromodulation serving as a definitive option for drug‐resistant cases. Most distinctively, nociplastic pain management inverts the traditional hierarchy, placing nonpharmacological, brain‐targeting strategies at its core, while relegating pharmacotherapy to a supportive role. A unifying principle across all subtypes is the necessity of a multimodal, patient‐centered approach that integrates pharmacological, interventional, physical, and psychological modalities. The future of pain therapy lies in refining these frameworks through identifying the causative pain source especially for nociplastic pain and establishing multimodal therapeutics for more effective and personalized relief.

## 7 Emerging Concepts: CAP for Therapeutics and Hippocampus for Sensing

This section introduces and critically evaluates two promising conceptual frontiers in chronic pain research and therapy. The first is the application of CAP, an ionized gas generating a regulated flux of reactive oxygen and nitrogen species (RONS), as a novel therapeutic modality. CAP's potential stems from its demonstrated, multimodal biological effects, promoting tissue and nerve healing, modulating comorbidities like cancer and obesity, and interacting with hormonal pathways, which collectively address diverse mechanisms underpinning nociceptive, neuropathic, and nociplastic pain. The second frontier proposes an original neurobiological model, positing the hippocampus as a putative central “sensor” for nociplastic pain. This hypothesis suggests the hippocampus may interface with maladaptive central information processing, analogous to how peripheral nociceptors interface with physical injury, with the hypothalamus serving as a decoding hub. While both concepts are supported by scientific evidence and offer transformative potential to revolutionize diagnostic and therapeutic strategies, their translation from theory to clinic still requires rigorous experimental and clinical validations.

### 7.1 CAP as an Emerging Therapeutics for Chronic Pain

CAP is an innovative technology that generates a regulated flux of RONS at near‐ambient temperatures. This cocktail includes short‐lived reactive species such as hydroxyl radicals (•OH) and superoxide (O_2_•^−^), as well as long‐lived species like singlet oxygen and nitric oxide, hydrogen peroxide, ozone, nitrogen dioxide, and nitric acid [18, 19, 20, 21, 22]. The precise spatiotemporal delivery of this specific RONS profile underpins its therapeutic selectivity [434, 435]. By enabling dosage‐controlled modulation of redox signaling pathways, CAP selectively induced programmed cell death in pathological cells [21, 436, 437, 438, 439] or, conversely, stimulated regenerative and proliferative processes in healthy tissues [440]. This mechanistic precision allows CAP to spare surrounding healthy tissue, offering a targeted and adaptable strategy to harness the biological effects of RONS for diversified therapeutic purposes.

As a result, CAP has demonstrated considerable promise across multiple biomedical domains, including wound healing [441, 442, 443, 444, 445, 446] and microbial disinfection [447, 448, 449]. Its application has been extended to the management of complex pathologies such as cancer [22, 450, 451, 452], psoriasis [453], and neurodegenerative disorders [454]. Although not yet formally adopted in pain management, a growing body of clinical and preclinical evidence have supported its feasibility for treating analgesia through distinct, pathology‐specific mechanistic pathways. It has been indicated CAP can alleviate nociceptive pain by modulating tissue‐level inflammatory and damage healing signaling [455, 456, 457, 458, 459, 460], improve neuropathic pain via targeting neuroinflammatory and neuroregenerative pathways [461, 462, 463, 464, 465, 466, 467], and alleviate cancer‐related pain through the targeted resolution of the tumor comorbidity itself [468, 469]. Furthermore, evidence suggests potential in resolving obesity‐associated pain by modulating inflammatory and metabolic dysregulation [470, 471, 472, 473] and in enhancing treatment efficacy among female patients via interactions with estrogen and growth factor signaling pathways [21, 436, 438, 439, 474, 475]. This spectrum of actions has highlighted CAP's potential as a multimodal therapeutic agent whose effects are dictated by its precise, mechanism‐driven application to specific disease pathophysiology.

#### 7.1.1 Therapeutic Opportunities

##### 7.1.1.1 Modulation of Tissue Inflammatory and Damage Healing Pathways

**FIGURE 5:**
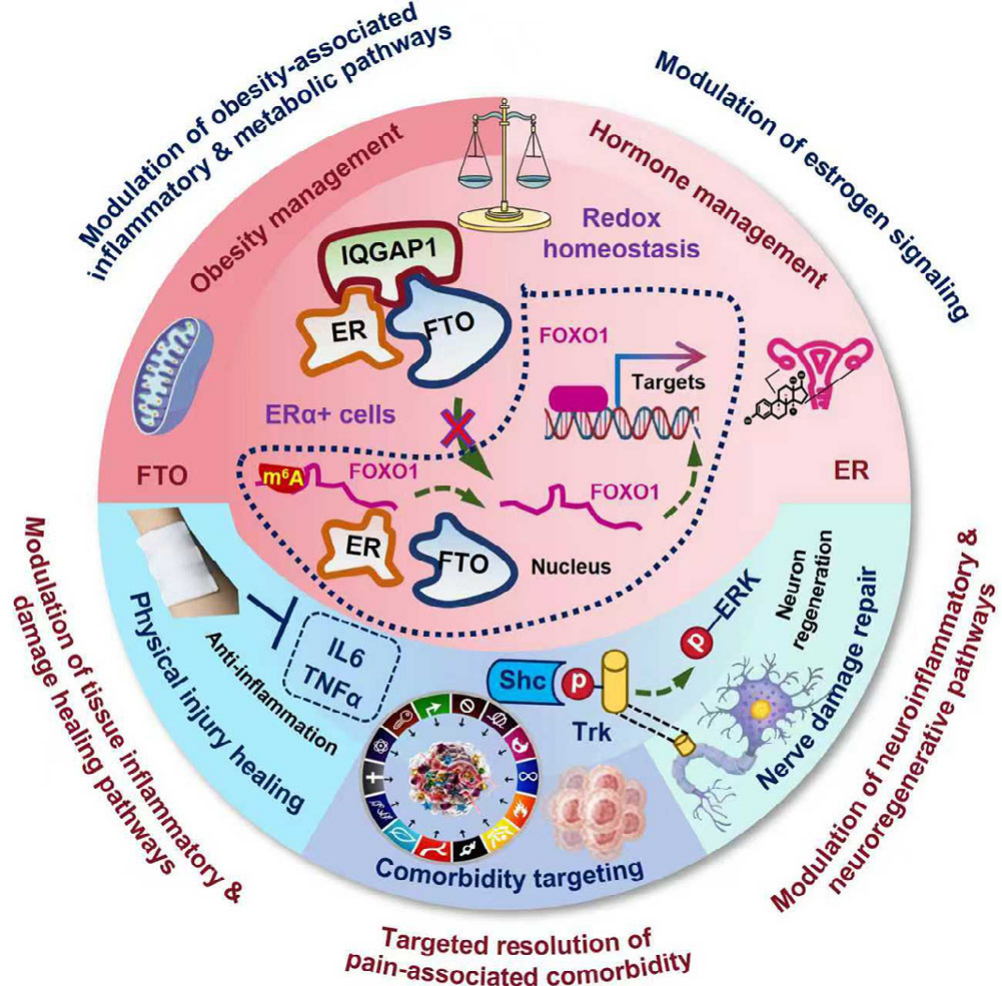
Cold atmospheric plasma as a promising approach for chronic pain relief. Schematic illustration of CAP as a multitarget therapeutic for chronic pain, delineating its distinct, mechanism‐driven actions across diverse pain types and comorbidities. The figure depicts the possible mechanisms that CAP can help treat chronic pain. Key therapeutic opportunities include: (1) modulation of tissue inflammatory and damage healing pathways, where CAP suppresses proinflammatory cytokines like IL6 and TNFα to alleviate nociceptive pain; (2) modulation of neuroinflammatory and neuroregenerative pathways, where CAP activates the Trk/Ras/ERK signaling cascade to promote neuron regeneration, targeting neuropathic pain; (3) targeted resolution of pain‐associated comorbidity, where CAP's antitumor actions (e.g., targeting cancer hallmarks) directly ablate cancer lesions to resolve cancer‐related pain; (4) modulation of obesity‐associated inflammatory and metabolic pathways, where CAP inhibits FTO and dampens systemic inflammation/oxidative stress to mitigate obesity‐driven pain; and (5) modulation of estrogen signaling, where CAP interacts with estrogen receptors (ERs) signaling to influence gender‐biased pain mechanisms. While “physical injury healing,” “nerve damage restoration,” and “comorbidity targeting” (the lower three panels) represent the impact of CAP on the sources of pain through canonical mechanisms, “obesity management” and “estrogen crosstalk” (the two upper panels) implicate the innovative mechanism of action underscoring the efficacy of CAP in resolving chronic pain. Note that the axes of ER and FTO interact, where FTO plays the dominant role in promoting obesity and ER facilitates this process by assisting its entry into the nucleus. CAP can effectively block their interaction, thereby inhibiting the nuclear localization of both FTO and ER. Together, these multifaceted actions may position CAP as a multimodal option targeting all three major types of chronic pain.

While not exhaustively studied for pain relief as a primary endpoint, CAP has demonstrated promising effectiveness in reducing nociceptive pain through specific, mechanism‐driven actions that promote tissue repair and modulate inflammation, particularly in the context of refractory chronic wounds and bacterial infections. This analgesic effect is not a general byproduct but is intrinsically linked to CAP's targeted biological activity on damaged tissue. Specifically, the mechanisms by which CAP achieves pain relief in these contexts are multifaceted and directly address the pathophysiological sources of nociceptive pain. The primary pathways involve the direct suppression of proinflammatory cytokines at the wound site, coupled with the stimulation of cellular proliferation and angiogenesis. By reducing the inflammatory load, a key driver of pain in wounds and infections, and accelerating the tissue repair process, CAP addresses the underlying causes of nociception rather than merely masking the symptom (Figure 5). Substantial clinical evidence has underscored this mechanism of action. In a randomized clinical pilot study of 37 individuals, 12 weeks of once‐weekly CAP treatment significantly reduced pain associated with refractory chronic wounds [455]. Another pilot study involving 10 patients reported that CAP treatment reduced the pain sensation from chronic wounds to one‐tenth of the original score after only four applications [456]. Further supporting this, a case study utilizing the PlasmaDerm VU‐2010 device demonstrated that CAP, as an add‐on therapy, not only promoted complete ulcer healing after 7 weeks but also reduced pain from chronic venous leg ulcers (NCT01415622) [457]. Additionally, a prospective pilot study with 10 participants found that a novel CAP wound dressing significantly outperformed conventional therapy in improving tissue parameters like deep tissue oxygen saturation and, critically, in reducing pain levels at split‐thickness skin graft donor sites [458]. Beyond wounds, CAP's analgesic potential extended to infections, as evidenced by a marked reduction in severe pain for a patient with a complex auditory canal infection following cholesteatoma surgery [459]. The ongoing clinical investigation into CAP's wound‐healing efficacy, which inherently encompasses pain outcomes, further validates its therapeutic role. Completed trials include a multicenter, noninferiority study comparing the Cold Plasma Jet kINPen to best‐practice wound dressings for chronic wound treatment (NCT04965805), and another assessing the Plasma Care device versus placebo (NCT07050667). Currently recruiting trials include a study on plasma therapy for chronic, nonhealing wounds (NCT05855499) and a planned pilot study for chronic venous leg and diabetic foot ulcers (NCT06964048). Collectively, this body of work have demonstrated that CAP's effectiveness against nociceptive pain is grounded in specific, interacting mechanisms, ranging from molecular anti‐inflammatory and proregenerative actions to physical modulation of skin permeability, which collectively resolve the tissue pathology responsible for pain generation.

Furthermore, CAP exhibits a distinct physical mechanism for pain reduction through enhanced transdermal drug delivery, which minimizes procedural injury. For instance, a pretreatment with CAP for 5 min prior to applying topical anesthetic cream significantly decreased pain during subsequent CO_2_ laser procedures for postacne scars. The primary mechanism identified for this analgesic effect was CAP's ability to increase transdermal agent transportation, thereby improving the efficacy of local anesthetics and reducing noxious stimulation (CHiCTR2000029063) [460]. This highlights a precise, technology‐enabled mechanism for pain control.

##### 7.1.1.2 Modulation of Neuroinflammatory and Neuroregenerative Pathways

The acute pain associated with herpes zoster infection is a recognized clinical sign of underlying nerve injury and a precursor to the development of PHN, a chronic neuropathic pain condition [461, 462]. CAP has demonstrated significant potential in alleviating such neuropathic pain through specific biological mechanisms that directly target the pathological processes of nerve damage and inflammation (Figure 5). This represents a targeted therapeutic strategy, moving beyond general analgesia to address the root causes of neuropathic pain. There exist several clinical evidence to support this mechanism. In a prospective randomized placebo‐controlled Phase II study involving 37 herpes zoster patients, a 5‐min CAP treatment effectively relieved acute pain [463]. Similarly, a randomized, parallel, positive‐controlled, noninferiority multicenter clinical trial (ChiCTR2300069993) demonstrated that CAP achieved comparable results to helium–neon (He–Ne) laser therapy in promoting acute pain relief and wound healing in patients with herpes zoster infection [464]. Furthermore, a translational study in domestic cats showed that combining CAP with a multimodal pharmacologic regimen (ketamine, gabapentin, amitriptyline, and prednisone) led to significant improvement in both chronically infected skin lesions and postsurgical neuropathic pain [465]. These outcomes have suggested that CAP's effect is integral to a comprehensive neuropathic pain management strategy.

**FIGURE 6:**
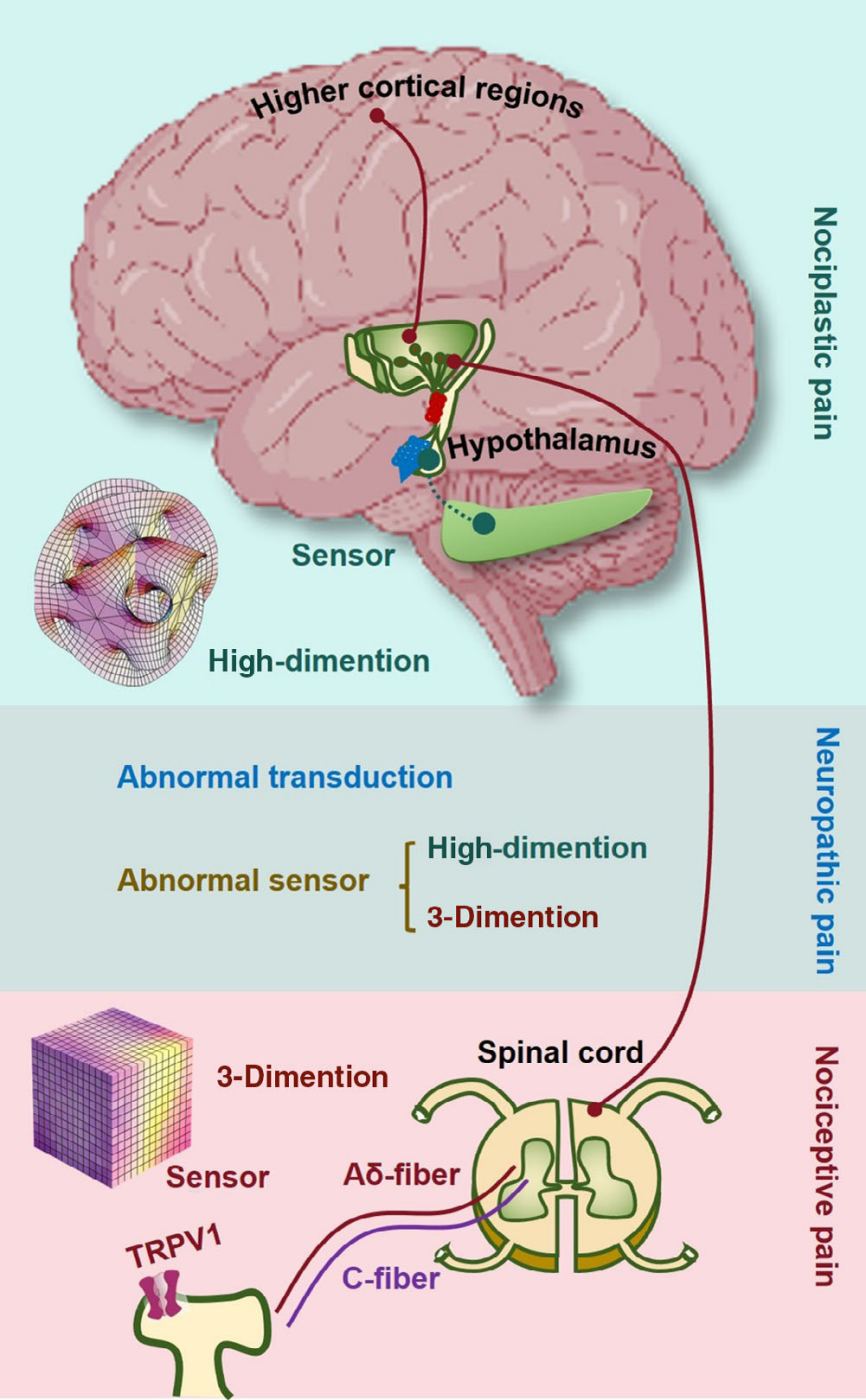
Hippocampus as a sensor of nociplastic pain. In this proposed pain‐sensing model, peripheral nociceptors act as sensors for nociceptive pain by detecting tissue injury, while neuropathic pain involves aberrant signaling through Aδ‐ and C‐fibers due to nerve damage within the somatosensory system. In contrast, the hippocampus is proposed to function as a central sensor for nociplastic pain, receiving information from putative higher‐dimensional fields, beyond conventional three‐dimensional bodily structures, thereby contributing to pain perception in the absence of tissue injury or neural lesions detectable by our three‐dimensional body device. All three types of pain signals converge within the hypothalamus, where they are integrated into coherent interoceptive‐affective representations before being relayed to higher‐order cortical regions for conscious experience. This framework encourages future studies to identify biomarkers (molecular or genetic) of hippocampal dysfunction for improved diagnosis of nociplastic pain and to develop targeted interventions modulating the hippocampal–hypothalamic circuit for more precise treatment strategies. Accordingly, the three types of pain can be reorganized into two large categories, that is, “abnormal sensor” and “abnormal transduction.” That is, both nociceptive and nociplastic pain can be understood as conditions arising from dysfunctional sensory mechanisms. While nociceptors act as receivers encoding information from physical three‐dimensional space, hippocampus may serve a analogous role in connecting us with higher‐dimensional, metaphysically oriented experiences. In contrast, neuropathic pain can be viewed primarily as a pathology of abnormal signal transduction within the nervous system. Building on this framework, pain can be reclassified into two broad categories, that is, “abnormal sensing” and “abnormal transmission.” While nociceptive and nociplastic pain both fall under the first category reflecting dysfunction in sensory encoding structures, neuropathic pain constitutes a distinct class defined by faulty neural signal conduction along pain pathways. Nociceptive and nociplastic pain differ fundamentally in the origin of the signals they process. While nociceptive pain arises from stimuli detected in the physical three‐dimensional environment by nociceptors, nociplastic pain may involve the hippocampus as a receiver of percepts situated in higher‐dimensional, metaphysically framed experiences.

The therapeutic efficacy is underpinned by direct and specific actions on neural tissue (Figure 6). Crucially, CAP has been shown to stimulate neuron regeneration in vitro, proposing its feasibility for treating neurological disorders [466, 467, 476]. More specifically, it has been elucidated that CAP enhanced neural cell differentiation into functional neurons through the precise activation of the Trk/Ras/ERK signaling cascade, a pathway critically involved in neuronal growth and survival. This effect has been validated both in vitro and in vivo, highlighting CAP's potential for treating traumatic injuries of the CNS [466, 467, 476]. These findings have directly implicated that CAP contributes to pain relief not merely through a general anti‐inflammatory effect, but through a targeted proregenerative mechanism that promotes the repair of injured nerves, thereby addressing a fundamental source of neuropathic pain. This capacity to modulate specific cellular differentiation pathways has distinguished CAP's mechanism from nonspecific physical therapies.

##### 7.1.1.3 Targeted Resolution of Pain‐Associated Comorbidity

CAP has demonstrated significant potential for the targeted resolution of cancer‐related pain by directly addressing the underlying tumor pathology, a complex comorbidity that drives nociception [468, 469]. This approach moves beyond symptomatic pain management to modulate the pain source itself (Figure 5). In a retrospective review of 12 patients with advanced head and neck squamous cell carcinoma, CAP treatment was associated with reduced demand for pain medication, diminished fetid odor related to microbial load, and superficial partial tumor remission [469]. Similarly, in another study using the kINPen MED plasma source (Greifswald, Germany), six patients with locally advanced (pT4) oropharyngeal squamous cell carcinoma and infected open ulcerations received CAP applications three times per week at a dosage of 1 min/cm^2^ spaced at 1‐week intervals. The results showed a noticeable reduction in odor, decreased use of pain medication, tumor remission, and a desmoplastic reaction in connective tissue [468]. These clinical outcomes suggest that CAP‐mediated tumor reduction and tissue normalization directly contribute to pain alleviation.

The foundation for this therapeutic effect lies in CAP's established and multifaceted antitumor efficacy, supported by extensive preclinical and clinical evidence indicating it as a promising approach for selective cancer ablation [22, 450, 451, 452]. Preclinically, the anticancer mechanisms of CAP are highly specific and multipronged, directly contributing to the ablation of pain‐generating tumors. These mechanisms include attenuating cancer stemness [22], halting metastasis [477], and potentiating various programmed cell death pathways such as apoptosis [438], ferroptosis [21], and critically, immunogenic cell death (ICD) [478]. The induction of ICD is particularly relevant as it stimulates immune cells involved in innate immunity, such as macrophages [475, 479] and dendritic cells [480, 481], and subsequently activates the adaptive immune response [482]. This immunomodulatory effect can enhance systemic antitumor responses, further contributing to tumor control and the resolution of associated comorbidities like pain.

Clinically, the translation of these mechanisms is underway. The first Phase I clinical trial examining the efficacy and safety of CAP as an onco‐therapy (NCT04267575) was conducted in the United States from March 2020 to April 2021. The study demonstrated a 75% local nonrecurrence rate at a 28‐month follow‐up with no adverse events reported among the 20 recruited patients with metastatic or recurrent solid tumors, underscoring its safety and potential durability. Several other trials are further evaluating CAP's role. A completed trial sponsored by the University Hospital Tuebingen assessed the feasibility of using physical CAP for treating cervical intraepithelial neoplasia, with pathological remission after 3–6 months as the primary endpoint (NCT03218436). Among ongoing trials, a study by the University Hospital Tübingen is recruiting to evaluate CAP for cervical HPV infections (NCT06291311), while the University Hospital Ghent is investigating CAP's effects in patients with peritoneal metastases (NCT06796634). Additionally, a trial sponsored by Universitätsklinikum Hamburg‐Eppendorf is enrolling to examine CAP for treating precancerous duodenal polyps (NCT06435533). Collectively, these studies highlight the growing clinical focus on utilizing CAP's precisely targeted antineoplastic actions to ablate tumors and, by extension, resolve associated morbidities including cancer‐related pain.

##### 7.1.1.4 Modulation of Obesity‐Associated Inflammatory and Metabolic Pathways

The contribution of obesity to chronic pain, particularly neuropathic and nociplastic types, is mediated through a state of persistent, low‐grade systemic inflammation. Adipose tissue in obesity functions as an active endocrine organ, releasing excessive proinflammatory cytokines (e.g., TNF‐α, IL‐6). This adipokine dysregulation stimulates hepatic production of acute‐phase proteins like CRP, perpetuating a inflammatory milieu that exacerbates peripheral and central sensitization pathways involved in pain disorders [483, 484]. CAP has emerged as a potential modulator of these interconnected metabolic and inflammatory circuits (Figure 5). A key proposed mechanism involves the direct targeting of FTO, a critical regulator of RNA methylation and metabolic homeostasis. Preclinical evidence identified CAP as an innovative inhibitor of FTO activity both in vitro and in vivo, suggesting its utility in mitigating obesity and related metabolic syndromes [470]. This is substantiated by preclinical findings in plasma oncology, where CAP downregulated FTO transcription in triple‐negative breast cancer cells, underscoring a mechanistic link between metabolic regulation and oncological effects that might be extended to pain modulation [471].

Beyond FTO inhibition, CAP exerts broader modulatory effects on the metabolic dysfunction characteristic of obesity. It has been shown to improve glycemic control by reducing levels of glycated proteins and mitigating systemic oxidative stress [472, 473]. Furthermore, CAP treatment decreased circulating proinflammatory cytokines, including TNF‐α, IL‐1, and IL‐6, while concurrently restoring the activity of key endogenous antioxidant enzymes such as superoxide dismutase, catalase, and glutathione peroxidase in experimental and animal models [472, 473]. These actions collectively help rebalance redox homeostasis and quench the chronic inflammatory drive. Therefore, CAP may alleviate obesity‐associated pain through a multimodal pharmacological strategy. That is, it may take actions primarily by inhibiting FTO to correct upstream metabolic dysregulation, and concomitantly by dampening the downstream inflammatory cascade and oxidative stress that directly fuel nociceptive and neuropathic signaling pathways. This has positioned CAP not merely as a generic anti‐inflammatory agent, but as a targeted intervention capable of addressing specific molecular hubs within the complex pathophysiology linking obesity to chronic pain.

##### 7.1.1.5 Modulation of Estrogen and Growth Factor Signaling

The well‐documented gender disparity in the prevalence of chronic pain disorders strongly implicates sex hormones, particularly estrogen, as key modulators of nociceptive signaling. Beyond epidemiological associations, extensive preclinical evidence confirms that estrogen significantly influences the excitability of both peripheral and central sensory neurons [485]. For instance, ovariectomized rodents developed mechanical and thermal hyperalgesia, a state that was reversible upon estrogen supplementation [486, 487]. Conversely, administration of 17‐β estradiol has been shown to alleviate neuropathic pain in male rats following nerve injury [488]. The ubiquity of estrogen receptors expressed on nociceptive neurons provides a direct anatomical substrate for these effects, although estrogen's actions can also occur via nongenomic, ER‐independent pathways [489, 490, 491].

Emerging evidence suggests that CAP may interact with these hormonal pain‐regulatory systems. A compelling line of inquiry arises from oncology research, where CAP demonstrates differential cytotoxicity based on cellular ER status. While effective against both ER‐positive and ER‐negative breast cancer cells, preclinical studies consistently reported that triple‐negative (ER‐negative) cells required a lower effective dose of CAP compared with luminal (ER‐positive) subtypes in vitro and in vivo [21, 436, 438, 439, 475]. This dose‐dependent disparity implies an intrinsic biological interplay between CAP and ER signaling pathways. More direct mechanistic insight came from in vitro studies showing that CAP treatment could alter the subcellular distribution of ERα in VERO‐E6 and human mammary epithelial MCF10A cells [474]. Given that ERs dynamically shuttled between the membrane, cytoplasm, and nucleus to orchestrate both rapid nongenomic and long‐term genomic signaling cascades using preclinical models [492], CAP‐induced modulation of ERα localization represents a plausible and specific mechanism to influence estrogen‐sensitive nociceptive circuits (Figure 5).

The signaling crosstalk extends beyond classical hormonal pathways to include growth factor receptors, notably the epidermal growth factor receptor (EGFR). In vitro evidence indicated that extracellular signals like EGF can transactivate ERs [490, 493], highlighting the interconnectedness of these signaling networks. Our previous preclinical work has demonstrated that CAP could precisely modulate EGFR activation, sensitizing distinct phosphorylation sites to different programmed cell death pathways, that is, phosphorylation at Y992/1173 was associated with apoptosis, while Y1068 phosphorylation was linked to ferroptosis [21, 438]. This precise, site‐specific modulation of EGFR signaling by CAP suggests it may function as a paralogous modulator to estrogen, capable of fine‐tuning complex receptor tyrosine kinase–hormone receptor interactions that are implicated in both pain perception and tissue pathology.

Furthermore, the intersection of gender, obesity, and pain suggests a convergent pathophysiological node. The higher prevalence of obesity among women [494] may be partially driven by hormonal influences, as experimental data showed estrogen can promote the nuclear localization of FTO in vitro, thereby contributing to adipogenesis [495] (Figure 5). As previously discussed, CAP exhibited inhibitory activity against FTO [470]. Therefore, a multifaceted mechanistic model emerges. That is, CAP may alleviate pain not only through direct interaction with ER signaling and crosstalk with EGFR but also indirectly by targeting FTO, a downstream effector in the estrogen–metabolic axis. This positions CAP's analgesic potential within a sophisticated framework of interlocking pathways, where its effects on hormonal receptors, growth factor signaling, and metabolic regulators converge to modulate the complex biological underpinnings of gender‐biased and obesity‐related chronic pain.

#### 7.1.2 Translational Challenges

Despite the aforementioned multifaceted potential of CAP for pain management, most evidence remains at the preclinical stage except for injury healing and nerve damage restoration. Thus, translating this potential into the clinical practice not only requires large‐scale, multicohort clinical trials to validate the proposed mechanisms of action, but also requires the conquer of several challenges.

First, though CAP can be administered via direct device ejection, its limited penetration depth (about 2 mm [496, 497, 498]) substantially constrains its therapeutic applicability, as many clinical pain syndromes originate from deep‐seated locations. Current research has focused primarily on enhancing its delivery efficacy through methods such as microneedles and micro‐sized tube [499, 500, 501, 502]. As the future endeavor, one could overcome this limitation by developing formulations suitable for subcutaneous injection, leveraging CAP's ability to deliver reactive species in alternative states such as liquids or hydrogels. Alternatively, materials engineered to preserve CAP's reactive species could be established to further expand its utility to topical applications. Additionally, CAP could be formulated as a nasal spray to enable delivery to the brain through intranasal administration.

Second, the efficacy of CAP is highly dose dependent [434]; consequently, tailoring dosages to individual patient conditions and achieving precise control over treatment dosing to ensure desirable outcomes present significant challenges. While this challenge is widely acknowledged, current studies still rely on empirical experience for dosage selection across different medical contexts. This reliance stems partly from the absence of a standardized dosage definition and partly from a limited understanding of the dynamic cellular redox profiles during both the pathogenesis and treatment of relevant diseases, including pain. A potential solution involves identifying key biomarkers or evaluation indices that reflect the redox status and pathogenic state of a particular disease and developing corresponding detection technologies that are rapid, user‐friendly, and capable of dynamic monitoring.

### 7.2 Hippocampus as a Potential Sensor for Nociplastic Pain

#### 7.2.1 Conceptual Framework

While nociceptors, specialized sensory endings of first‐order neurons, serve as the sensors of nociceptive pain by detecting actual or potential tissue damage, and neuropathetic pain originates from malfunctioning nerve fibers themselves due to lesions or diseases within the somatosensory system, nociplastic pain arises from maladaptive CNS processing, characterized by amplified pain perception without clear tissue or neural pathology. Thus, while nociceptive pain depends on peripheral sensor activation, both neuropathic and nociplastic pain reflect pathophysiological changes beyond nociceptor function.

The hippocampus and hypothalamus, deeply intertwined neural structures, govern essential functions from memory consolidation to physiological homeostasis. Scientifically, the hippocampus encodes high‐dimensional cognitive maps and episodic experiences, while the hypothalamus translates these signals into adaptive neuroendocrine and autonomic responses. Some theoretical frameworks, inspired by concepts like the Akashic Records, speculatively propose that such brain systems might interface with higher‐dimensional information fields, where past, present, and potential experiences exist as accessible data structures. In this metaphor, the hippocampus may act as a nociplastic receiver of experiential traces, the hypothalamus as a modulator of embodied meaning of signals received (not limited to nociplastic pain), and consciousness itself as a process navigating a vast, implicit order of information, far beyond conventional spacetime descriptions. Thus, can we add hippocampus into the primary pain framework as the sensor of nociplastic pain, where nonciceptors sense signals from our known three‐dimensional space and hippocampus detect information from higher dimensional space? These multimodal signals converge on the hypothalamus, which functions as a central decoding hub for interoceptive and affective‐relevance assessment. This integrated information is then relayed via third‐order neurons to higher cortical regions, where it culminates in the conscious experiences including chronic pain. From this perspective, both nociceptive and nociplastic pain can be understood as conditions arising from dysfunctional sensory mechanisms. While nociceptors act as receivers encoding information from physical three‐dimensional space, hippocampus may serve a analogous role in connecting us with higher‐dimensional, metaphysically oriented experiences (Figure 6). In contrast, neuropathic pain can be viewed primarily as a pathology of abnormal signal transduction within the nervous system.

This, once holds true, may have transformative clinical potential. For instance, it may guide our future investigations on markers (imaging, molecular, or genetic biomarkers) characteristic of hippocampus structural or functional abnormalities for diagnosing nociplastic pain, as well as innovative therapeutic strategies directly targeting altered hippocampus and disrupted signal transduction between hippocampus and hypothalamus.

#### 7.2.2 Supportive Evidence

In support of this hypothesis, current research on nociplastic pain has increasingly recognized its CNS system origins and the involvement of higher brain regions like the hippocampus and hypothalamus, with the contemporary research status being characterized by several convergent findings and persistent gaps. First, there is robust evidence linking hippocampal abnormalities to chronic and nociplastic pain states. It has been demonstrated that chronic pain may lead to structural and functional alterations in the hippocampus, including neuronal atrophy in the CA1 and CA3 regions, reduced synaptic plasticity, and decreased functional connectivity [503]. Specifically, in chronic pain models, the ventral hippocampal CA1 exhibited dysfunctional neuronal excitability and altered molecular signaling, such as the downregulation of hyperpolarization activated cyclic nucleotide gated potassium and sodium channel 2 (HCN2), which was implicated in pain chronicity [504]. These changes have been directly associated with the cognitive and affective comorbidities of pain, such as Alzheimer's disease [505]. Second, the hypothalamus has been established as a critical integrator for the physiological and affective dimensions of pain. Its role in coordinating autonomic responses, such as cardiovascular changes, to different types of nociceptive input through intricate circuits involving the PAG has been confirmed [506, 507, 508, 509]. These functions have positioned the hypothalamus as a key node in translating noxious signals into holistic homeostatic and emotional responses. Third, the quest for objective biomarkers for pain, especially nociplastic pain, has become a major research frontier. Innovations are being developed, focusing on composite, multimodal strategies that integrate neuroimaging (like MRI to detect hippocampal volume changes), molecular biomarkers (like cytokines or metabolites), neurophysiological data, and genetic profiling to improve diagnosis and enable personalized therapy [510, 511, 512, 513].

#### 7.2.3 Translational Challenges

Despite the much evidence in support of this hypothesis, substantial limitations exist that may preclude the formal adoption of the hippocampus as a “sensor of nociplastic pain” analogous to peripheral nociceptors. The primary limitation is the lack of direct causal evidence. While hippocampal alterations are consistently correlated with chronic pain, it is not definitively established whether these changes are the cause of nociplastic pain generation or a consequence of prolonged nociceptive input, stress, or other comorbid factors. On the other hand, the field lacks specific biomarkers that can reliably distinguish nociplastic pain from other central sensitivity syndromes or from pain with mixed mechanisms, rendering the theoretical proposition of the hippocampus accessing “higher‐dimensional” information fields falling outside the scope of testable scientific hypotheses with current available methodologies. Finally, translating these concepts into targeted therapies is challenging. Although modulating hippocampal plasticity or hypothalamus‐linked pathways is a promising research direction, developing precise, effective, and safe interventions based on this specific framework requires a much deeper understanding of the causal pathways and their human applicability, which current research has not yet achieved.

In conclusion, the exploration of CAP and the hippocampus‐as‐sensor model represents a bold departure from conventional pain paradigms. CAP emerges as a uniquely versatile physical agent with preclinical and early clinical evidence supporting its efficacy across pain‐related mechanisms, from wound healing and neuroinhibition to comorbidity management (Figure 5). Concurrently, the hippocampal hypothesis provides a provocative, mechanism‐driven framework for understanding nociplastic pain, shifting the focus from the periphery to central cognitive‐affective processing. Despite the supportive scientific evidence provided, the substantial clinical promise is, however, tempered by several translational barriers that must be surmounted before either can achieve routine clinical adoption. For CAP, key challenges include overcoming its limited tissue penetration, establishing precise and personalized dosing protocols, and validating efficacy through large‐scale clinical trials. For the hippocampal model, the primary obstacles are establishing direct causal evidence for its role in pain generation and developing testable biomarkers and interventions based on this framework. Despite these challenges, rigorously investigating these innovative concepts is crucial, as they hold the potential to catalyze the development of entirely new, personalized, and mechanism‐based therapeutic avenues for the most refractory forms of chronic pain.

## 8 Limitations

This review synthesizes current knowledge on the epidemiology, mechanisms, risk factors, diagnosis, and management of the three principal mechanistic categories of chronic pain, nociceptive, neuropathic, and nociplastic, and proposes two novel conceptual frameworks: CAP as a multifactorial therapeutic approach and the hippocampus as a putative central sensor for nociplastic pain. By adopting a mechanism‐oriented perspective, the discussion underscores the necessity of moving beyond purely syndromic diagnoses toward a more nuanced understanding that informs targeted, personalized treatment strategies.

However, several critical limitations of the present review must be acknowledged. First and foremost, this is a narrative review, not a systematic review or meta‐analysis. While it provides a comprehensive synthesis, its findings are inherently influenced by the authors’ selection and interpretation of the literature, introducing the potential for selection bias. Certain areas may have been emphasized over others based on the authors’ perspective and the overarching goal of introducing the CAP and hippocampus frameworks. Thus, though we advocated CAP, it does not mean that CAP necessarily outweighs more established therapeutics and exclude the existence of other promising therapeutic avenues. Instead, CAP may serve as an ideal adjuvant, enhancing therapeutic efficacy and mitigating adverse effects when combined with other modalities. Also, although we proposed the hippocampus model, it does not negate the functional importance of other brain regions. Areas such as the PFC (involved in cognitive regulation), the amygdala (contributing to emotional processing), and the default mode network (integrating self‐referential information) may collectively support hippocampal functionality, forming a dynamic network that synthesizes sensory, affective, and cognitive signals to fully represent the multidimensional nature of the pain experience.

Second, the review's structure, which categorizes pain into three distinct types, risks oversimplifying the clinical reality. In practice, many patients present with overlapping or mixed pain mechanisms, and the boundaries between nociceptive, neuropathic, and nociplastic pain are often blurred. While the review acknowledges this continuum, the segregated discussion of mechanisms, risk factors, and treatments might inadvertently reinforce a compartmentalized view. Future work may be needed to further integrate these models to better reflect the clinical complexity.

In conclusion, the landscape of chronic pain management is evolving from a symptom‐focused approach to a mechanism‐informed strategy. This review highlights the distinct yet interconnected pathways of nociceptive, neuropathic, and nociplastic pain and explores innovative therapeutic and conceptual frontiers. The limitations inherent in its narrative nature and the preliminary status of its key proposals underscore the need for continued research. Future efforts should prioritize systematic, mechanistic studies in well‐phenotyped patient cohorts, the development of objective diagnostic tools to differentiate pain types, and rigorous clinical testing of novel interventions like CAP. Validating integrative models, such as the proposed hippocampal role in nociplastic pain, could ultimately pave the way for a more profound understanding and more effective, personalized treatment of this debilitating global health challenge.

## 9 Conclusion and Prospects

The modern understanding of chronic pain has undergone a profound transformation, shifting from a monolithic view of persistent symptoms to a sophisticated, mechanism‐based taxonomy of nociceptive, neuropathic, and nociplastic subtypes. This review has synthesized the distinct yet often overlapping pathophysiological landscapes of these conditions, spanning from peripheral sensitization and central hyperexcitability to profound brain circuit remodeling and dysregulated stress axes. Concurrently, we have detailed the corresponding evolution in the clinical practice, where diagnostic frameworks increasingly integrate clinical phenotyping with advanced biomarkers and where therapeutic strategies have moved decisively from a one‐size‐fits‐all approach to multimodal, personalized regimens tailored to the dominant pain mechanism. This paradigm shift, while foundational, has exposed the persistent limitations of our current tools, that is, diagnostic imprecision, therapies with modest long‐term efficacy and significant side‐effect burdens, and a frequent inability to address root causes, particularly in nociplastic pain, highlighting an urgent need for transformative innovation.

To address these gaps, this review has introduced and critically evaluated two forward‐looking conceptual frontiers. First, CAP has emerged as a uniquely versatile physical modality. Its therapeutic potential is presented as particularly promising due to its apparent capacity to interact with several key pathophysiological pathways across different pain types. That is, instead of being confined to a single pathway, it possesses a multimodal action profile capable of promoting tissue healing, attenuating neuroinflammation, modulating key comorbidities like obesity and cancer, and interacting with endocrine signaling. This has positioned CAP as a potential therapeutic platform that could simultaneously target several risk factors and mechanisms across different chronic pain subtypes. Second, we proposed a novel neurobiological framework that posits the hippocampus as a putative central “sensor” for nociplastic pain. This hypothesis reframes nociplastic pain not merely as a failure of modulation but as a dysfunction of a higher‐order sensory system, where the hippocampus potentially interfaces with maladaptive cognitive‐emotional information streams, and the hypothalamus acts as a hub for interoceptive and affective decoding. This model provides a compelling, albeit hypothetical, mechanism to explain the diffuse, affective, and memory‐laden nature of nociplastic pain, offering new targets for intervention. Additionally, by offering a fresh neuroanatomical and functional model to explain the pervasive somatic and affective symptoms that characterize nociplastic pain, it shifts the focus from a purely “bottom‐up” sensitization model to one incorporating “top‐down” cognitive‐emotional valuation of bodily states, potentially explaining the high comorbidity with psychological distress and cognitive dysfunction.

However, the journey from promising concept to clinical reality is paved with significant translational challenges. For CAP, the path forward requires overcoming key technological and biological hurdles, that is, enhancing its limited tissue penetration through novel delivery systems (e.g., injectable hydrogels), establishing precise, biomarker‐guided dosing protocols to navigate its biphasic (pro‐ vs. antioxidant) effects, and most critically, validating its efficacy and safety through rigorous, large‐scale randomized controlled trials across defined pain populations. For the hippocampal model, the primary obstacle is moving beyond correlation to causation. Future research must employ advanced causal interrogation techniques (e.g., optogenetics in animal models, longitudinal neuroimaging in prodromal human cohorts) to definitively establish whether hippocampal dysfunction drives nociplastic pain initiation or is merely a secondary consequence. This work must be coupled with the development of objective, hippocampus‐based diagnostic biomarkers to translate the theory into a clinically actionable tool.

Ultimately, the future of chronic pain management lies at the convergence of precision medicine and paradigm‐shifting innovation. The integration of deep phenotypic profiling with multiomics data will be crucial for refining our mechanistic classifications and predicting treatment response. Within this refined framework, disruptive approaches like CAP and novel circuit‐targeted therapies informed by models such as the hippocampal sensor hypothesis hold the potential to move beyond symptomatic management. The goal must evolve from merely suppressing pain to actively rectifying the dysregulated neural, immune, and endocrine processes that sustain it. By embracing these integrative and innovative strategies, the field can progress toward truly effective, durable, and personalized solutions, fundamentally altering the prognosis for millions living with chronic pain.

## Author Contributions

X. Dai conceived the idea and and prepared the draft and figures. C.X. Wang and X. Dai prepared the tables. X.P. Mei approved the content of the study. P. Jiang provided the financial support. All authors have read and approved the final manuscript.

## Conflicts of Interest

The authors declare no conflicts of interest.

## Ethics Statement

The authors have nothing to report.

## Funding

This study was supported by the Clinical Research Award of the First Affiliated Hospital of Xi'an Jiaotong University, China (No. XJTU1AF2022LSK‐39). The funding bodies played no role in the design of the study and collection, analysis, and interpretation of data and in writing the manuscript.

## Data Availability

The authors have nothing to report.
